# Effectiveness of nutrition counseling for pregnant women in low‐ and middle‐income countries to improve maternal and infant behavioral, nutritional, and health outcomes: A systematic review

**DOI:** 10.1002/cl2.1361

**Published:** 2023-11-29

**Authors:** Omar Dewidar, Jessica John, Aqeel Baqar, Mohamad Tarek Madani, Ammar Saad, Alison Riddle, Erika Ota, Jacqueline K. Kung'u, Mandana Arabi, Manoj Kumar Raut, Seth S. Klobodu, Sarah Rowe, Jennifer Hatchard, Jennifer Busch‐Hallen, Chowdhury Jalal, Sara Wuehler, Vivian Welch

**Affiliations:** ^1^ Bruyere Research Institute University of Ottawa Ottawa Ontario Canada; ^2^ Eat, Drink and Be Healthy Tunapuna Trinidad and Tobago; ^3^ School of Epidemiology and Public Health University of Ottawa Ottawa Ontario Canada; ^4^ Global School of Nursing Science, Global Health Nursing St. Luke's International University Chuo‐ku Japan; ^5^ Nutrition International—Africa Regional Office Nairobi Nairobi Kenya; ^6^ Nutrition International New York New York USA; ^7^ Nutrition International New Delhi India; ^8^ Department of Nutrition and Food Science California State University, Chico Chico California USA; ^9^ Nutrition International Ottawa Ontario Canada; ^10^ Global Technical Services, Nutrition International Ottawa Ontario Canada

## Abstract

**Background:**

Nutritional counseling, which includes two‐way interactive education, has been hypothesized to improve the health and nutritional status of pregnant women, but little is known about the impact such practice of care might have on maternal and infant health and behavioral outcomes of pregnant women living in low income, low‐middle income, and upper‐middle‐income countries (LMIC)s.

**Objectives:**

We conducted a systematic review to appraise the effectiveness and impact on health equity of two‐way nutritional counseling practices in LMICs on maternal and infant behavioral, nutritional, and health outcomes.

**Search Methods:**

We conducted electronic searches for relevant studies on Medline, Embase, CINAHL, PsychInfo, and the Cochrane CENTRAL for randomized and non‐randomized trials on the effectiveness of two‐way interactive nutritional counseling among pregnant women from the date of database inception up to June 22, 2021. In addition, we searched references of included studies in systematic reviews, gray literature resources, and unpublished studies or reports that satisfied our eligibility criteria using a focused Google search.

**Selection Criteria:**

We included randomized and non‐randomized controlled studies (NRS), controlled before and after, and interrupted time series that assessed the effectiveness of two‐way interactive nutrition counseling targeting pregnant women in LMICs.

**Data Collection and Analysis:**

Data extraction and risk of bias were conducted in duplicate. The risk of bias (ROB) for randomized trials (RCT) was assessed according to the Cochrane Handbook of Systematic Reviews, and ROB for NRS was assessed using the Newcastle‐Ottawa scale (NOS). RCT and NRS were meta‐analyzed separately.

**Main Results:**

Our search identified 6418 records and 52 studies met our inclusion criteria, but only 28 were used in the quantitative analysis. Twenty‐eight studies were conducted in Asia, the most in Iran. Eight studies were conducted in Africa. Two‐way interactive nutritional counseling during pregnancy may improve dietary caloric intake (mean difference [MD]: 81.65 calories, 95% confidence interval [CI], 15.37–147.93, three RCTs; *I* ^2^ = 42%; moderate certainty of evidence using GRADE assessment), may reduce hemorrhage (relative risk [RR]: 0.63; 95% CI, 0.25–1.54, two RCTs; *I* ^2^ = 40%; very low certainty of evidence using GRADE assessment), may improve protein (MD: 10.44 g, 95% CI, 1.83–19.05, two RCTs; *I* ^2^ = 95%; high certainty of evidence using GRADE assessment), fat intake (MD: 3.42 g, 95% CI, −0.20 to 7.04, two RCTs; *I* ^2^ = 0%; high certainty of evidence using GRADE assessment), and may improve gestational weight gain within recommendations (RR: 1.84; 95% CI, 1.10–3.09, three RCTs; *I* ^2^ = 69%). Nutrition counseling probably leads to the initiation of breastfeeding immediately after birth (RR: 1.72; 95% CI, 1.42–2.09, one RCT). There was little to no effect on reducing anemia (RR: 0.77; 95% CI, 0.50–1.20, three RCTs; *I* ^2^ = 67%; very low certainty of evidence using GRADE assessment) risk of stillbirths (RR: 0.81; 95% CI, 0.52–1.27, three RCTs; *I* ^2^ = 0%; moderate certainty of evidence using GRADE assessment) and risk of cesarean section delivery (RR: 0.96; 95% CI, 0.76–1.20, four RCTs; *I* ^2^ = 36%; moderate certainty of evidence using GRADE assessment).

**Authors’ Conclusions:**

Our review highlights improvements in maternal behavioral and health outcomes through interactive nutrition counseling during pregnancy. However, we are uncertain about the effects of nutrition counseling due to the low certainty of evidence and a low number of studies for some key outcomes. Moreover, the effects on health equity remain unknown. More methodologically rigorous trials that focus on a precise selection of outcomes driven by the theory of change of nutrition counseling to improve maternal and infant behavioral and health outcomes and consider equity are required.

## 1 PLAIN LANGUAGE SUMMARY

### 1.1 Nutrition counseling may improve some maternal and infant health and behavioral nutrition outcomes, but more high‐quality studies are needed

A balanced diet during pregnancy is vital for women's health, positive pregnancy outcomes, and proper fetal growth and development. The World Health Organization (WHO) recommends nutrition counseling as part of regular antenatal care contacts to improve nutrition practices and health outcomes.

Two‐way interactive nutrition counseling involves discussions and shared decision‐making between patient and provider. Our review found that this approach for nutrition counseling has a positive effect on some maternal outcomes but not others.

### 1.2 What is this review about?

Poor maternal nutrition is prevalent among pregnant women in low‐ and middle‐income countries (LMICs). Nutrition deficiencies are strongly associated with maternal and infant health complications. This review aims to identify and synthesize the evidence for the effects of nutrition counseling on pregnant women living in LMICs on maternal infant and child behavioral, nutritional, and health outcomes.

**What is the aim of this review?**

This Campbell systematic review examines the effectiveness of two‐way interactive nutrition counseling for pregnant women in LMICs on improving maternal and infant health and behavioral outcomes and its impact on health equity.

### 1.3 What studies are included?

Eligible studies had to be randomized controlled trials (RCTs), non‐randomized controlled studies, controlled before‐after studies, or interrupted time series studies evaluating the impact of nutrition counseling compared to standard care among pregnant women with no pregnancy‐related complications living in LMICs.

Fifty‐two studies were included in the review. Of these, 28 reported outcomes of interest. All the studies compared nutrition counseling to standard antenatal care. Studies were conducted mainly in Asia (28 studies) and Africa (8 studies).

### 1.4 What are the main findings of this review?

Nutrition counseling may improve dietary caloric intake, protein and fat intake, and gestational weight gain within health recommendations and may reduce hemorrhage during post‐delivery.

Little to no effect was found in reducing the risk of cesarian delivery or improving hemoglobin concentration.

Nutrition counseling shows little to no difference in reducing the rate of stillbirths and anemia.

No studies reported the effects of nutrition counseling on maternal mortality, iron deficiency, or assessed iron intake.

### 1.5 What do the findings of this review mean?

Our review highlights improvements in maternal behavioral and health outcomes through interactive nutrition counseling during pregnancy. However, inconsistencies in the definition of nutrition counseling studies conducted in LMICs and low certainty of evidence limit our interpretation of the findings. Thus, high‐quality studies with a standardized definition of nutrition counseling that highlights the interactive nature of the practice and a clear theory of change for maternal and infant behavioral and health outcomes are needed.

Findings for impact on health equity were scarce and uncertain.

### 1.6 How up‐to‐date is this review?

The review authors searched for studies up to June 2021.

## 2 BACKGROUND

### 2.1 The problem, condition, or issue

A healthy, balanced diet is necessary for a positive pregnancy experience. Pregnant women have the right to quality antenatal care (ANC). Evidence shows that fetal growth and maternal physical and mental health during pregnancy require increased nutritional demands (Mousa et al., 2019). Nutritional deficiencies are highly prevalent among pregnant women, specifically in low‐ and middle‐income countries (LMICs), contributing to preventable adverse maternal and birth outcomes (Darnton‐Hill & Mkparu, 2015; Viswanathan et al., 2008). Observational studies have indicated that gestational weight gain and energy intake are strongly associated with better birth outcomes, especially in undernourished women (Kramer, 1987; Kramer et al., 2008; Rush, 2001). Additionally, micronutrient deficiencies affect the maternal capacity to conceive and support the pregnancy through birth (Gernand et al., 2016). Substantial evidence exists to support that nutrition counseling, in the presence or absence of other communication channels and tools, carries the potential to improve nutrition practices and health outcomes, in part, through health education and promotion (Bhutta et al.,  2008; Bhutta et al., 2013; Graziose et al., 2018; Mbuagbaw et al., 2015; Tekelab et al., 2019; World Health Organization, 2016).

The World Health Organization recommends “Counseling on healthy eating and physical activity” to be integral to women's ANC (World Health Organization, 2016). Despite the potential impact of nutrition counseling, limited time, infrastructure, staff capacity, and motivation often hinder or prevent the delivery of quality ANC in low‐resource settings—and often, challenges in the delivery of nutrition counseling occur (Girard & Olude, 2012). Nutrition counseling provides a critical communication channel between the patient and healthcare provider to identify the patient's nutritional problems, needs and goals, and ways to achieve them. However, the setting where the counseling is conducted may impact the effectiveness of the practice. For example, many evidence‐based nutrition actions achieve the desired outcomes when women and mothers use a recommended nutrition practice at home (Fox et al., 2018). Furthermore, if a supportive enabling environment is in place (e.g., if maternal micronutrient supplements are available at distribution points in adequate quantities and quality, supportive health care policies are in place, and there is adequately compensated and distributed health staff), then quality nutritional counseling with beneficiaries has been shown to improve provider job satisfaction, retention, and ability to provide nutrition services to a higher quality standard (Girard & Olude, 2012; Sunguya, Poudel, Mlunde, Shakya, et al., 2013; Sunguya, Poudel, Mlunde, Urassa, et al., 2013). This, in turn, can affect maternal and infant health outcomes. A better understanding of the current coverage of interactive nutrition counseling during ANC in LMICs can help better target resources and advocacy for accelerated progress toward the Sustainable Development Goals (SDGs).

Pregnant women in LMICs are at an increased risk of facing unjust or unfair health disparities, especially during the critical time of their pregnancy. The systematic social disadvantage associated with living in low‐resource settings jeopardizes the quality of the ANC they receive. As well, health inequities, differences in health that are avoidable and unjust (Whitehead, 1992), could be further magnified by the disadvantages they experience due to their place of residence, race and culture, occupation, gender and sex, religion, education, socioeconomic status, social capital, plus: personal characteristics (i.e., age, disabilities), relationship features (i.e., exclusion from school, parent drug use), and time‐dependent relationships (i.e., times when an individual might be temporarily disadvantaged such as pregnancy). Such characteristics are better known using the acronym PROGRESS+, which provides a useful framework to identify differences in health outcomes among socially disadvantaged populations (O'Neill et al., 2014).

### 2.2 The intervention

Nutrition counseling practices have varied considerably (Vasiloglou et al., 2019). Therefore, we defined the intervention based on the World Food Program (Programme, 2014 *)*: “Counseling provided to individuals or in group sessions, that includes two‐way interactive education linked to promoting specific behaviors.” There is growing interest in identifying the effectiveness of nutrition counseling during pregnancy on antenatal and postpartum women's health and behavioral outcomes, and neonates’ health outcomes. Thus, this review investigates the effectiveness of identified two‐way interactive nutrition counseling practices at improving maternal and infant nutrition and health in LMICs.

### 2.3 How the intervention might work

**Figure 1:**
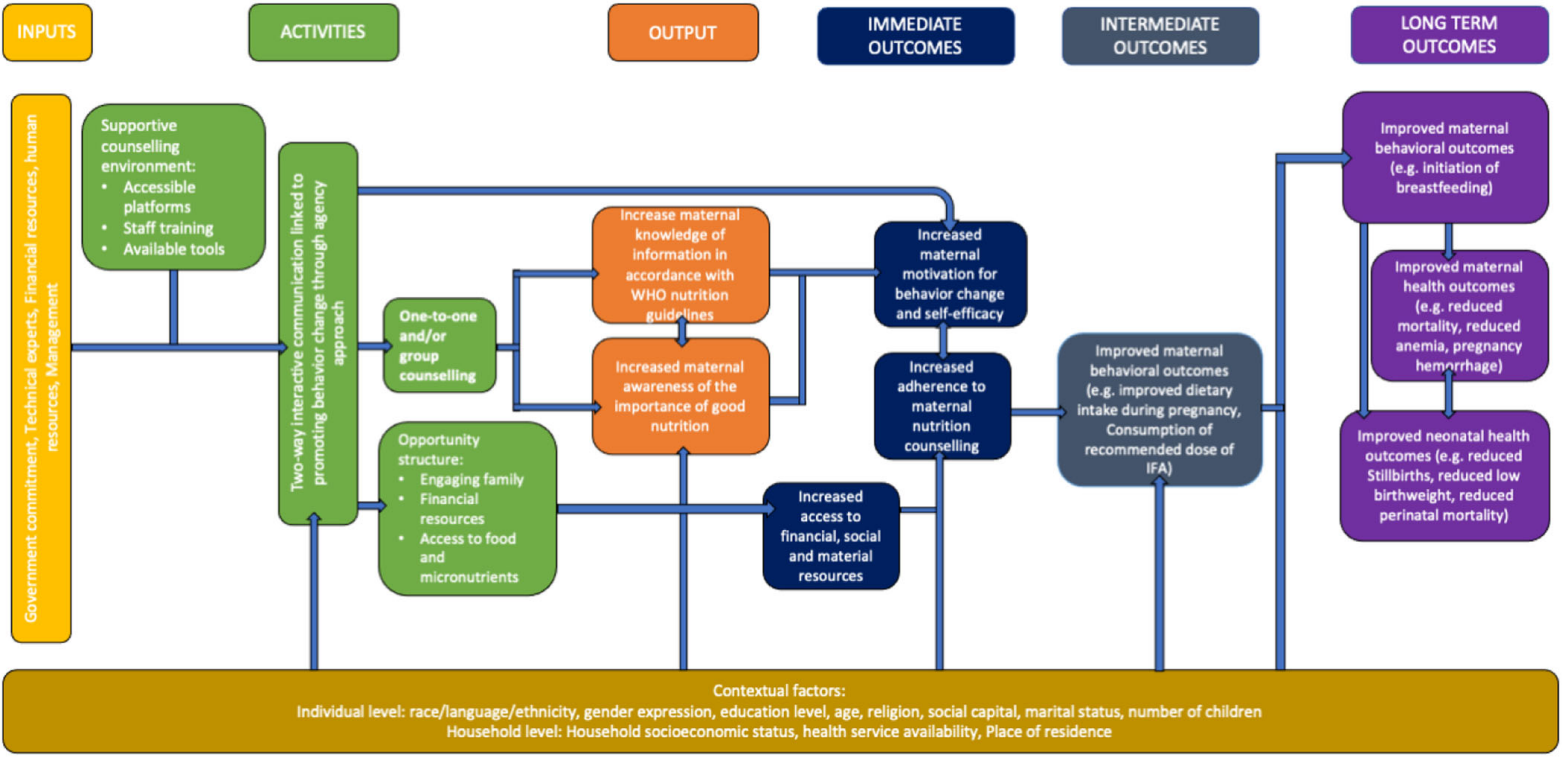
Logic model: Nutrition counseling to improve maternal and infant behavioral and health outcomes. Figure adapted from Nutrition International's *Quality Nutrition Counseling in Antenatal Care*: *What Is It and What is Needed?* poster presented at the 2020 Micronutrient Forum Conference.

Figure 1's Logic Model summarizes key components of interest on the path from nutrition counseling to desirable outcomes. This model formed the framework for selecting indicators and their analyses while recognizing that published research might only collect and report on certain components of interest. Women's empowerment is positively associated with improved health and nutrition outcomes for women and infants (Carlson et al., 2015; Cunningham et al., 2015; Pratley, 2016). Knowledge, awareness, motivation, and access are desirable outputs and outcomes to support women's empowerment. Thus, nutrition counseling that incorporates an empowerment approach, with a minimum of two‐way interactive education, may improve intervention uptake and effectiveness by increasing women's agency to act on the information provided through nutrition counseling sessions. In turn, the program can create a supportive opportunity structure (Alsop, 2005) where women can access the necessary material, financial and social support to effect positive change. We have adapted the empowerment model used by Riddle et al. to reflect the causal pathways through which an empowerment approach may contribute to improved health and nutrition outcomes for pregnant women and their infants. We adopt the definition of empowerment developed by Kabeer: “The expansion in people's ability to make strategic life choices in a context where this ability was previously denied to them” (Kabeer, 1999). We aimed to collect the characteristics of the counseling programs to determine elements of an effective structure for nutrition counseling.

As part of this review, we looked for evidence of nutrition counseling that applied a complete empowerment model. We defined a complete empowerment model as including two components as outlined in Riddle et al.: (1) Nutrition counseling to foster agency and (2) activities to create a supportive opportunity structure.

First, nutrition counseling that fosters agency should provide participants with a “space for self‐reflection and identification of important life areas” in relation to their nutritional status (Shankar et al., 2019). In other words, desirable counseling provides opportunities for participants to identify and assess barriers to improving their nutritional situation and identifying goals and opportunities to improve their nutritional status. In turn, this is expected to increase their motivation for behavior change and self‐efficacy. Second, activities to create a supportive opportunity structure will provide participants with the necessary material, social or financial resources needed to act on the knowledge and skills they acquire through nutrition counseling (Alsop, 2005; Kabeer, 1999). This includes altering the constraining political, economic, socio‐cultural, intrafamilial, or legal structures (both formal and informal) that can limit behavior change (Alsop, 2005; Mahotra Anju & Boender, 2002). Examples of opportunity structure‐related activities are engaging participants’ families in nutrition counseling to increase their awareness and support for proper nutrition during pregnancy or providing financial support to participants to increase their access to food or health services.^1^

#### 2.3.1 Knowledge and awareness, outputs

Nutrition‐related health education embedded in nutrition counseling to pregnant women can help reduce the risk of developing nutritional‐specific complications throughout pregnancy, during labor and delivery (Girard & Olude, 2012). The common characteristic of such counseling lies in the provision of direct or indirect communication focusing on influencing their knowledge about the importance of a nutritious and balanced diet during pregnancy, and improving their behaviors and attitudes toward the use of necessary nutritional supplements such as fortified foods and micronutrient supplements when appropriate (Arrish et al., 2014). Nutrition counseling for pregnant women also seeks to influence their behavior with regard to accessing quality ANC services, promoting delivery at the health facility, accessing quality postnatal care services, and adequate intent and young child feeding practices (Alam et al., 2005; Girard & Olude, 2012; Perumal et al., 2013).

#### 2.3.2 Empowerment‐based counseling

We hypothesize that nutrition counseling during ANC conducted through home visits, one‐on‐one facility counseling, and/or interactive group meetings with community and facility‐based health personnel while covering the importance of maternal nutrition, including improving food quality and micronutrient supplementation would increase the women's knowledge of nutrition‐related information according to international practice guidelines. Similarly, the frequency (dose) of patient interactions is expected to affect the effectiveness of the intervention. Trained providers must engage with women and provide this information in an interactive fashion. In an empowerment‐based model, the interaction would provide space for women to identify their own nutritional goals and articulate strategies for their achievement, that is, counseling accompanied by nutritional supplements. In turn, women would gain awareness of the importance of prenatal and postpartum nutrition, which is integral to all counseling programs, and feel motivated to act on the information they have obtained. Peer support in the form of key influencers such as grandmother, husband, and other family members and friends in the counseling sessions’ group sessions are anticipated to improve adherence to the actions addressed through counseling sessions by creating a supportive opportunity structure for pregnant women to exercise agency over their nutritional needs. Thus, we hypothesized that nutrition counseling which involves key influencers, would lead to increased motivation and resource support for behavior change, which would translate into improved attitudes and practices, such as increased adherence to the nutrition counseling intervention(s). We also hypothesized that this effect would be more pronounced with multiple opportunities for nutrition education and counseling to take place.

#### 2.3.3 Change in behavior

In the intermediate term, these immediate outcomes would improve behavioral outcomes such as dietary intake and diversity during pregnancy and adherence to micronutrient supplementation. Change in behavior is critical on the path to impact. Nutrition counseling for pregnant women should also seek to influence their behavior with regard to accessing quality ANC services, promoting delivery at the health facility, accessing quality postnatal care services, and adequate intent and young child feeding practices (Alam et al., 2005; Girard & Olude, 2012; Perumal et al., 2013).

#### 2.3.4 Long‐term outcomes

Nutrition counseling is anticipated to improve maternal and infant health outcomes thereby contributing to reduced risk of mortality, pregnancy complications, anemia, stillbirths, and perinatal mortality.

#### 2.3.5 Contextual factors or mediating variables

We acknowledge there are also several factors at an individual and household level that could mediate the effectiveness of the intervention, as well as the suggested outcomes along the causal pathway (Pouchieu et al., 2013). These include a household's socioeconomic status, food security, or an individual's level of education. We applied the PROGRESS+ framework (O'Neill et al., 2014) to identify these socially stratifying factors and where possible, conducted sub‐group analyses to look at the differences in effects by group.

### 2.4 Why it is important to do this review

There is limited evidence on the effectiveness of nutritional education and counseling among pregnant women. A Cochrane systematic review found that such practices tended to lower the risk of preterm delivery, but had no effect on infant complications or stillbirth (Ota et al., 2015). A similar meta‐analysis of controlled experimental trials found a significant association between receiving nutritional counseling and improved gestational and birth weight, as well as reduced risk of anemia among pregnant mothers (Girard & Olude, 2012). Even though both reviews have shown that nutritional education and counseling carry the potential to positively influence the health status of both the mother and the infant, several methodological limitations in the breadth of their searches (no gray literature or equity terms searched) and selection of study designs prevented a comprehensive synthesis of knowledge around the effectiveness of such practices. To the best of our knowledge, our systematic review provides the most robust and equity‐focused analysis on the effectiveness of nutritional education and two‐way interactive counseling among pregnant women in LMICs for health and nutrition outcomes that were analyzed by the previous review as well as an additional analysis of behavioral outcomes.

Nutrition counseling and education have increased the compliance of pregnant women to dietary guidelines in high income countries (Goodarzi‐Khoigani et al., 2018). The impact of such interventions on maternal and fetal health outcomes in LMICs has not been well examined or documented. Moreover, little evidence is available on the implications that such interventions might carry on the health equity of pregnant women in such socially and economically disadvantaged settings represented by PROGRESS+ characteristics. The added value of our systematic review is to not only provide a comprehensive analysis of the effectiveness of nutrition counseling and education interventions, but also to use an equity lens in interpreting the differences in the magnitude of their effectiveness across participant characteristics. This review aimed to accelerate the progress toward SDGs set by the United Nations (Girard & Olude, 2012). Although the focus of this review was on maternal nutrition and newborns, it also investigated an interdependent relationship of gender equality/socioeconomic status with nutrition. Thus, according to our pathway, we hoped to improve equity in access to social, financial resources for women. Our review will help inform nutrition counseling programmers and policy makers about effective and equitable counseling programs to improve the health status of pregnant women and their infants in countries where malnutrition is highly prevalent.

## 3 OBJECTIVES

The objective of this systematic review was to identify, appraise, and synthesize the best available evidence on the effectiveness of two‐way interactive nutrition counseling on maternal and infant health outcomes, and assess the differences in effects across participants’ PROGRESS+ characteristics.

To achieve these objectives, we aimed to answer the following research questions:

- What is the effect of maternal nutrition counseling during pregnancy in LMICs on maternal, infant and infant behavioral and health outcomes?
- What are the documented potential effects of nutrition counseling interventions on maternal and infant behavioral and health outcomes across participants’ PROGRESS+ characteristics?

## 4 METHODS

### 4.1 Criteria for considering studies for this review

#### 4.1.1 Types of studies

This review was conducted according to a published protocol (Dewidar et al., 2021). We conformed to the Cochrane Collaboration's Effective Practice and Organisation of Care (EPOC) criteria for the selection of studies (EPOC, 2017) as randomized trials may not be sufficiently available to address the effects of two‐way nutrition counseling on health outcomes. We included individual and cluster randomized controlled trials (RCTs), non‐randomized controlled trials (NRCTs), controlled before and after (CBA) studies as well as interrupted time series or repeated time measures studies (ITSs). We followed EPOC guidance and only included ITS with three data points before and three data points after the intervention (EPOC, 2017). We excluded cross sectional studies. Studies that did not include a control group were excluded from the review as it was difficult to attribute causation with that study design.

#### 4.1.2 Types of participants

Participants included low risk pregnant women (15–49 years, with no active pregnancy‐related complications that required referral for additional management or specialist care) in different stages of gestation in LMICs, as defined by the World Bank country income group categories at the time of the study's conduct.

#### 4.1.3 Types of interventions

The objective of this systematic review was to identify, appraise and synthesize the best available evidence on the effectiveness of nutrition counseling in improving maternal and infant health outcomes, and assess the differences in effect sizes across participants’ PROGRESS+ characteristics.

The primary comparison was interactive nutrition counseling versus standard ANC with no interactive nutrition counseling. Studies that compared nutrition counseling to another approach of ANC (i.e., educational booklets given to pregnant women) were included and analyzed separately.

Support *could be* provided in addition to counseling as follows:

1. Providing nutritious food to malnourished individuals, based on anthropometric entry and exit criteria (low body mass index or low mid‐upper arm circumference).
2. Providing additional nutrient supplementation (e.g., micronutrient supplements such as iron folic acid supplements).
3. Educating individuals on a variety of activities that aimed to give them tools to meet their basic needs, including food, so that they do not have to rely on long‐term income transfers or food assistance.

The support must have been present in both study arms for the study to be included as we aimed to assess the efficacy of nutrition counseling.

#### 4.1.4 Types of outcome measures

Studies reporting at least one of the following outcomes were included:

**Table:** 

	Primary outcomes	Secondary outcomes
Maternal health	Mortality (up to 6 weeks postpartum)	Gestational weight gain (kg)
	Anemia (hemoglobin lower than 110 g/L) during and post counseling	Hemoglobin concentration postintervention
	Iron deficiency (as defined by study authors)	Hemorrhage (defined by study authors)
	‐	Mode of delivery (c‐section vs. vaginal)
Maternal behaviors	Intent to breastfeed (defined by study authors)	Dietary intake during pregnancy (kcal/day)
	Timely initiation of breastfeeding (proportion of women who initiated breastfeeding within 1 h of birth)	Macronutrient intake during pregnancy
	Adherence to iron containing supplement consumption (proportion of women who reportedly consumed iron supplements during pregnancy as defined by study authors)	‐
	Adherence to ANC (as reported by study authors)	‐
Infant health	Stillbirths (death after 20 week's gestation and before birth)	Low birthweight (less than 2500 g)
	Preterm birth (before 37 week's gestation)	Small for gestational Age (SGA) (as defined by study authors)
	Perinatal mortality (defined by study authors)	‐

We also recognized that some outcomes would be defined differently by different studies. For example, infant death was defined by mortality within first 4 weeks after birth, but some studies used a different definition. Similarly, adherence was defined by participation in all counseling sessions or as participation in a minimum number of sessions. We collected details on the definition of all outcomes and decided whether the construct being measured was sufficiently similar, based on clinical expertise, to be included in meta‐analysis.

#### 4.1.5 Impact of intervention on health equity

To assess the impact of nutrition counseling on health inequalities, we examined the effects of nutrition counseling across socially stratifying factors, if they are reported by authors. We used the acronym PROGRESS+ to identify characteristics which may lead the population to being socially disadvantaged. PROGRESS is short for: Place of residence, Race/ethnicity/language, Occupation, Gender/Sex, Religion, Education, Socioeconomic status, and Social capital/resources, personal characteristics (i.e., age, disabilities), relationship features (i.e., exclusion from school, parent drug use) and time‐dependent relationships (i.e., leaving the hospital or other times when an individual might be temporarily disadvantaged (O'Neill et al., 2014). We reported the findings of all these subgroup analyses as conducted within the studies. When there was data on subgroup analyses across the same PROGRESS‐Plus factor for more than one study, we combined them using meta‐analysis to assess across study effects.

#### 4.1.6 Other eligibility criteria

Language and date of publication were not restrictive criteria for our review. We translated studies identified in non‐English languages. We also included protocols, peer‐review conference abstracts and studies in gray literature in our review and they were classified as awaiting classification.

Adaptations to the protocol were discussed with team members, documented and reported as a discrepancy from the protocol in the systematic review.

### 4.2 Search methods for identification of studies

#### 4.2.1 Electronic searches

We searched the following electronic bibliographic databases for relevant records: Medline via Ovid, Embase via Ovid, PsychInfo via Ovid, CINAHL via EBSCO, and the Cochrane CENTRAL Register of Controlled Trials via Ovid from date of database conception (Medline 1946, EMBASE 1974, PsychInfo 1967, CENTRAL 1996, and CINAHL 1961) to June 22, 2021. Furthermore, we hand‐searched reference lists of included studies and all relevant reviews identified by our search to ensure literature search saturation. We sought consultations from content experts in the fields of nutritional counseling and health literacy in LMICs for any missing records (AR, EO, MA, SR, JH, JBH, SW). Moreover, we searched PROSPERO for any registered systematic reviews that have been recently published and hand‐searched their reference lists for relevant records. Finally, we searched electronic registries of clinical trials such as clinicaltrials.gov and the WHO International Clinical Trials Registry for any recently published trials not captured by our search.

A comprehensive search strategy was developed in consultation with a health science librarian with expertise in systematic review searches and was adapted to the syntax and subject headings of each of the electronic databases that we planned to search. A combination of indexed terms, database‐specific and MeSH headings, as well as free text keywords were used. Please see Supporting Information: Appendix I for our search strategy. Keywords used to develop our search strategy include variations of the following: “Nutrition,” “Counseling,” “Education,” “Program,” “Communication,” “Diet,” “prenatal/perinatal care,” “Nutrition therapy,” and “Pregnant women.”

We filtered out any editorials, comments, or personal communications to ensure that we only captured peer‐reviewed trials on our topic of interest.

#### 4.2.2 Searching other resources

We further scanned references and citations of included studies and relevant systematic reviews for primary studies that met our eligibility criteria. We also used a focused google search to identify relevant non‐peer reviewed studies or reports. Furthermore, we searched the following gray literature sources:

- International Food Policy Research Institute (https://www.ifpri.org/)
- Alive and Thrive website (https://www.aliveandthrive.org/en)
- World Health Organization e‐Library of Evidence for Nutrition Actions (e‐LENA) (https://www.ennonline.net/)
- Sight and Life Library (https://sightandlife.org/)

### 4.3 Data collection and analysis

#### 4.3.1 Description of methods used in primary research

A broad range of intervention designs were expected to be used as part of the studies identified in this review. They included home or clinic visits, group or individual counseling sessions and a variety of intervention content.

#### 4.3.2 Selection of studies

Two review authors (AB, AS, OD or JJ) independently screened records yielded by our search against our inclusion/exclusion criteria using their titles and abstracts. To do so, we used Covidence reference manager software (*Covidence systematic review software*). Subsequently, eligible records were screened as full text, in duplicate and independently, to evaluate if they truly meet our inclusion criteria. Discrepancies between reviewers were resolved by consensus or with the help of a third member of the research team (VW) when required. We prepared a PRISMA study selection chart (Moher et al., 2009) along with references for excluded studies for transparent reporting.

#### 4.3.3 Data extraction and management

Data extraction was conducted independently and in duplicate. Conflicts were resolved by consensus or with the help of a third member of the research team (VW) when required. Also, if the authors of primary studies need to be contacted about missing information, they were reached out to by their primary contact information for a maximum of three attempts without reply in between. A standardized data extraction framework was developed in consultation with content and health equity experts using excel sheets. Please see Supporting Information: Appendix II for a data extraction sheet. To ensure the validity of our data extraction framework and increase its compatibility with our analysis objectives, we pilot tested the extraction process with a random sample of *n* = 5 included records and revised the process accordingly.

Reviewers extracted the following variables:

(1) Study identifiers: such as name of authors, date of publication, journal, volume, and page number if needed
(2) Study methodology: objectives, study design, methodological details such as processes for randomization, allocation and blinding, target population, recruitment and sampling procedures, setting, participant eligibility criteria, participant baseline characteristics, sample size per arm at baseline
(3) Intervention description: name, nature, components (e.g. timing, frequency/dose, route of delivery, empowerment approach elements), and details of the comparison intervention
(4) Outcomes: Definitions, instrument and scale interpretation, timing of outcome measures, and adverse events
(5) Results: Participant attrition rate, categorical data, continuous data, between‐group estimates
(6) Author conclusions, funding and conflict of interest.

The data extraction form emphasized the separate extraction of health and behavioral outcomes to ensure consistency with data reporting.

Throughout data extraction we assessed whether outcome data is stratified by PROGRESS+: Place of residence, Race and culture, Occupation, Gender and sex, Religion, Education, Socioeconomic status, Social capital, personal characteristics (i.e., age, disabilities), relationship features (i.e., exclusion from school, parent drug use), and time‐dependent relationships (i.e., leaving the hospital or other times when an individual might be temporarily disadvantaged) (O'Neill et al., 2014).

#### 4.3.4 Assessment of risk of bias (ROB) in included studies

Risk of bias of individual studies was assessed by two independent reviewers at the study level using R.O.B 2.0 tool. At the study level, the Cochrane collaboration tool for assessing ROB was used when assessing bias for RCTs (JPT et al., 2019). The domains included sequence generation, allocation concealment, selective reporting, blinding of participants/personnel/outcome, selective outcome reporting, and incomplete outcome data. All biases were assessed by providing a judgment (high, low, some concerns) on individual elements from the five domains. In the likelihood of assessing ROB in non‐randomized studies of interventions, the Newcastle‐Ottawa Scale (NOS) tool was used to assess the ROB in non‐randomized studies (Wells et al., 2014). This tool was used to assess any biases in the selection of participants, comparability of cohorts, and adequacy of outcome assessment. All judgments of biases were made independently by two reviewers. Disagreements were resolved through discussion or consulting with the study supervisor (VW). To provide a graphic representation of bias between and within studies, we used robvis tool in R (McGuinness & Higgins, 2021). Modified EPOC ROB were used to critically appraise interrupted time series and CBAs (EPOC, 2017). This tool assessed protection against contaminations, recruitment bias, and detection of analysis errors in studies by cluster allocation instead of individuals. To minimize ROB in individual studies, experimental studies were given priority over observational studies to prevent subjective analysis and interpretation.

#### 4.3.5 Measures of treatment effect

Within each of our outcomes of interest, we assessed heterogeneity across the type of intervention to decide if it is sensible to pool the data together. We conducted all possible meta‐analyses of effect estimates for RCTs and NRCTs separately while accounting for differences in outcomes, interventions and comparators. ITS studies were analyzed using the methods recommended by EPOC (EPOC, 2017). Continuous outcomes, such as iron deficiency, anemia/hemoglobin concentration, or birthweight, were analyzed using mean differences in change from baseline if possible. In the availability of baseline and end‐point data, we calculated the change from baseline and associated standard difference as provided in the Cochrane handbook for systematic reviews of Interventions (JPT et al., 2019). All continuous outcomes were accompanied by estimates of statistical significance such as standard deviations, standard errors of the mean, and 95% confidence intervals. Dichotomous outcomes, such as the rate of stillbirths, perinatal and infant mortality, were analyzed using relative risk measurements such as risk ratios (RR). Similarly, all dichotomous effect estimates were accompanied by statistical significance estimates such as 95% confidence intervals (95% CI) and *p* values. When more than one publication reported effect estimates from the same population receiving the same intervention, we reported effect estimates that encompass a larger sample size.

#### 4.3.6 Unit of analysis issues

For cluster randomized trials (CRT), we assessed unit of analysis errors (e.g. no adjustment for clusters made). If errors were detected, we inflated the standard deviation using an Intra‐cluster Correlation Coefficient (ICC) in accordance with the Cochrane handbook for systematic reviews of interventions (JPT et al., 2019). In the presence of dichotomous outcomes, we adjusted the numerator and denominator for unit of analysis errors.

#### 4.3.7 Criteria for determination of independent findings

For studies with multiple arms, we selected all arms that fill the inclusion criteria and still provided a “control” arm. In turn, we analyzed each arm in comparison to the control arm separately. If more than one arm provided two‐way interactive communication with the participants and there was no control remaining, we assigned the intervention arm as the arm that is considered to have the most interactive two‐way communication with the participants. The control arm would be the arm considered to have the least interactive two‐way communication with the participants. The unit of analysis was per individual randomized in the study.

#### 4.3.8 Dealing with missing data

Whenever data from included primary studies were not reported or lacking (e.g., missing details on variation such as standard deviation, number of participants) or missed details on PROGRESS+ factors, we contacted authors to obtain such missing data. Three attempts were made to contact the corresponding authors for missing data with 3 working days between the attempts. If authors did not respond or were not able to provide missing data, we reported effect estimates as reported only. Values and standard deviations for missing data were not imputed. Unavailable standard deviations were calculated using other methods such as confidence intervals and exact p values using the formulae provided in the Cochrane handbook for systematic reviews of Interventions (JPT et al., 2019). Studies with no quantitative results were not included in our analysis.

For continuous outcomes, we used intention to treat analysis method, using the number of individuals randomized into the study, including missing individuals. For dichotomous outcomes, we also used intention to treat when analyzing our data, thus the total number of participants in the study was used as the denominator, assuming that the event did not occur in the missing individuals (Higgins et al., 2019). Sensitivity analysis was conducted when primary studies report per‐protocol analyses.

#### 4.3.9 Assessment of heterogeneity

Statistical heterogeneity was assessed using the *I* ^2^ statistic and *χ* ^2^ test of independence.

#### 4.3.10 Assessment of reporting biases

Funnel plots were used to assess the risk of publication bias in our analyses with 10 or more studies to prevent biasing the estimated between‐study heterogeneity variance (JPT et al., 2019). We used RevMan 5.4 software to visually create the funnel plots (*Review Manager (RevMan) [Computer program]. Version 5.4. Copenhagen*: The Nordic Cochrane Centre, The Cochrane Collaboration, 2020).

#### 4.3.11 Data synthesis

Since we are assessing two‐way interactive counseling, we expect that the delivery of the intervention would differ on an individual basis depending on the woman's needs. Therefore, we used random effect models in our analysis. All pooled results were reported using forest plots and pooled effect estimates. We did not pool data from randomized and non‐randomized study designs.

#### 4.3.12 Subgroup analysis and investigation of heterogeneity

We planned to conduct subgroup analyses for maternal health, infant health and maternal behavioral outcomes across the following:

- Frequency (dose) or type of intervention (web‐based interaction, one‐on‐one or group)
- Specific equity characteristics across the PROGRESS+ criteria (Education, socioeconomic status and age are considered the most important for this question)
- Time of commencement of the intervention by gestational age (potentially contributes to dose)
- Empowerment model or approach (based on our classification described in the following section)

We tested for subgroup interaction in Review Manager 5.4. We documented if any of these subgroups were conducted within the studies and report them in our review.

*I* ^2^ value with a cut‐off of 0.75 or higher was considered for subgroup based on any clinically important differences in study populations, characteristic of interventions, nature of comparator groups, and outcome measurements (JPT et al., 2019).

##### Empowerment approach classification

We classified included studies by the extent to which they apply a complete empowerment model. Included studies were classified according to the following categories: (1) Complete empowerment model (i.e., including agency and opportunity structure activities), (2) Partial empowerment model (i.e., including agency‐related activities only), or (3) Unclear empowerment approach. Classification decisions were based on the primary author's description of the intervention.

Where there was a sufficient number of included studies for any of our outcomes of interest, we conducted the following sub‐group analyses: (1) complete empowerment model versus unclear empowerment approach, (2) complete empowerment model versus partial empowerment approach, (3) partial empowerment model versus unclear empowerment approach. If there are too few studies for sub‐group analysis, we provided a narrative synthesis comparing the effects across the three categories.

#### 4.3.13 Sensitivity analysis

Sensitivity analysis was conducted across ROB (generation of sequence, allocation bias and protection against contamination) and for methodological imputations (e.g., adjustment for unit of analysis errors).

#### 4.3.14 Summary of findings and assessment of the certainty of the evidence

We tabulated outcome measures in GRADE summary of findings (SoF) tables aggregated in the following categories: Maternal health outcomes, infant and health outcomes, Maternal behavioral outcomes with seven outcomes per SoF table. The table was generated as per recommendations in the Cochrane Handbook and included:

1) Primary and secondary outcomes of the review
2) Measures of absolute magnitude of intervention effect
3) Number of participants
4) Grade of the overall quality of the body of evidence
5) Comments that aid the interpretation of the results

Certainty of evidence was evaluated using GRADE methodology (Atkins et al., 2004) to assess our certainty in the evidence. We present our certainty levels as either high, moderate, low or very low. The results of each outcome measure were assessed against eight criteria. The following five criteria were considered for possible downgrading the quality of evidence: study quality (ROB), consistency (consistency between included studies), precision of results, directness (same population, intervention and outcomes as we desire) and reporting bias. Three criteria may upgrade the level of certainty: strength of associations between intervention and outcome; size of the dose–response effects; and where all plausible confounders would have reduced the effect. We expected that there might be challenges with pooling of results. If this occurred, we planned to report the rating of the certainty of evidence using a narrative summary using the GRADE approach (Murad et al., 2017).

Conclusions were based on the findings reported in this study only. We described implications of the findings for both policy and research, while highlighting possible limitations and remaining uncertainties, and identifying current gaps in the evidence.

## 5 RESULTS

### 5.1 Description of studies

#### 5.1.1 Results of the search

**Figure 2:**
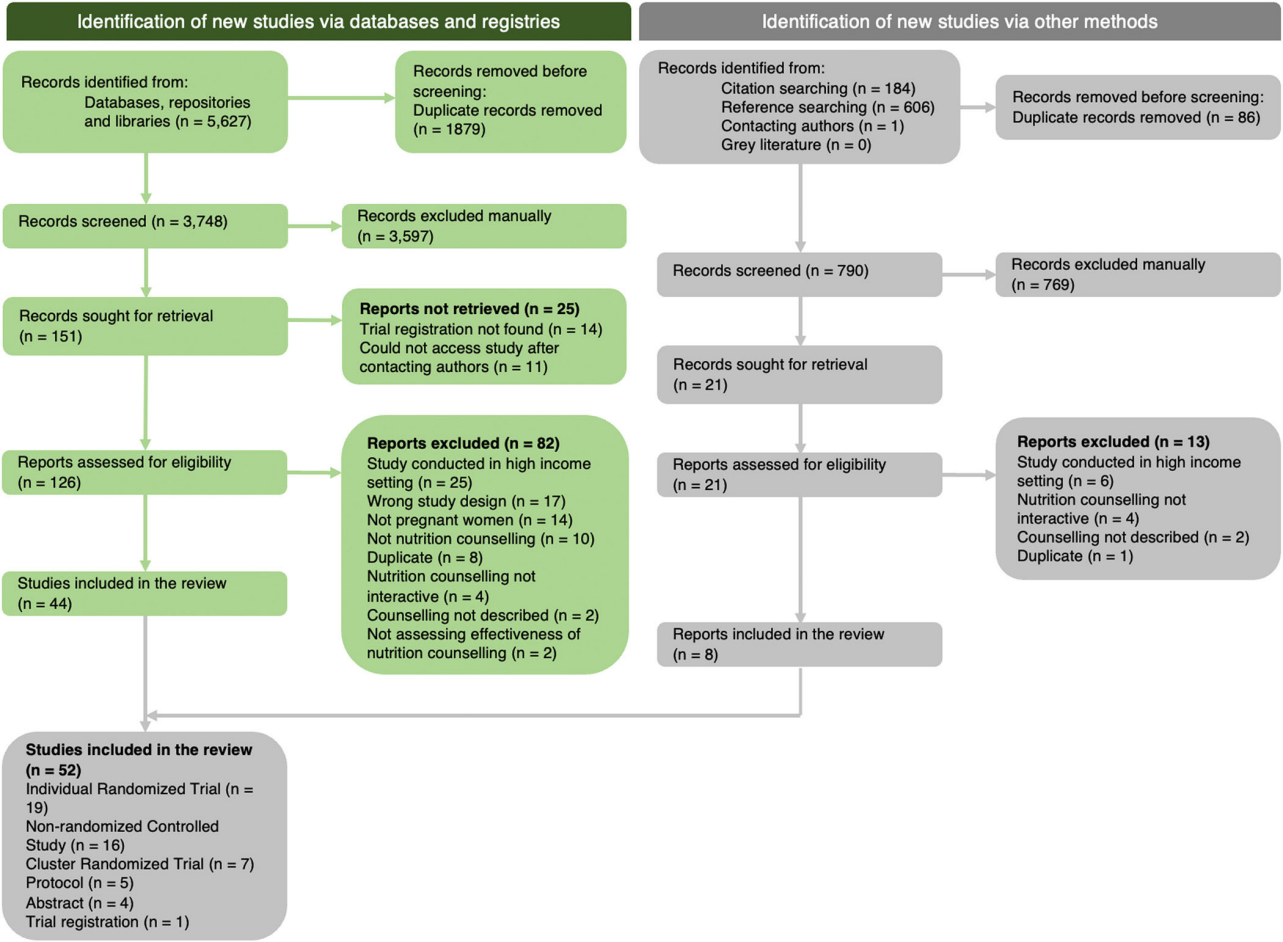
PRISMA flowchart.

Figure 2 displays the PRISMA chart summarizing the results of the search. We identified 6418 papers from electronic reference searching, citation checking, and manual Google searches. After removing duplicates, 4538 titles and abstracts were reviewed. Of those, 147 full texts were assessed for eligibility, identifying 52 included studies (42 studies, five protocols, four abstracts, and one trial registration). Gray literature sources were searched using keywords listed in the search strategy. However, no relevant reports were found.

#### 5.1.2 Included studies

We included a total of 42 studies for nutrition counseling to improve maternal and infant outcomes. Nineteen studies were RCTs (Abdel‐Aziz et al., 2018; Asiabar et al., 2018; Aşcı & Rathfisch, 2016; Belizan et al., 1995; Goodarzi‐Khoigani et al., 2018; Izadirad et al., 2017; Jahan et al., 2014; Jahangiri et al., 2020; Jing et al., 2015; Kanber et al., 2011; Khoramabadi et al., 2015; Kian et al., 2016; Lin et al., 2020; Liu et al., 2009; Mazloomy‐Mahmoodabad et al., 2020; Mohsenzadeh‐Ledari et al., 2020; Tu et al., 2021; Vítolo et al., 2011; Zhou & Tang, 2020), 16 were NRS (Abu‐Baker et al., 2021; Daniel et al., 2016; Garg & Kashyap, 2006; Hasneezah et al., 2020; Kamalifard et al., 2012; Liu et al., 2017; Malta et al., 2021; Mirmolaei et al., 2009; Nahrisah et al., 2020; Perichart‐Perera et al., 2009; Permatasari et al., 2021; Saadatnia et al., 2021; Sachdeva & Mann, 1994; Sharifirad et al., 2013; Sunuwar et al., 2019; Villalon et al., 2010) and 7 were CRTs (Demilew et al., 2020; Isrctn, 2017; Kafatos et al., 1989; Katenga‐Kaunda et al., 2021; Nyamasege et al., 2021; Nyamasege et al., 2018; Ziyenda Katenga‐Kaunda et al., 2020). All the studies compared two‐way interactive nutrition counseling with standard ANC. However only 66% (28 out of 42 studies) reported at least one of the outcomes of interest (Abdel‐Aziz et al., 2018; Aşcı & Rathfisch, 2016; Belizan et al., 1995; Daniel et al., 2016; Demilew et al., 2020; Garg & Kashyap, 2006; Hasneezah et al., 2020; Isrctn, 2017; Jahan et al., 2014; Jing et al., 2015; Kafatos et al., 1989; Kanber et al., 2011; Kian et al., 2016; Lin et al., 2020; Liu et al., 2017; Mohsenzadeh‐Ledari et al., 2020; Nahrisah et al., 2020; Nyamasege et al., 2021; Nyamasege et al., 2018; Perichart‐Perera et al., 2009; Sachdeva & Mann, 1994; Sharifirad et al., 2013; Sunuwar et al., 2019; Tu et al., 2021; Villalon et al., 2010; Vítolo et al., 2011; Zhou & Tang, 2020). Seven studies were not in English language: four were in Persian (Isrctn, 2017; Kamalifard et al., 2012; Mazloomy‐Mahmoodabad et al., 2020; Mirmolaei et al., 2009), one in French (Villalon et al., 2010), one in Portuguese (Villalon et al., 2010) and one in Turkish (Kanber et al., 2011).

##### Outcomes

More than half of the studies (23 out of 42 studies, 55%) focused on maternal health outcomes (Abdel‐Aziz et al., 2018; Aşcı & Rathfisch, 2016; Belizan et al., 1995; Daniel et al., 2016; Garg & Kashyap, 2006; Hasneezah et al., 2020; Jahan et al., 2014; Jing et al., 2015; Kafatos et al., 1989; Kanber et al., 2011; Kian et al., 2016; Lin et al., 2020; Liu et al., 2009; Liu et al., 2017; Mohsenzadeh‐Ledari et al., 2020; Nahrisah et al., 2020; Nyamasege et al., 2018; Sharifirad et al., 2013; Sunuwar et al., 2019; Tu et al., 2021; Villalon et al., 2010; Vítolo et al., 2011; Zhou & Tang, 2020), 15 studies (36%) reported maternal behavioral outcomes (Aşcı & Rathfisch, 2016; Belizan et al., 1995; Demilew et al., 2020; Garg & Kashyap, 2006; Hasneezah et al., 2020; Isrctn, 2017; Jahan et al., 2014; Jing et al., 2015; Liu et al., 2009; Nahrisah et al., 2020; Nyamasege et al., 2021; Nyamasege et al., 2018; Sachdeva & Mann, 1994; Sunuwar et al., 2019) and 13 studies (31%) reported infant health outcomes (Abdel‐Aziz et al., 2018; Aşcı & Rathfisch, 2016; Belizan et al., 1995; Daniel et al., 2016; Jahan et al., 2014; Kafatos et al., 1989; Liu et al., 2009; Liu et al., 2017; Nahrisah et al., 2020; Nyamasege et al., 2021; Nyamasege et al., 2018; Perichart‐Perera et al., 2009; Villalon et al., 2010). One study reported outcomes disaggregated by place of residence (urban vs. rural) (Liu et al., 2009). Studies that reported outcomes are summarized in Tables 1, 2, 3, 4.

**Table 1:** Description of nutrition counseling in studies that reported outcomes of interest.

Paper	Definition of nutrition counseling						Outcomes of Interest
	How is Nutrition Counseling defined in the paper	Type (1‐1, group etc.)	Frequency (# of times in ANC)	Who provided the counseling	Where the counseling was provided	Content (diet, nutrition supplements, breastfeeding etc.)	
Abdel‐Aziz et al. (2018)	Not reported—Tailored nutrition counseling sessions were designed based on the 1990 IOM guidelines for nutrition and weight during pregnancy. Women in the intervention group received standard care delivered at the center group and attended six extra counseling sessions with the nutrition counselor, with face‐to‐face appointments every 2 weeks during the implementation phase.	1‐1	11–15	member of the research team	ANC centers	How to control weight gain during pregnancy and how to maintain or optimize a healthy lifestyle in a period of physical and mental changes. the nutrition counselor educated participants how to choose healthier foods (whole grains, fruits and vegetables, healthy fats, and protein sources); how to limit intake of unhealthy foods (refined grains and sweets) and beverages (sugary drinks); and how to get rid of the unhealthy habits (frying food, eating fast food, skipping meals, and eating unhealthy snacks between meals) and avoid sedentary life by encouraging walking for 30 min three times per week	Anemia, gestational weight gain, mode of delivery, preterm birth
Belizan et al. (1995)	Not reported—prenatal care (standard hospital procedures) was supplemented with four to six home visits.	1‐1	4–6	Specially trained female social workers	Home visits	reducing stress and anxiety, inadequate health‐related behavior, and untimely or null recognition of pregnancy‐ and labor‐related morbidity and at increasing health services utilization.	Anemia, hemorrhage, adherence to nutrition counseling, stillbirths, Low birthweight
Nyamasege (2018)	An interactive supporting process focusing on the need for diet modification, is a widely used strategy in health facilities to improve the nutritional status of women during pregnancy	1‐1	7	Trained community health volunteers	Home visits	The specific maternal nutrition education key messages included importance of adequate diet during pregnancy, attending ANC, and taking iron and folate supplements. Other maternal health‐related key messages were on seeking early treatment for infections and how to prevent them, encouraging the use of good hygienic practices, avoiding alcohol, smoking, and nonprescription drugs, and good antenatal care.	Mode of delivery, adherence to nutrition counseling, preterm birth, low birthweight
Jahan et al. (2014)	Not reported—The nutrition education sessions were provided in a counseling format and focused on encouraging behavior change.	group	3	Trained member of the research team	Maternal health hospital	The content of the sessions included the nutritional value of food, the importance of exclusive breastfeeding, establishing an adequate diet during pregnancy and lactation, cooking practices for optimum retention of nutrients, and creating awareness about food taboos relating to pregnancy and infant feeding.	Gestational weight gain, timely initiation of breastfeeding
Jing et al. (2015)	Not reported—women in the intervention group received an education manual on diet and physical activity (written by the research team) after randomization, and one‐to‐one counseling for at least 20 min in a private room with a trained graduate student (W. J.), By contrast, pregnant women in the control group received only conventional interventions, such as standard health education manuals produced by the hospital	1‐1 & group	3	Graduate student	West China Second University	The key points of education included the harm of GWG and GDM, the benefit of encouraged behaviors, the difficulty to change habits, and the importance of belief in the efficacy of the interventions.	Gestational weight gain, Macrionutrient intake during pregnancy
Sharifirad (2013)	Not reported—Then the interview based on HBM was performed in two sessions of nutritional education (16 minuets for each session) according to nutrition educational guidelines in health care centers and live lecture, group discussion, colloquy and individual nutrition consultation for experimental group.	Group	2	female researcher	Health care centers	Not reported according to the “nutrition educational Guidelines in health care centers”	Gestational weight gain
Villalon et al. (2010)	Not reported	Group	5	Not reported	Prenatal clinics	individual nutritional advice including a personalized diet adapted to the needs of each woman and educational group sessions. Various subjects were taught such as physiological transformations related to pregnancy, recommended diet, essential nutrients, body weight, breastfeeding, the introduction of solid foods to the child and diet the nursing woman.	Gestational weight gain
Kafatos et al. (1989)	Not reported	1‐1	6–10	Trained nurse counselors	Clinics	Basics of nutrition during pregnancy for the health of mother and fetus, including food sources and nation effects of selecting a balanced diet. The women were also taught practical techniques for improving the quality of their diets. They were encouraged to consume locally grown foods that have a high nutrient value and to prepare and preserve food in such a way as to reduce the loss of nutrients.	Hemoglobin, dietary intake, stillbirths, low birthweight, small for gestational age (SGA)
Kanber et al. (2011)	Not reported	1‐1	One time	Not reported	Obstetrics and Gynecology Hospital and the University Hospital, Istanbul Turkey	Not reported	Hemoglobin concentration
Lin et al. (2020)	Structured but individually modified education regarding a balanced dietary pattern, moderate physical activity, and weight control.	1‐1	One time	Interventionist (no further detail)	Women and Children's Hospital, School of Medicine, Xiamen University, China	The balanced dietary pattern in the intervention was based on the China diagnosis and therapy guideline of pregnancy with diabetes mellitus,24 aimed to achieve or maintain ideal body weight and meet nutritional needs. Pregnant women were encouraged to consume vegetables, fruits, high‐fiber wholegrain products, low‐fat dairy products, and to avoid foods rich in sugar and saturated fatty acids, among other guidance	Gestational weight gain, Hemorrhage, Mode of delivery
Liu et al. (2009)	Not reported‐ The goals of the intervention were to provide information and guidance on contemporary postpartum practices and take away common misconceptions about traditional dietary and health behaviors (e.g., fruit and vegetables should be restricted because of cold nature, postpartum women should stay inside and not go outdoors)	Group	2	Trained health staff by the team	Clinic	The content of the two sessions focused on:	Mode of delivery, dietary intake during pregnancy (kcal/day)
						Food guide pyramid knowledge Nutrient‐food association knowledge The importance of dairy consumption, fruit and vegetable consumption Examples of healthy menus Optimal hygiene behavior and physical exercise patterns during postpartum period Discussions about healthy lifestyle during postpartum period Common misconceptions on hygiene behaviors during puerperium Discussions about nutrition and health problems concerns postpartum practices	
Zhou & Tang (2020)	Not reported—individualized nutritional care	1‐1	One time	Experts from the Nutrition Department, West China Second University Hospital, Sichuan University/West China Women's and Children's Hospital	Hospital	The daily caloric requirement in the first trimester and the second and third trimester, in which protein accounted for 25%, carbohydrates accounted for 55% and fat accounted for 20% of the die, while consuming 5–6 meals a day. Adherence to low salt and low sugar diets.	Hemorrhage
Kian et al. (2016)	Not‐reported—face‐to‐face training	1‐1 & group	2	Tehran University of Medical Sciences	Hospital	Training items mentioned in the first session of the training were included; a definition of gestational diabetes, causes, side effects, individuals at risk, and control and treatment of gestational diabetes such as glycemic control. Training items that were taught in the second session were included; nutrition, physical activity and exercise, insulin injection, and following‐up after the pregnancy.	Mode of delivery

Abbreviations: ANC, antenatal care; GDM, gestational diabeties mellitus; GWG, gestational weight gain.

**Table 2:** Summary of studies that reported maternal health outcomes.

Maternal health outcomes of interest	Paper	Inclusion criteria	Exclusion criteria	What trimester was the maternal health outcome measured? (i.e., anemia, gestational weight)	What was the nutrition intervention? (i.e., counseling on Macronutrient intake, counseling on IFA)	What was the change from the intervention (unit: effect size)
Anemia	Abdel‐Aziz et al. (2018)	primigravidae aged between 20 and 30 years in the first trimester (<12 weeks of gestation) of selected ANC clinic, free from history of any chronic medical problems	younger than 18 years, have a history of previous abortion or stillbirth, presence of any chronic disease, and taking any type of medications that might interfere with the bodyweight (steroids, diuretics, and thyroid hormones).	Third trimester	Prevention of excessive GWG, healthy eating	RR: 0.45 [0.22, 0.93]
	Belizan et al. (1995)	Women initiating prenatal care between the fifteenth and twenty‐second weeks of gestation with singleton pregnancies;, with at least one of the following risk factors: (1) previous low‐birth‐weight or infant death; (2) previous fetal, neonatal or infant death (3) <17 years old; (4) body weight < 50 kg and height < 1.5; (5) low family income defined by locally adapted cutoff points; (6) <3 years of schooling; (7) smoking or heavy alcohol consumption; and (8) single, separated, divorced or widowed.	With clinical evidence of cardiovascular, renal, or other chronic diseases; history of cerclage. Rh‐negative, or mental diseases	Third trimester	healthy eating, support during pregnancy	RR: 1.03 [0.85, 1.25]
	Nyamasege (2018)	Pregnant women were prospectively included throughout the trimesters, residents of two densely populated slums (Korogocho, and Viwandani) located 7 km apart from each other.	Women of reproductive age who were to deliver before the intervention started	Third trimester	Knowledge of pregnancy complications, importance of taking iron and folate supplements.	RR: 0.72 [0.45, 1.17]
Gestational weight gain (kg)	Aşcı & Rathfisch (2016)	Pregnant women aged over 18, who had no health problems, did not intend to lose weight in pre‐pregnancy period, got pregnant in natural ways for two times at most, and were pregnant for a period of 3 months or less, were included in the study	Pregnancy complications	Third trimester	Pregnancy weight gain, healthy eating	MD (kg): 0.16 [−1.75, 2.07]
	Jahan et al. (2014)	Women at a gestational age of 24 weeks attending the government Maternal and Child Health Training Institute, Azimpur, and the Marie Stopes Clinic, Bashbari, Dhaka	Complications and special requirements	Third trimester	Pregnancy weight gain, adequate food intake, and breastfeeding for the newborn	MD (kg): 1.03 [−1.79, 3.85]
	Jing et al. (2015)	Women aged at least 18 years, could understand the written Chinese language, and did not have pre‐existing diabetes	pregnancy‐related complications or general medical disorders not associated with pregnancy	Second trimester	Pregnancy weight gain	MD (Kg): −0.45 [−1.40, 0.50]
Gestational weight gain (within recommendations)	Sharifirad (2013)	Pregnant women who were resided in Gonabad and went to urban health care centers for prenatal care	‐	Third trimester	No information reported	RR: 1.92 [1.37, 2.71]
	Villalon et al. (2010)	Women were between 19 and 30 years old, first month of gestation, attended one of the prenatal clinics in the Cotonou region, did not consume any form of tobacco, never experienced a previous pregnancy.	‐	Third trimester	Healthy eating, pregnancy weight gain, breastfeeding	RR: 1.35 [0.38, 4.76]
Hemoglobin concentration	Kafatos et al. (1989)	Women residing in the market towns and villages of Florina, Greece	‐	Third trimester	Healthy eating	MD (mmol/L): −0.05 [−0.05, −0.05]
	Kanber et al. (2011)	Women in 3rd month of pregnancy	Pregnant women with chronic anemia, chronic kidney disease, parasitic disease, and illiterate people were not included in the study.	Third trimester	No information reported	MD (mmol/L): 0.10 [−0.39, −0.59]
Hemorrhage	Belizan et al. (1995)	Women initiating prenatal care between the 15th and 22nd weeks of gestation with singleton pregnancies;, with at least one of the following risk factors: (1) previous low‐birth‐weight or infant death; (2) previous fetal, neonatal or infant death (3) <17 years old; (4) body weight < 50 kg and height < 1.5; (5) low family income defined by locally adapted cutoff points; (6) <3 years of schooling; (7) smoking or heavy alcohol consumption; and (8) single, separated, divorced or widowed.	With clinical evidence of cardiovascular, renal, or other chronic diseases; history of cerclage. Rh‐negative, or mental diseases	Third trimester	Healthy eating, support during pregnancy	RR: 0.82 [0.51, 1.31]
	Lin et al. (2020)		Pregnant women with pre‐existing diabetes mellitus, multiple pregnancies, use of medication that influences glucose metabolism (e.g., steroids, b‐adrenergic agonists, and anti‐psychotic drugs), physical disability, or severe psychiatric disorders.	Second or third trimester of pregnancy	Healthy eating, exercise	RR: 0.26 [0.10, 0.66]
	Zhou & Tang (2020)	According to the diagnostic methods and standards of IADPSG 2010, fasting plasma glucose (FPG) ‚ ≥5.1 mmol/L when first examined at 24–28 weeks of pregnancy could be diagnosed as GDM.	(1) Twin or multiple pregnancies; (2) combined with other metabolic diseases such as thyroid dysfunction, abnormal secretion of adrenaline or adrenocorticotropic hormone or growth hormone; (3) combined with other diseases.	Postpartum (after delivery)	No information reported	RR: 0.29 [0.06, 1.31]
Cesarean section delivery	Abdel‐Aziz et al. (2018)	Primigravidae aged between 20 and 30 years in the first trimester (<12 weeks of gestation) of selected ANC clinic, free from history of any chronic medical problems were recruited to participate.	Women were not eligible for participation if they were younger than 18 years (to avoid natural linear growth), having a history of previous abortion or stillbirth, presence of any chronic dis‐ease, and taking any type of medications that might interfere with the bodyweight (steroids, diuretics, and thyroid hormones).	Delivery	Weight gain, eating healthy	RR: 0.48 [0.24, 0.95]
	Aşcı & Rathfisch (2016)	Pregnant women aged over 18, who had no health problems, did not intend to lose weight in pre‐pregnancy period, got pregnant in natural ways for two times at most, and were pregnant for a period of 3 months or less, were included in the study	Pregnancy complications	Delivery	Pregnancy weight gain, healthy eating	RR: 1.13 [0.64, 2.02]
	Lin et al. (2020)	Adult pregnant women aged 18 years or older who had at least one risk factor of GDM were included in this study. The risk factors of GDM were defined as follows: age ≥ 35 years, pre‐pregnancy body mass index (BMI) ≥ 25 kg/m^2^, family history of diabetes mellitus, history of PCOS, and history of GDM in a previous pregnancy.	Pregnant women with pre‐existing diabetes mellitus, multiple pregnancies, use of medication that influences glucose metabolism (e.g., steroids, b‐adrenergic agonists, and anti‐psychotic drugs), physical disability, or severe psychiatric disorders.	Delivery	Healthy eating, exercise	RR: 0.72 [0.54, 0.96]
	Liu et al. (2009)	1) healthy pregnant women; 2) at their third trimester; 3) had at least three routine examinations at these antenatal clinics.	1) over 35 years of age; 2) had pregnancy complications such as cardiovascular, digestive, endocrine and reproductive system diseases; 3) had multiple gestations; and 4) could not have a vaginal birth because of predisposing factors such as an abnormal pelvis, malposition, or uterine fibroids.	Delivery	Healthy eating, diversity	RR: 0.99 [0.79, 1.25]
	Nyamasege (2018)	Pregnant women were prospectively included throughout the trimesters, residents of two densely populated slums (Korogocho, and Viwandani) located 7 km apart from each other.	Women of reproductive age who were to deliver before the intervention started.	Delivery	Knowledge of pregnancy complications, importance of taking iron and folate supplements	RR: 1.04 [0.80, 1.35]

Abbreviations: ANC, antenatal care, GDM, gestational diabeties mellitus; GWG, gestational weight gain; IFA, iron‐folic acid; RR, risk ratio.

**Table 3:** Summary of studies that reported maternal behavior outcomes.

Paper	Maternal behavior outcomes of interest	Exclusion criteria	What trimester was maternal health measured? (i.e.)	What was the nutrition intervention? (i.e., counseling on dietary diversity, counseling on IFA)	What was the change from the intervention?
Aşcı & Rathfisch (2016)	Dietary intake	Pregnancy complications	Second trimester	Pregnancy weight gain, healthy eating	Mean difference of 199.00 calories [−43.15, 441.15] from control (standard ANC)
Jing et al. (2015)	Dietary intake	Pregnancy‐related complications or general medical disorders not associated with pregnancy	Second trimester	Pregnancy weight gain	Mean difference of 125.34 calories [26.67, 224.01] from control (standard ANC)
Liu et al. (2009)	Dietary intake	‐	6 weeks after delivery	Healthy eating, exercise, support	Mean difference of 51.27 calories [30.06, 72.48] from control (standard ANC)
Aşcı & Rathfisch (2016)	Macronutrient intake—Protein	Pregnancy complications	Third trimester	Pregnancy weight gain, healthy eating	Mean difference of 14.70 g [12.87, 16.53] from control (standard ANC)
Jing et al. (2015)	Macronutrient intake—Protein	Pregnancy‐related complications or general medical disorders not associated with pregnancy	Second trimester	Pregnancy weight gain	Mean difference of 5.91 g [2.37, 9.45] from control (standard ANC)
Aşcı & Rathfisch (2016)	Macronutrient intake—Fat	Pregnancy complications	Third trimester	Pregnancy weight gain, healthy eating	Mean difference of 7.00 g [−2.01, 16.01] from control (standard ANC)
Jing et al. (2015)	Macronutrient intake—Fat	Pregnancy‐related complications or general medical disorders not associated with pregnancy	Second trimester	Pregnancy weight gain	Mean difference of 2.73 g [−1.23, 6.69] from control (standard ANC)
Aşcı & Rathfisch (2016)	Macronutrient intake—carbohydrates	Pregnancy complications	Third trimester	Pregnancy weight gain, healthy eating	Mean difference of 18.50 g [−20.75, 57.75] from control (standard ANC)
Jing et al. (2015)	Macronutrient intake—carbohydrates	Pregnancy‐related complications or general medical disorders not associated with pregnancy	Second trimester	Pregnancy weight gain	Mean difference of 19.12 g [1.09, 37.41] from control (standard ANC)

Abbreviations: ANC, antenatal care; IFA, iron‐folic acid.

**Table 4:** Summary of studies that reported infant health outcomes of interest.

Infant health outcomes of interest	Paper	Exclusion criteria	What was the nutrition intervention? (i.e., counseling on Macronutrient intake, counseling on IFA)	What was the change from the intervention (Unit: effect size)
Stillbirths	Belizan et al. (1995)	With clinical evidence of cardiovascular, renal, or other chronic diseases; history of cerclage. Rh‐negative, or mental diseases	Nutrition during pregnancy, support during pregnancy	RR: 0.89 [0.55, 1.44]
	Kafatos et al. (1989)	Not reported	Nutrition during pregnancy	RR: 0.45 [0.11, 1.77]
	Kian et al. (2016)	Women who received diabetes training before entering the study.	Knowledge of pregnancy complications	RR: 0.33 [0.01, 7.96]
Preterm birth	Abdel‐Aziz et al. (2018)	younger than 18 years, have a history of previous abortion or stillbirth, presence of any chronic disease, and taking any type of medications that might interfere with the bodyweight (steroids, diuretics, and thyroid hormones).	Prevention of excessive GWG, healthy eating	RR: 0.84 [0.68, 1.04]
	Nyamasege (2018)	women of reproductive age who were to deliver before the intervention started	Knowledge of pregnancy complications, importance of taking iron and folate supplements	RR: 0.39 [0.17, 0.88]
Low birthweight	Belizan et al. (1995)	With clinical evidence of cardiovascular, renal, or other chronic diseases; history of cerclage. Rh‐negative, or mental diseases	Nutrition during pregnancy, support during pregnancy	RR: 0.92 [0.70, 1.21]
	Kafatos et al. (1989)	Not reported	Nutrition during pregnancy	RR: 1.14 [0.51, 2.56]
	Nyamasege (2018)	women of reproductive age who were to deliver before the intervention started	Knowledge of pregnancy complications, importance of taking iron and folate supplements	RR: 0.37 [0.20, 0.71]

Abbreviations: ANC, antenatal care, GWG, gestational weight gain; IFA, iron‐folic acid; RR, risk ratio.

##### Settings

Eight studies were conducted in Africa, two including African population from multiple countries (Nyamasege et al., 2021; Nyamasege et al., 2018), one in Ethiopia (Demilew et al., 2020), two in Malawi (Katenga‐Kaunda et al., 2021; Ziyenda Katenga‐Kaunda et al., 2020), one in Kenya (Isrctn, 2017), one in Benin (Villalon et al., 2010) and one in Egypt (Abdel‐Aziz et al., 2018). Twenty‐eight studies were conducted in Asia; 12 took place in Iran (Kiani Asiabar et al., 2018; Goodarzi‐Khoigani et al., 2018; Izadirad et al., 2017; Jahangiri et al., 2020; Kamalifard et al., 2012; Khoramabadi et al., 2015; Kian et al., 2016; Mazloomy‐Mahmoodabad et al., 2020; Mirmolaei et al., 2009; Mohsenzadeh‐Ledari et al., 2020; Saadatnia et al., 2021; Sharifirad et al., 2013), six in China (Jing et al., 2015; Lin et al., 2020; Liu et al., 2009; Liu et al., 2017; Tu et al., 2021; Zhou & Tang, 2020), three in India (Daniel et al., 2016; Garg & Kashyap, 2006; Sachdeva & Mann, 1994), two Turkey (Aşcı & Rathfisch, 2016; Kanber et al., 2011), two in Indonesia (Nahrisah et al., 2020; Permatasari et al., 2021), one in Malaysia (Soliman et al., 2019), one in Bangladesh (Jahan et al., 2014), on in Jordan (Abu‐Baker et al., 2021) and one in Nepal (Sunuwar et al., 2019).

Four studies were conducted in South America; two studies in Brazil (Malta et al., 2021; Vítolo et al., 2011), one study in Mexico (Perichart‐Perera et al., 2009), and one study included participants from several south American countries (Belizan et al., 1995).

One study was conducted in Greece at the time when it was classified as a LMIC (Kafatos et al., 1989).

##### Participants

All the studies included pregnant women from either low‐income, lower‐middle income or upper middle‐income countries. The population size ranged from 47 in Villalon et al. (2010) to 2230 in Belizan et al. (1995). Thirty‐eight studies described the participant characteristics by at least one element of PROGRESS‐+ (Abdel‐Aziz et al., 2018; Abu‐Baker et al., 2021; Kiani Asiabar et al., 2018; Aşcı & Rathfisch, 2016; Belizan et al., 1995; Daniel et al., 2016; Demilew et al., 2020; Garg & Kashyap, 2006; Goodarzi‐Khoigani et al., 2018; Hasneezah et al., 2020; Isrctn, 2017; Izadirad et al., 2017; Jahan et al., 2014; Jing et al., 2015; Kafatos et al., 1989; Kamalifard et al., 2012; Kanber et al., 2011; Katenga‐Kaunda et al., 2021; Khoramabadi et al., 2015; Kian et al., 2016; Lin et al., 2020; Liu et al., 2017; Malta et al., 2021; Mirmolaei et al., 2009; Mohsenzadeh‐Ledari et al., 2020; Nahrisah et al., 2020; Nyamasege et al., 2021; Nyamasege et al., 2018; Perichart‐Perera et al., 2009; Permatasari et al., 2021; Saadatnia et al., 2021; Sharifirad et al., 2013; Sunuwar et al., 2019; Tu et al., 2021; Villalon et al., 2010; Zhou & Tang, 2020; Ziyenda Katenga‐Kaunda et al., 2020). Thirty‐six studies (84%) reported the age of the pregnant women (Abu‐Baker et al., 2021; Kiani Asiabar et al., 2018; Aşcı & Rathfisch, 2016; Belizan et al., 1995; Daniel et al., 2016; Demilew et al., 2020; Garg & Kashyap, 2006; Goodarzi‐Khoigani et al., 2018; Hasneezah et al., 2020; Isrctn, 2017; Izadirad et al., 2017; Jahan et al., 2014; Jing et al., 2015; Kafatos et al., 1989; Kamalifard et al., 2012; Kanber et al., 2011; Katenga‐Kaunda et al., 2021; Khoramabadi et al., 2015; Kian et al., 2016; Lin et al., 2020; Liu et al., 2017; Malta et al., 2021; Mirmolaei et al., 2009; Mohsenzadeh‐Ledari et al., 2020; Nahrisah et al., 2020; Nyamasege et al., 2021; Nyamasege et al., 2018; Perichart‐Perera et al., 2009; Permatasari et al., 2021; Sharifirad et al., 2013; Sunuwar et al., 2019; Tu et al., 2021; Villalon et al., 2010; Zhou & Tang, 2020; Ziyenda Katenga‐Kaunda et al., 2020), four studies described the place of residence (Kanber et al., 2011; Katenga‐Kaunda et al., 2021; Liu et al., 2009; Villalon et al., 2010), eight described race or ethnicity of the participants (Belizan et al., 1995; Hasneezah et al., 2020; Isrctn, 2017; Malta et al., 2021; Nyamasege et al., 2021; Nyamasege et al., 2018; Permatasari et al., 2021; Villalon et al., 2010), 19 studies described occupation (Kiani Asiabar et al., 2018; Aşcı & Rathfisch, 2016; Belizan et al., 1995; Hasneezah et al., 2020; Isrctn, 2017; Izadirad et al., 2017; Kanber et al., 2011; Khoramabadi et al., 2015; Liu et al., 2017; Mirmolaei et al., 2009; Mohsenzadeh‐Ledari et al., 2020; Nahrisah et al., 2020; Nyamasege et al., 2021; Nyamasege et al., 2018; Permatasari et al., 2021; Saadatnia et al., 2021; Sharifirad et al., 2013; Sunuwar et al., 2019; Vítolo et al., 2011), two described gender roles (Katenga‐Kaunda et al., 2021; Permatasari et al., 2021), three reported religion (Nyamasege et al., 2021; Sunuwar et al., 2019; Villalon et al., 2010), 25 reported education level (Kiani Asiabar et al., 2018; Aşcı & Rathfisch, 2016; Belizan et al., 1995; Garg & Kashyap, 2006; Goodarzi‐Khoigani et al., 2018; Isrctn, 2017; Izadirad et al., 2017; Kamalifard et al., 2012; Kanber et al., 2011; Katenga‐Kaunda et al., 2021; Khoramabadi et al., 2015; Kian et al., 2016; Liu et al., 2017; Malta et al., 2021; Mirmolaei et al., 2009; Mohsenzadeh‐Ledari et al., 2020; Nahrisah et al., 2020; Nyamasege et al., 2021; Nyamasege et al., 2018; Permatasari et al., 2021; Saadatnia et al., 2021; Sharifirad et al., 2013; Sunuwar et al., 2019; Ziyenda Katenga‐Kaunda et al., 2020), and 23 reported socioeconomic status (Abdel‐Aziz et al., 2018; Abu‐Baker et al., 2021; Kiani Asiabar et al., 2018; Belizan et al., 1995; Garg & Kashyap, 2006; Goodarzi‐Khoigani et al., 2018; Hasneezah et al., 2020; Isrctn, 2017; Izadirad et al., 2017; Jahan et al., 2014; Kamalifard et al., 2012; Kanber et al., 2011; Katenga‐Kaunda et al., 2021; Kian et al., 2016; Lin et al., 2020; Liu et al., 2017; Malta et al., 2021; Mohsenzadeh‐Ledari et al., 2020; Nahrisah et al., 2020; Permatasari et al., 2021; Saadatnia et al., 2021; Ziyenda Katenga‐Kaunda et al., 2020), 10 social capital (Belizan et al., 1995; Isrctn, 2017; Kanber et al., 2011; Katenga‐Kaunda et al., 2021; Nahrisah et al., 2020; Nyamasege et al., 2021; Nyamasege et al., 2018; Villalon et al., 2010; Vítolo et al., 2011; Ziyenda Katenga‐Kaunda et al., 2020).

##### Interventions

Twenty five studies were conducted in a community setting such as neighborhoods and community centers (Abu‐Baker et al., 2021; Aşcı & Rathfisch, 2016; Demilew et al., 2020; Garg & Kashyap, 2006; Goodarzi‐Khoigani et al., 2018; Isrctn, 2017; Izadirad et al., 2017; Jahangiri et al., 2020; Kafatos et al., 1989; Kamalifard et al., 2012; Katenga‐Kaunda et al., 2021; Khoramabadi et al., 2015; Malta et al., 2021; Mazloomy‐Mahmoodabad et al., 2020; Mirmolaei et al., 2009; Nahrisah et al., 2020; Nyamasege et al., 2021; Nyamasege et al., 2018; Perichart‐Perera et al., 2009; Permatasari et al., 2021; Saadatnia et al., 2021; Sachdeva & Mann, 1994; Sharifirad et al., 2013; Vítolo et al., 2011; Ziyenda Katenga‐Kaunda et al., 2020), 11 were in hospitals (Kiani Asiabar et al., 2018; Belizan et al., 1995; Daniel et al., 2016; Jing et al., 2015; Kanber et al., 2011; Kian et al., 2016; Lin et al., 2020; Mohsenzadeh‐Ledari et al., 2020; Sunuwar et al., 2019; Tu et al., 2021; Zhou & Tang, 2020) and six were conducted in clinics (Abdel‐Aziz et al., 2018; Hasneezah et al., 2020; Jahan et al., 2014; Liu et al., 2009; Liu et al., 2017; Villalon et al., 2010).

The nature of the counseling was one to one sessions (patient with provider) in 11 studies (Kiani Asiabar et al., 2018; Belizan et al., 1995; Isrctn, 2017; Jahangiri et al., 2020; Kafatos et al., 1989; Kanber et al., 2011; Katenga‐Kaunda et al., 2021; Lin et al., 2020; Nyamasege et al., 2018; Vítolo et al., 2011; Zhou & Tang, 2020). Group sessions were used in eight studies (Izadirad et al., 2017; Jahan et al., 2014; Khoramabadi et al., 2015; Liu et al., 2009; Saadatnia et al., 2021; Sharifirad et al., 2013; Sunuwar et al., 2019; Villalon et al., 2010), while the remaining studies were mixtures of both approaches including written guidance documents.

Eight studies considered the local culture in the conduct of the nutrition counseling (Abu‐Baker et al., 2021; Belizan et al., 1995; Demilew et al., 2020; Katenga‐Kaunda et al., 2021; Nahrisah et al., 2020; Nyamasege et al., 2021; Permatasari et al., 2021; Villalon et al., 2010).

Classification of interventions by empowerment approach: Classification of the empowerment approach integrating nutrition counseling according to Riddle et al.'s framework shows that most (29 studies) of the counseling interventions used an unclear empowerment approach (Abdel‐Aziz et al., 2018; Abu‐Baker et al., 2021; Kiani Asiabar et al., 2018; Daniel et al., 2016; Demilew et al., 2020; Goodarzi‐Khoigani et al., 2018; Hasneezah et al., 2020; Izadirad et al., 2017; Jahan et al., 2014; Jahangiri et al., 2020; Kafatos et al., 1989; Kanber et al., 2011; Khoramabadi et al., 2015; Kian et al., 2016; Lin et al., 2020; Liu et al., 2009; Mazloomy‐Mahmoodabad et al., 2020; Mirmolaei et al., 2009; Mohsenzadeh‐Ledari et al., 2020; Nyamasege et al., 2018; Perichart‐Perera et al., 2009; Saadatnia et al., 2021; Sachdeva & Mann, 1994; Sharifirad et al., 2013; Sunuwar et al., 2019; Tu et al., 2021; Villalon et al., 2010; Vítolo et al., 2011; Zhou & Tang, 2020) and four studies implemented a complete empowerment model (Katenga‐Kaunda et al., 2021; Ziyenda Katenga‐Kaunda et al., 2020). The approach toward empowerment for the remaining studies was partial.

##### Comparison

All the studies compared two‐way interactive nutrition counseling to standard ANC. Only one study had multiple arms; face‐to‐face counseling, guidance booklet and routine ANC (Kian et al., 2016).

#### 5.1.3 Excluded studies

We excluded 95 studies in total (including  nine duplicates); 31 were conducted in high income setting; 17 studies were excluded because they did not follow EPOC study design recommendations (Akter et al., 2012; Caut et al., 2019; Della Libera et al., 2011; Gila‐Diaz et al., 2021; Jindal et al., 2021; Kamudoni et al., 2016; Nair et al., 2020; NCT04694235, 2021; Parra et al., 2005; Rifayanto et al., 2021; Santos et al., 2013; Selvakumar, 2015; Shimpuku et al., 2019; Thaver et al., 2020; Wijaya‐Erhardt et al., 2014); 14 studies targeted participants that were not relevant to this review (dos Santos et al., 2019; Fayasari et al., 2019; Fernald et al., 2016; Kimani‐Murage et al., 2013; Kimani‐Murage et al., 2015; Lin et al., 2017; Mwangi et al., 2020; Nguyen et al., 2017; Nguyen et al., 2021; Nugraheni et al., 2020; Owais et al., 2017; Scherbaum et al., 2017; Stewart et al., 2020); 10 did not have a nutrition counseling intervention for pregnant women (Abrha et al., 2020; Diddana et al., 2018; Fan et al., 2010; Ferrari et al., 2020; Gomes et al., 2019; Hajian et al., 2020; Pawalia et al., 2020; RBR‐5jy777, 2019; Sahran et al., 2021; Swaminathan, 2015; Nimbalkar et al., 2017, nutrition counseling was not interactive in eight studies (Abujilban et al., 2018; Adhikari al., 2009; Costa de Oliveira et al., 2018; Delfiani et al., 2021; Deveer al., 2013; Teweldemedhin et al., 2021; Javadi et al., 2020; Nimbalkar et al., 2017); four studies did not describe the nature of the nutrition counseling (RBR‐7yx36h, 2019; Saadatnia et al., 2021; Sirajuddin et al., 2018; TCTR20210317002, 2021) and two studies did not assess the effectiveness of counseling or its modalities (Mshanga et al., 2019; Sun et al., 2016).

### 5.2 Risk of bias in included studies

#### 5.2.1 RCTs

**Figure 3:**
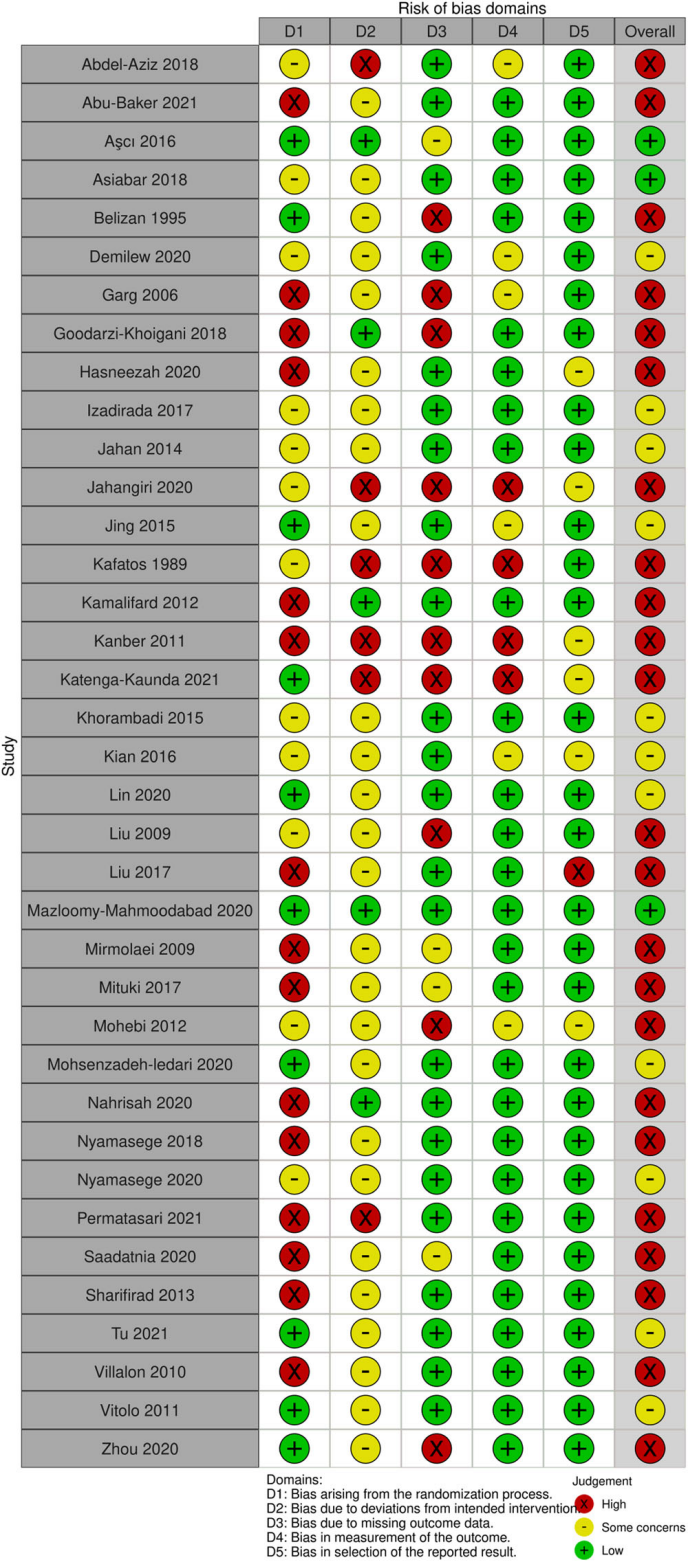
Risk of Bias summary: Review author's judgment for each study.

Figure 3 shows the ROB assessed using the Cochrane ROB 2.0 tool in the 36 RCTs that addressed nutrition counseling for pregnant women.

##### Bias arising from the randomization process

Fifteen trials were high ROB due to inadequate randomization and lack of information to judge the randomization process (Abu‐Baker et al., 2021; Garg & Kashyap, 2006; Goodarzi‐Khoigani et al., 2018; Hasneezah et al., 2020; Isrctn, 2017; Kamalifard et al., 2012; Kanber et al., 2011; Liu et al., 2017; Mirmolaei et al., 2009; Nahrisah et al., 2020; Nyamasege et al., 2018; Permatasari et al., 2021; Saadatnia et al., 2021; Sharifirad et al., 2013; Villalon et al., 2010). The randomization process was not clearly mentioned in eleven trials, resulting in some concerns with ROB (Abdel‐Aziz et al., 2018; Kiani Asiabar et al., 2018; Demilew et al., 2020; Izadirad et al., 2017; Jahan et al., 2014; Jahangiri et al., 2020; Kafatos et al., 1989; Khoramabadi et al., 2015; Kian et al., 2016; Liu et al., 2009; Nyamasege et al., 2021). The remaining trials had low ROB regarding randomization.

##### Bias due to deviations from intended intervention

Six trials were deemed high ROB with regard to blinding of participants/personnel (Abdel‐Aziz et al., 2018; Jahangiri et al., 2020; Kafatos et al., 1989; Kanber et al., 2011; Katenga‐Kaunda et al., 2021; Permatasari et al., 2021). Twenty‐six trials had insufficient information resulting in some concerns. The remaining trials had low ROB (Aşcı & Rathfisch, 2016; Goodarzi‐Khoigani et al., 2018; Mazloomy‐Mahmoodabad et al., 2020; Kamalifard et al., 2012; Nahrisah et al., 2020).

##### Bias due to missing outcome data

Imbalanced losses were found in four trials (Belizan et al., 1995; Garg & Kashyap, 2006; Liu et al., 2009; Zhou & Tang, 2020). The issue of incomplete outcome data was not addressed adequately in three trials resulting in some concerns in ROB (Isrctn, 2017; Mirmolaei et al., 2009; Saadatnia et al., 2021). The missing outcome data were balanced across trial groups in the rest of the studies.

##### Bias in measurement of the outcome

Almost all the studies were low ROB. Five studies had some concerns in the appropriateness of measuring the outcome (Demilew et al., 2020; Garg & Kashyap, 2006; Jing et al., 2015; Kian et al., 2016; Sharifirad et al., 2013).

##### Bias in selection of the reported result

All the relevant outcomes were reported in most of the trials. Two trials had some concerns about selective reporting (Kian et al., 2016; Hasneezah et al., 2020). One trial had evidence of selectively reporting outcomes (Liu et al., 2017).

#### 5.2.2 Nonrandomized controlled studies (NRS)

ROB assessment for NRS was conducted using the NOS tool. All the studies were truly or somewhat representative of the average pregnant women in the community, and the controls were drawn from the same cohorts. One study used self‐reporting to ascertain nutrition counseling (Sachdeva & Mann, 1994) compared to using structured interviews with blinded personnel. All studies demonstrated that the outcome of interest was not present at the start of the study. None of the studies controlled for gestational age when comparing groups in the design or analysis. Two studies assessed outcomes independently (Perichart‐Perera et al., 2009; Sunuwar et al., 2019). All the studies followed up with the participants until after delivery and accounted for all the subjects after following up.

### 5.3 Synthesis of results

Despite differences in intervention content and design, we agreed that the interventions were sufficiently similar to combine. All the included studies involved interactive counseling, and if support was available, it was provided in both arms. We tabulated the studies reporting maternal health outcomes, maternal behavior outcomes, and infant health outcomes in Tables 2, 3, 4. However, measures that could not be pooled due to incomplete reporting of results or differences in measurement approaches were described for each study separately while providing effect estimates and measures of variance.

#### 5.3.1 Primary outcomes

*Maternal*—*Mortality*. There were no studies that assessed this outcome.

**Figure 4:**
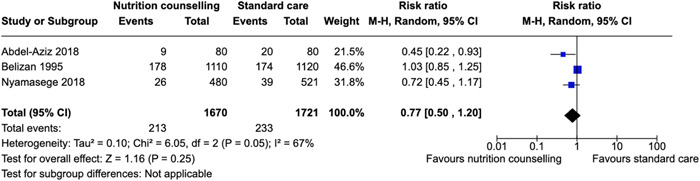
Forest plot of comparison: Nutrition counseling versus standard care, outcome: Anemia.

*Maternal*—*Anemia*. Three RCTs (Abdel‐Aziz et al., 2018; Belizan et al., 1995; Nyamasege et al., 2018), involving 3391 participants, between the first and second pregnancy term assessed the impact of nutrition counseling on anemia. Nutrition counseling may make little to no difference in the incidence of anemia in pregnant women (RR: 0.77, 95% CI, 0.50–1.20, three RCTs; *I* ^2^ = 67%) (Figure 4).

One NRS (Garg & Kashyap, 2006) using unadjusted raw number of events reported that nutrition counseling may reduce the risk of Anemia (RR: 0.71, 95% CI, 0.52–0.97, one NRS).

*Maternal*—*Iron deficiency*. None of the included studies reported maternal iron deficiency or assessed iron intake.

*Maternal*—*Intent to breastfeed*. Two RCTs reported the impact of nutrition counseling on the intent to breastfeed. Mituki et al. (Isrctn, 2017) used a breastfeeding efficacy tool that included whether a mother chooses to initiate breastfeeding. Participants in the counseling group probably increased their breastfeeding efficacy (difference of means = 2.87 [scale 1–5 where 1 is not at all confident], 95% CI = 1.76–3.98). Another study asked the participants about their intent to breastfeed according to the WHO definition (“infant receives only breastmilk from mother or wet nurse, or expressed milk, and no other liquids or solids except drops or syrups consisting of vitamins, mineral supplements or medicines”) (World Health Organization) and found little to no difference (RR: 1.01, 95% CI, 0.94–1.09, one RCT) (Nyamasege et al., 2021).

*Maternal*—*Timely initiation of breastfeeding*. Nutrition counseling probably leads to breastfeeding initiation immediately after birth (RR: 1.72, 95% CI, 1.42–2.09, one RCT) (Jahan et al., 2014).

*Maternal*—*Adherence to iron supplement consumption*. Four NRS reported this outcome. Garg & Kashyap (2006) reported the distribution of participants according to the number of iron supplement tablets they ingested, unadjusted for confounders. We transformed the data to find the average number of tablets the participants ingested in each group. We found that, on average, women in the intervention group ingested more (72 tablets in the intervention vs 16 tablets in the control group). Another NRS (Hasneezah et al., 2020) of 162 participants reported a generalized estimating equation (GEE) analysis of high compliance level toward supplementation adjusted for the study group, measuring time point and birth spacing, and found that nutrition counseling was effective in improving compliance (AOR = 4.59, 95% CI, 1.58–13.35). Similarly, Nahrisah et al. (2020) found a significant difference in the mean number of iron‐folic acid (IFA) intake adjusted for IFA intake before intervention, age, education, and monthly family income (difference of means = 52.30 tablets, 95% CI = 47.46, 57.14). Sachdeva et al. (1994) reported that all the participants in the nutrition counseling group took folifer tablets regularly, while only 37% of the subjects in the control group consumed their tablets.

*Maternal—Adherence to ANC*. Two RCTs reported this outcome. Belizan et al. (1995) found that interactive nutrition counseling had little to no effect on adherence to antenatal visits (difference in means = −0.10 visits, 95% CI = −0.42, 0.22). Similar findings were found in Nyamasege et al. (2018) with a mean of 4 visits for both groups. However, in this study, when looking at adherence to six or more ANC visits, nutrition counseling probably leads to improved adherence (RR: 1.83, 95% CI, 1.23–2.73, one RCT).

**Figure 5:**
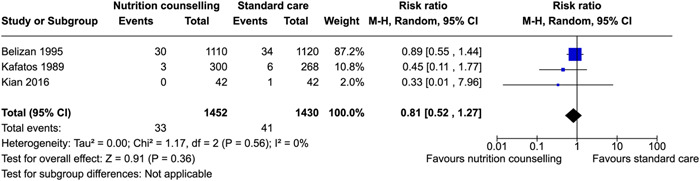
Forest plot of comparison: Nutrition counseling vs standard care, outcome: Stillbirths.

*Infant—Stillbirths*. Three RCTs reported on stillbirths. Nutrition counseling showed little to no effect on the risk of stillbirths (Belizan et al., 1995; Kian et al., 2016; Kafatos et al., 1989) (RR: 0.81, 95% CI, 0.52–1.27, three RCTs; *I* ^2^ = 0%) (Figure 5).

One NRS (Sachdeva et al., 1994) of 66 participants reported one stillbirth in the nutrition counseling group using unadjusted raw number of events.

**Figure 6:**
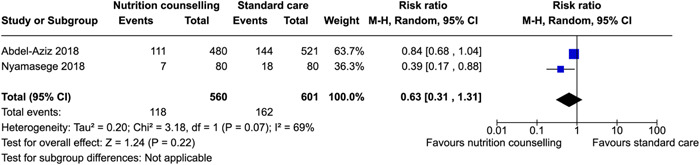
Forest plot of comparison: Nutrition counseling vs standard care, outcome: Preterm birth.

*infant*—*Preterm birth*. This outcome was reported in two RCTs (Abdel‐Aziz et al., 2018, and Nyamasege et al., 2018). Nutrition counseling may have little to no impact on the risk of preterm birth for neonates (RR: 0.63, 95% CI, 0.31–1.31, two RCTs; *I* ^2^ = 69%) (Figure 6).

One NRS (Perichart‐Perera et al., 2009) of 194 participants found a greater but non‐significant benefit in the nutrition counseling group (RR: 0.61, 95% CI, 0.28–1.33, one NRS).

*Infant*—*perinatal mortality*. Belizan et al. (1995) found little to no benefit for nutrition counseling on reducing perinatal mortality (RR: 0.89, 95% CI, 0.58–1.37, one RCT).

#### 5.3.2 Secondary outcomes

**Figure 7:**
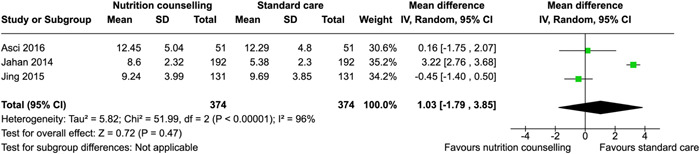
Forest plot of comparison: Nutrition counseling versus standard care, outcome: Gestational weight gain (kg).

*Maternal*—*Gestational weight gain*. Eight RCTs reported the impact of nutrition counseling on gestational weight gain using various methods. Four trials reported gestational weight gain as a continuous outcome. However, the findings were substantially heterogeneous. Aşcı & Rathfisch (2016) and Jing et al. (2015) found little to no difference between the nutrition counseling and the control groups, while Jahan et al. (2014) found that nutrition counseling significantly impacted gestational weight gain. (Figure 7). Tu et al. (2021) measured weight gain in the first 12 weeks of pregnancy and found no association with nutrition counseling (difference in means = −0.07 kg, 95% CI = −0.33, 0.19).

**Figure 8:**
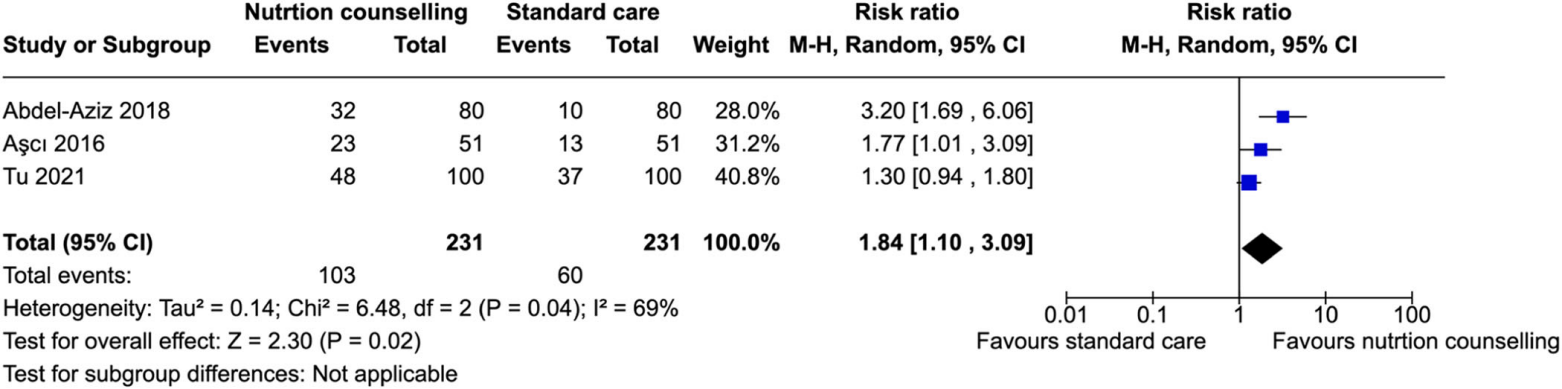
Forest plot of comparison: Nutrition counseling versus standard care, outcome: Gestational weight gain (within recommendations).

Four RCTs reported gestation weight gain as a dichotomous outcome; the proportion of women that gained weight during pregnancy within, below, and above standard health recommendations (Abdel‐Aziz et al., 2018; Aşcı & Rathfisch, 2016; Jing et al., 2015; Tu et al., 2021). Gestational weight gain within recommendations favored the nutrition counseling group (RR: 1.84 95% CI, 1.10–3.09, three RCTs; *I* ^2^ = 69%) (Figure 8). One study reported no difference in gestation weight gain above recommendations between nutrition counseling and control groups (Jing et al., 2015). Abdel‐Aziz et al. (2018) found that weight gain above or below recommendations was less likely to occur in participants that received nutrition counseling.

**Figure 9:**
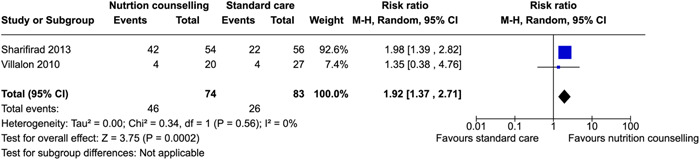
Forest plot of comparison: Nutrition counseling versus standard care, outcome: Gestational weight gain (within recommendations) using non‐randomized studies.

Five NRS reported the impact of nutrition counseling on gestational weight gain using unadjusted data; four measured it as a continuous outcome (Daniel et al., 2016; Liu et al., 2017; Villalon et al., 2010; Sunuwar et al., 2019) and two measured it as a dichotomous outcome (Sharifirad et al., 2013; Villalon et al., 2010). For the continuous measurement, there was substantial heterogeneity, with three studies showing greater benefit in the nutrition counseling group, and one study showing the opposite (Liu et al., 2017). Two NRS reported gestational weight gain as a dichotomous outcome. Nutrition counseling likely improved gestational weight gain within recommendations (RR: 1.92; 95% CI, 1.37–2.71, two NRS; *I* ^2^ = 0%) (Figure 9). Sharifirad et al. (2013) reported a lower risk of women that received nutrition counseling to gain weight more or less than recommended. None of the studies controlled for food security.

**Figure 10:**
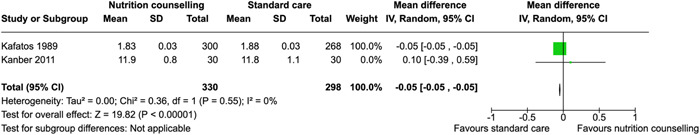
Forest plot of comparison: Nutrition counseling versus standard care, outcome: Hemoglobin concentration.

*Maternal*—*Hemoglobin concentration*. Two RCTs with a total of 628 participants reported a concentration of hemoglobin (Kafatos et al., 1989; Kanber et al., 2011). Nutrition counseling did not improve hemoglobin concentration compared to standard care (Mean difference: −0.05 mmol/L, 95% CI, −0.05 to −0.05, two RCTs; *I* ^2^ = 0%) (Figure 10).

**Figure 11:**
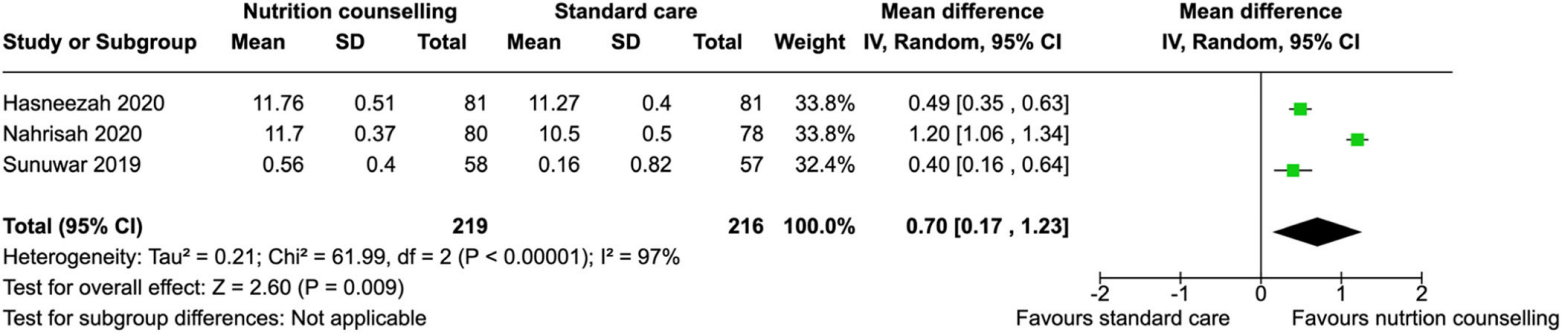
Forest plot of comparison: Nutrition counseling versus standard care, outcome: Hemoglobin concentration using nonrandomized studies.

Three NRS reported hemoglobin concentration without adjustment found contrasting results to the previously mentioned trials (Figure 11).

**Figure 12:**
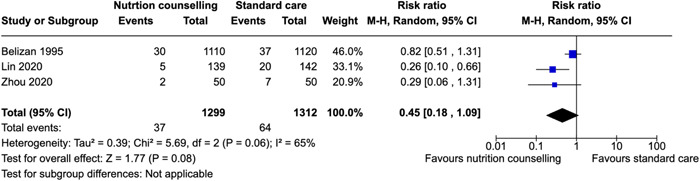
Forest plot of comparison: Nutrition counseling versus standard care, outcome: hemorrhage.

*Maternal*—*Hemorrhage*. Three RCTs including 2634 participants reported this outcome. Nutrition counseling reduced the risk of postpartum hemorrhage; however, it was not statistically significant (Belizan et al., 1995; Lin et al., 2020; Zhou & Tang, 2020) (RR: 0.45; 95% CI, 0.18–1.09, three RCTs; *I* ^2^ = 65%) (Figure 12).

**Figure 13:**
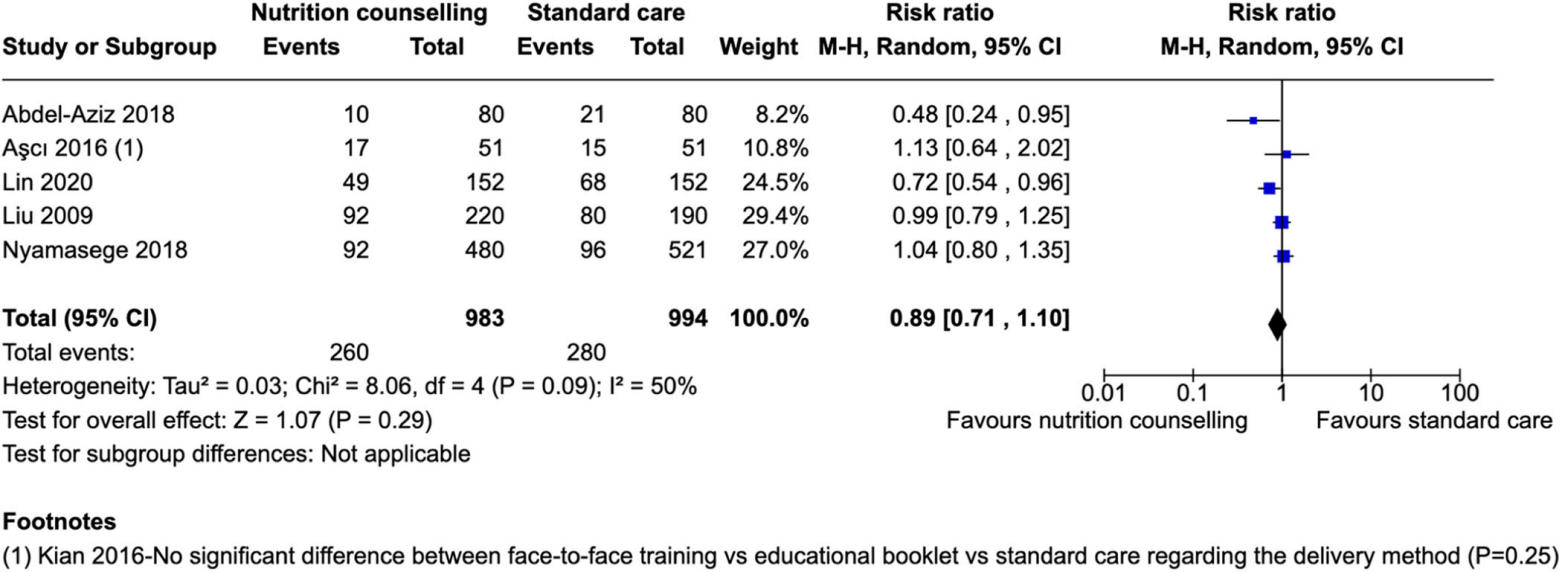
Forest plot of comparison: Nutrition counseling versus standard care, outcome: Cesarean section delivery.

*Maternal*—*Mode of delivery at birth*. Six trials reported the impact of nutrition counseling on cesarean delivery (Abdel‐Aziz et al., 2018; Aşcı & Rathfisch, 2016; Lin et al., 2020; Liu et al., 2009; Nyamasege et al., 2018; Kian et al., 2016). However, one trial only reported the *p* value for the statistical test between the groups (Kian et al., 2016). Pooled analysis of the remaining five RCTs shows that there is no difference between groups for cesarean delivery (RR: 0.89; 95% CI, 0.71–1.10, five RCTs; *I* ^2^ = 50%) (Figure 13).

One NRS (Liu et al., 2017) using unadjusted raw number of events also found no difference (RR: 0.99; 95% CI, 0.79–1.25, one NRS, 410).

**Figure 14:**
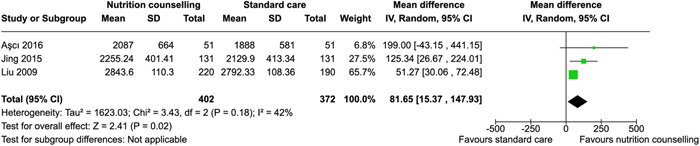
Forest plot of comparison: Nutrition counseling versus standard care, outcome: Dietary intake.

*Maternal*—*Dietary intake during pregnancy*. Four RCTs reported this outcome. Nutrition counseling encouraging caloric intake was associated with greater dietary caloric intake (Aşcı & Rathfisch, 2016; Jing et al., 2015; Liu et al., 2009) (difference in mean: 81.65 Calories, 95% CI, 15.37–147.93, three RCTs; *I* ^2^ = 42%) (Figure 14). Another trial reported that women receiving nutrition counseling had a lower caloric consumption versus those in the control group (Kafatos et al., 1989).

One NRS study (Sachdeva & Mann, 1994) also reported significant improvement in the caloric intake.

**Figure 15:**
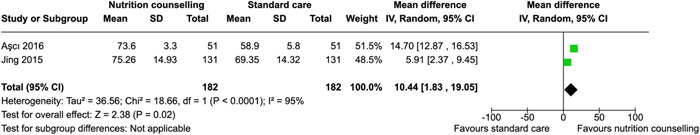
Forest plot of comparison: Nutrition counseling versus standard care, outcome: Macronutrtient intake—Protein.

**Figure 16:**
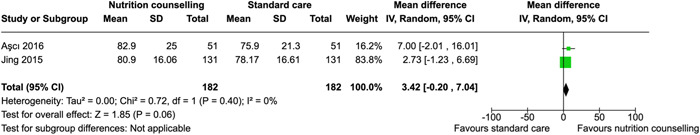
Forest plot of comparison: Nutrition counseling versus standard care, outcome: Macronutrtient intake—Fat.

**Figure 17:**
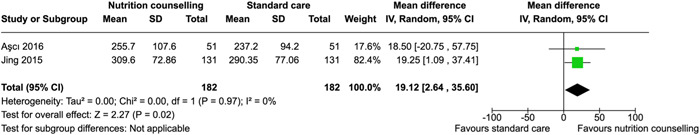
Forest plot of comparison: Nutrition counseling versus standard care, outcome: Macronutrient intake—Carbohydrates.

*Maternal*—***Macronutrient intake** during pregnancy*. The outcome was reported in three RCTs (Aşcı & Rathfisch, 2016; Demilew et al., 2020; Jing et al., 2015). Nutrition counseling may improve protein intake however there is considerable heterogeneity between studies (difference in mean: 10.44 g, 95% CI, 1.83–19.05, two RCTs; *I* ^2^ = 95%) (Figure 15). There was little to no difference for fat intake (difference in mean: 3.42 g, 95% CI, −0.20 to 7.04, two RCTs; *I* ^2^ = 0%) (Figure 16), but nutrition counseling improved carbohydrate intake (difference in mean: 19.12 g, 95% CI, 2.64–35.60, two RCTs; *I* ^2^ = 0%) (Figure 17). Another RCT (Demilew et al., 2020) reported significantly greater dietary diversity among women who received nutrition counseling.

**Figure 18:**
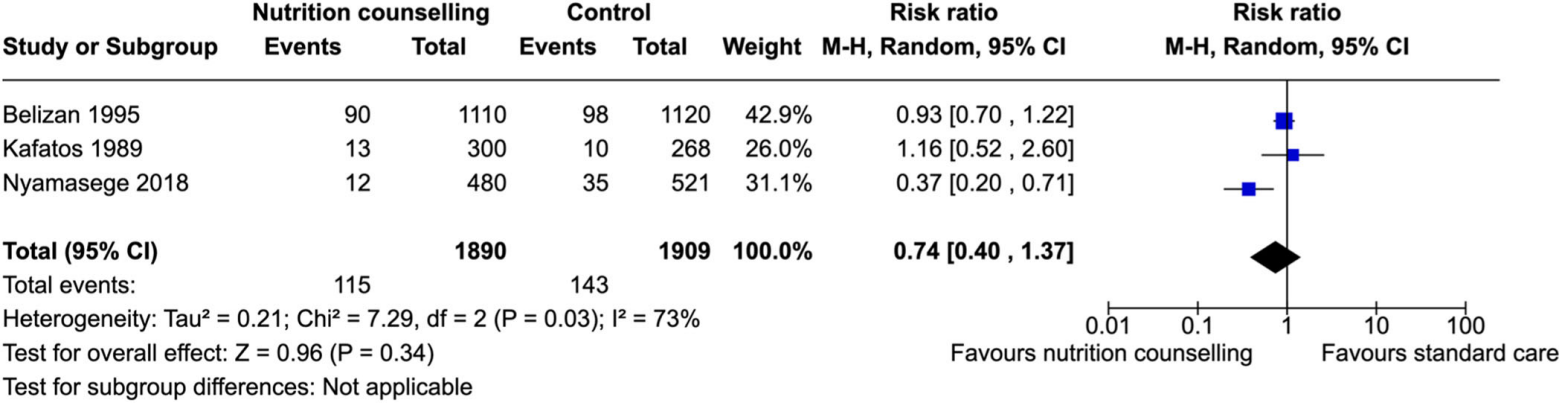
Forest plot of comparison: Nutrition counseling versus standard care, outcome: Low birthweight.

*Infant*—*Low birthweight*. Three RCTs reported this outcome (Belizan et al., 1995; (Kafatos et al., 1989; Nyamasege et al., 2018). Participants in the nutrition counseling group had little to no difference in the risk of giving birth to neonates with low birthweight (RR: 0.74, 95% CI, 0.40–1.37, three RCTs; *I* ^2^ = 73%) (Figure 18).

One NRS study (Perichart‐Perera et al., 2009) reported a nonsignificant difference in the proportion of newborns born with low birthweight (*p* = 0.45) using unadjusted raw number of events.

*Infant*—*Small for gestational age*. Nutrition counseling may not reduce the risk of small for gestational age in neonates (Kafatos et al., 1989) (RR: 1.01, 95% CI, 0.52–1.99, one RCT).

##### Effects of the intervention across PROGRESS‐+

One RCT (Liu et al., 2009) reported the impact of nutrition counseling on mode of delivery disaggregated by place of residence; 142 participants in urban and 160 in rural. In urban communities, women that received nutrition counseling had a lower risk of delivering their neonates via cesarean section (RR: 0.86, 95% CI, 0.64–1.14, one RCT). In contrast, women in rural communities had a higher risk (RR: 1.39, 95% CI, 1.05–1.83, one RCT). One RCT (Liu et al., 2017) reported that there was no significant difference in gestational weight gain (42‐day postpartum weight retention) between nutrition counseling and standard care groups (*p* > 0.05) or (*p* > 0.05) by education level or income level.

##### Subgroup analysis

We had too few studies to conduct subgroup analyses across intervention features (frequency/dose or timing of initiation of the intervention) and empowerment approaches.

##### Sensitivity analysis

We conducted sensitivity analysis using per‐protocol analysis and we did not observe any significant change in the strength of the associations for our outcomes of interest or their direction. This is reported in Table 5.

**Table 5:** Sensitivity analysis using per protocol analysis.

Outcome	*N* RCTs	ITT (risk ratio M‐H, random, 95% CI)	Per protocol (risk ratio M‐H, random, 95% CI)
Anemia	3	0.77 [0.50, 1.20]	0.76 [0.48, 1.20]
Gestational weight gain within recommendations	3	1.84 [1.10, 3.09]	1.81 [1.10, 2.98]
C‐section delivery	5	0.89 [0.71, 1.10]	0.91 [0.71, 1.16]
Hemorrhage	3	0.44 [0.18, 1.10]	0.45 [0.18, 1.09]
Stillbirths	3	0.81 [0.52, 1.27]	0.81 [0.51, 1.27]
Preterm births	2	0.63 [0.31, 1.31]	0.62 [0.29, 1.33]
Low birthweight	3	0.74 [0.40, 1.37]	0.74 [0.40, 1.36]

Abbreviations: CI, confidence interval; ITT, intention to treat; RCT, randomized controlled trial.

When we restricted our analyses to the studies with low ROB for the following domains: bias arising from the randomization process, bias due to deviations from intended intervention and bias due to missing outcome data, no significant change was observed in the direction or size of gestational weight gain, dietary intake, and mode of delivery.

## 6 DISCUSSION

### 6.1 Summary of main results

We included 52 studies, including pregnant women from LMICs, that evaluated the impact of two‐way interactive nutrition counseling. This review summarizes the current evidence on the effect of two‐way interactive nutrition counseling during pregnancy on maternal and infant outcomes.

Nutrition counseling may increase dietary caloric intake by 81.65 calories, protein (10.44 g) and carbohydrate (19.12 g) intake, and gestational weight gain within recommendations by 84%. Nutrition counseling may increase the probability of initiation of breastfeeding immediately after birth by 72% and reduce the risk of postpartum hemorrhage by 55%. Nutrition counseling had little to no effect on reducing anemia, stillbirths, preterm birth, small for gestational age, cesarian section delivery, improving hemorrhage or adherence to ANC. Sensitivity analysis was conducted for all outcomes using pre‐protocol analysis and studies with low ROB arising from the randomization process, bias due to deviations from intended intervention and bias due to missing outcome data, and no significant change in results. Studies on counseling failed to report on maternal mortality or iron deficiency. Women living in rural settings may have a higher risk of cesarean section delivery. Income or education level were not associated with gestational weight gain.

### 6.2 Overall completeness and applicability of evidence

We extensively searched multiple databases and gray literature sources to identify relevant papers. We also searched the reference list and citations of the included studies and contacted authors for missing data and studies inaccessible through our search engines. Data extraction and quality assessment were conducted in duplicate. Discrepancies were resolved by discussion or a third reviewer. We used the GRADE approach to assess the certainty of evidence.

This review summarizes the findings from 52 studies conducted in LMICs, particularly in Asia and Africa. Nutrition counseling may be effective in improving dietary caloric intake, protein, carbohydrate intake, and gestational weight gain (GWG) within health recommendations. Sensitivity analysis on the ROB domains did not significantly change the estimates. Of note, all the nutrition counseling practices were compared with standard ANC; however, practices of standard ANC vary globally in terms of content, methods of delivery and frequency, limiting the conclusions that can be drawn regarding the effects of two‐way interactive nutrition counseling. Moreover, the analyses were conducted on pregnant women living in LMICs, thus, they may not be generalizable to pregnant women living in high income countries. Even within LMICs, we found that most of the studies were skewed toward upper‐middle income countries such as Iran and China. Research in the very poor lower income countries where the need might be greatest was lacking. For example, only Ethiopia (one study found) and Malawi (two studies found) were represented. In the lower‐middle income classification Kenya (one study), India (three studies), Bangladesh (one study), Benin (one study), and Nepal (one study) were the only countries represented. This shows a large gap across LMICs on the availability of evidence informing the efficacy of two‐way nutrition counseling to improve maternal health outcomes. However, this is not surprising. Around two‐thirds of the population in these countries are in abject poverty and have pressing issues on education, health, and agriculture sector. Thus, they have limited expenditure in the areas of scientific research and education. Similar to research conducted in HICs, context should be considered when applying interventions to different countries and adapted accordingly.

### 6.3 Quality of the evidence

We judged the quality of the individual studies by utilizing the ROB 2.0 assessment tool for RCTs and cRCTs, and NOS for NRS. GRADE methodology was used to assess the quality of RCT evidence. The quality of evidence regarding the effects of nutrition counseling ranged from moderate to very low, largely due to heterogeneity, a common factor across all the studies included in this review. Less than half of the studies were randomized trials. Furthermore, across all the studies, there were concerns relating to different types of bias. Baseline characteristics and outcome measures were usually similar within studies. Participant's knowledge of allocated interventions was mostly unclear and most of the studies did not address the issue of incomplete outcome data. Similarly, efforts to minimize contamination bias were not adequately described. Almost all the pooled results were heterogenous, and the summary estimates were imprecise with wide CIs. We were unable to investigate subgroup analyses due to a lack of studies.

The NRS were of high quality with studies including representative samples of pregnant women, and authors demonstrating that the outcome of interest was not present at the start of the study. All the studies accounted for the study participants until after delivery. However, we were unable to test for publication bias for any of our outcomes due to the insufficient number of studies. We conducted a sensitivity analysis for outcomes with high ROB arising from the randomization process, bias due to deviations from intended intervention and bias due to missing outcome data, however, there was no significant change in effect estimates.

### 6.4 Potential biases in the review process

This review was conducted using the standard methods of Campbell and Cochrane collaborations. Two reviewers screened the studies for inclusion and abstracted the data from the included studies. Our eligibility criteria were decided a priori to the conduct of the review and published in a peer‐reviewed protocol detailing the methods used to conduct this review.

We included studies according to the EPOC study design criteria to ensure that we have included all relevant studies that could address the questions of interest. We also supplemented our database search with a gray literature search of international resources such as the WHO e‐Library and International Food policy Research Institute websites. However, the number of studies reporting the outcomes of interest was limited, impeding our ability to investigate subgroup effects and obtain precise estimates.

Judgments may have been made regarding whether nutrition counseling was two‐way interactive or didactic, as the definition of nutrition counseling was rarely explicitly reported. Thus, it is possible that different review teams may have reached different decisions regarding assessment of eligibility and ROB. Nonetheless, this review was conducted in collaboration with nutritionists, program managers and systematic review methodologists to minimize uninformed decisions.

### 6.5 Agreements and disagreements with other studies or reviews

Multiple systematic reviews have suggested that nutritional counseling carries the potential to positively influence infant morbidity and mortality. Despite none of our eligible studies measuring mortality, we still believe that it is a relevant outcome to be assessed when investigating the effectiveness of nutrition counseling. Given that most of the studies were conducted in community centers, we acknowledge that it would be unfeasible to measure such outcomes. Thus, large population studies are required to evaluate long‐term outcomes such as mortality, A Cochrane review found that providing women with education or counseling regarding their nutrition was associated with a reduced risk of preterm birth and low birth weight but found no effect on stillbirths and infant mortality (Ota et al., 2015). Similar to the review conducted by Ota et al., we found no effect on stillbirths, but in contrast, we did not find an effect on preterm birth. This may be because one of the studies included in preterm analysis conducted by Ota and colleagues was conducted outside of LMICs and could have shifted the effect estimate. Another meta‐analysis found that nutrition counseling improved maternal weight gain and reduced the incidence of maternal anemia (Girard & Olude, 2012). We also found an effect on maternal weight gain but not maternal anemia. Girard et al allowed any type of nutrition education that focused on improving maternal diet and nutritional status, no matter the method of education; this allowed more studies to be assessed (11 studies that reported anemia, three not from LMICs) compared to our review (three studies). It is noteworthy that these reviews examined nutritional counseling regardless of the setting in which the intervention was delivered. Thus, the contextual factors of LMIC settings may very well be the reason behind the attenuated effect estimates of our included studies. One facet of this phenomenon lies in the challenges of implementing interventions in LMIC. Similar to mental health care (Hanlon et al., 2014), screening (Anderson et al., 2008), and vaccination interventions (Ekwunife et al., 2017), implementing a nutrition counseling program among pregnant women might carry feasibility concerns due to a lack of resources or infrastructure. Indeed, one of our RCTs highlighted the logistical challenges nurses faced to reach the villages in which the program was set to be delivered during the winter months. Therefore, future research should focus on examining and addressing the implementation challenges surrounding the delivery of nutrition counseling. Although some studies found improved outcomes depending on the frequency of interactions or the intervention dose, we could not test this assumption due to insufficient papers with individual outcomes of interest.

We were limited in our ability to understand the impact of women's empowerment on nutrition counseling due to the limited number of studies available to inform the analysis. We included as many relevant empowerment indicators as possible to ensure that we captured any relevant studies. However, the number of studies that involved a full empowerment approach were scarce. Similarly, only one trial reported equity analyses. There is an urgent need for better data on women's empowerment and equity to facilitate research on the gender pathways to improve women's health. This includes transparent and thorough descriptions of the intervention by the authors and adopting an equity lens in the conduct and reporting of the studies to further the understanding of the mechanisms by which two‐way nutrition counseling impacts health outcomes.

## 7 AUTHORS’ CONCLUSIONS

### 7.1 Implications for practice and policy

#### 7.1.1 Standardized definition of nutrition counseling

The trials included in this review lacked a clear and consistent definition of nutrition counseling, what it entailed, and who it was delivered by (i.e., trained health care provider, community volunteer). It was, therefore, difficult to ascertain whether the “intervention” was consistent across studies and determine if the intervention was designed so that one could expect a change in the outcome(s) of interest. As such, a cautious interpretation of these findings and what they mean in practice is needed. A standard globally adopted definition of quality nutrition counseling considers the provider‐client relationship and emphasizes care that is a collaborative interaction where women have autonomy over their health, and is needed, particularly for LMICs.

#### 7.1.2 Nutrition counseling appears to influence nutrition behaviors, but more evidence needed to understand its impact on outcomes

Despite the lack of clarity on a definition of counseling and other weaknesses in and across the included studies, a positive association between counseling and uptake of nutrition recommendations (adherence to maternal micronutrient supplementation, increased caloric intake, and timely initiation of breastfeeding) was still found in this review. However, this positive association did not extend to health outcomes, such as a reduction in the risk of anemia. Anemia is a complex condition with varying etiologies. Effectively measuring counseling's potential impact on anemia prevention would require the included studies to control for the many causes of anemia (which they did not). Furthermore, the intervention should be provided for an extended period.

We know that pregnant women are particularly at risk of anemia caused by iron deficiency because their daily iron requirement drastically increases during pregnancy, and iron‐rich foods are often expensive, unavailable, or may fall outside plant‐based food practices. Plant‐based sources of iron are in a form (nonheme) that is difficult for the body to absorb in adequate quantities. Even in high‐income settings and settings where animal‐source foods are plentiful, it can be difficult for pregnant women to get all the iron they need each day from food alone. Due to this increased, difficult‐to‐meet need, and grave consequences of iron‐deficiency anemia during pregnancy, the WHO recommends daily iron supplementation during pregnancy beginning as early as possible and throughout pregnancy in all settings, with a dose composition of 30–60 mg of elemental iron to prevent anemia (World Health Organization, 2016). Iron supplementation in pregnancy is an evidence‐based intervention shown to reduce the risk of anemia at term by 70% (Peña‐Rosas et al., 2015), and this review confirms that counseling can improve adherence to supplementation. Furthermore, the studies did not indicate the anemia status of the women when entering pregnancy, and protocols for treatment of existing anemia differ from recommendations for prevention.

The implication for practice is that quality counseling on maternal micronutrient supplementation for reducing the risk of anemia and its consequences should be prioritized as part of comprehensive maternal nutritional care.

#### 7.1.3 Need for guidance on how to implement and measure quality nutrition counseling in LMICs

Lastly, once/even if a comprehensive, standardized, and global definition of quality nutrition counseling is adopted and effective measurement tools and processes are developed, and operationalized, practical guidance around how to implement quality nutrition counseling in LMIC settings remains a huge gap. Particularly for over‐burdened, under‐resourced frontline health workers in many LMIC contexts. Further implementation research is required to help identify key systems‐strengthening and capacity‐building entry points, tools, and techniques to support frontline health workers in systematically delivering quality, evidence‐based nutrition counseling as part of their ANC service. Involving professional dietitians in this process would help produce high‐quality programs that meet the women's needs.

### 7.2 Implications for research

Our review found improvements in maternal behavioral and health outcomes through interactive nutrition counseling during pregnancy. However, we also found very limited and highly varied definitions of interactive nutrition counseling, making it difficult to determine the most effective aspects of counseling. More methodologically rigorous trials focus on a clear selection of outcomes driven by the theory of change of nutrition counseling and how this could lead to improved maternal and infant behavioral and health outcomes. These studies should clearly define the intervention, what is being considered as nutrition counseling, what empowerment models are applied, descriptors and relevant status of the recipients, surrounding enablers and barriers, frequency and type of contacts, duration of each contact as well as the entire intervention, skills, and training of the providers, nutrition topics covered, key outcomes along the logic model that are collected, as well as other factors that could contribute to change in selected outcomes (e.g., prevalence of malaria as one contributor to anemia).

We recommend that future studies aim to test and evaluate the assumptions held in the logic model and create a validated theory of change of how nutrition counseling improves maternal and infant behavioral and health outcomes, as follows:

1. Validate the key components and linkages of the theory of change, starting with a standardized definition of nutrition counseling.
2. In terms of achieving outcomes, test and measure which aspects of counseling are most critical, such as soft skills of counseling, setting for counseling, time used for counseling, content relative to needs of the individuals and context, content of training, and supportive supervision provided to the health system personnel. The empowerment model is a critical component of counseling that must be clarified.
3. Employ qualitative methods in measuring the nutrition counseling process, which would assist researchers in understanding the relationship with behavioral outcomes, and to better interpret the results derived from quantitative analysis.
4. Test hypotheses along the impact pathway with adequately powered studies while considering the impact on populations at risk of experiencing inequities.
5. Hypothesize and test the causal inferences to evaluate the effectiveness of this complex intervention, nutrition counseling, on intermediate and long‐term outcomes, such as health outcomes, women's empowerment, and well‐being.
6. Seek to improve data on women's empowerment and equity to facilitate research on the gendered‐sensitive pathways to improve women's health. This includes developing and sharing transparent and thorough descriptions of the intervention by the authors and adopting an equity lens in the conduct and reporting of the studies to further the understanding of the mechanisms by which two‐way nutrition counseling impact health outcomes.

## CONTRIBUTIONS OF AUTHORS

Omar Dewidar, Ammar Saad and Aqeel Baqar ran the searches and performed the initial searching. Omar Dewidar, Jessica John, Mohamed Tarek Madani, Ammar Saad and Aqeel Baqar conducted the data extraction, quality assessment of all included studies. Omar Dewidar conducted the analyses. Omar Dewidar drafted the review. The remaining authors provided edits and suggestions to the review. Vivian Welch supervised the project.

## DECLARATIONS OF INTEREST

The authors declare to have no competing interests.

JBH, CJ, JKK, MKR, SR, SW are employees of Nutrition International (NI), which provided funding to the implementation of the study. However, funding was provided after the study was conceptualized and the proposal was drafted.

## PRELIMINARY TIMEFRAME

[This will be a heading at the same level as declarations of interest at publication stage.]

Approximate date for submission of the systematic review.

Please note this should be no longer than 2 years after protocol approval. If the review is not submitted by then, the review area may be opened up for other authors.

## PLANS FOR UPDATING THIS REVIEW

Once the review is completed, it will be updated every 5 years.

The Senior author will be responsible for updating the review.

## DIFFERENCES BETWEEN PROTOCOL AND REVIEW

There were no differences between protocol and review.

## PUBLISHED NOTES

**Characteristics of studies**

**Characteristics of included studies**

Aşcı 2016

**Table:** 

**Methods**	Individual Randomized Controlled Trial in family health center in Istanbul, Turkey
**Participants**	**Inclusion criteria:** Pregnant women aged over 18, who had no health problems, did not intend to lose weight in pre‐pregnancy period, got pregnant in natural ways for two times at most, and were pregnant for a period of 3 months or less, were included in the study. **Exclusion criteria:** Six people from intervention and control groups were excluded from the study due to reasons such as not coming to regular follow‐ups and pregnancy complications. **Age [Mean (SD)]: ‐ Intervention, Control:** 24.31 (4.22), 24.28 (4.15) **Working status [*n* (%)]:‐ Intervention, Control:** 7 (15.6), 2 (4.4) **Education mean (years): ‐ Intervention, Control:** 6.6 (2.8), 7.6 (3.2) **Income [*n* (%)]:‐ Intervention, Control:** Low: 15 (33.3), 11 (24.4) Middle: 30 (66.7), 34 (75.6) High: 0, 0
**Interventions**	**Intervention**—**Lifestyle intervention (*n* = 51)** **Description:** Four meetings were held with women regarding healthy lifestyle, nutrition, exercise, and weight follow‐up. At every meeting, objectives of nutrition and physical activity for optimal GWG were specified until the next meeting. Women reaching their objectives were praised and encouraged. Nutrition and physical activity levels of women who could not reach their objectives were reviewed with women, and a more intensive consultancy (repetition of basic nutrition and physical activity recommendations, reviewing individual objectives, and supportive phone consultancy) was provided. Interviews made in weeks 12–15, 16–18, and 20–24, a 15‐min health training prepared in the computer was carried out and then brochures were delivered. Each of these interviews lasted for about 1 h. In gestational weeks 12–15, the women were interviewed regarding the importance of healthy life and health practices. In gestational weeks 16–18, inter‐views were held concerning physical activity and exercises. Low‐level aerobic exercises recommended for pregnancy were shown and performed. Women were recommended to do mild‐moderate safe exercise types, which increase the heart rate to maximum 140 beats/min while being easily able to talk, for 30 min every other day (elliptical trainer, swimming, pilates, yoga, golf adapted for pregnancy, and mild level aerobic exercises), and maintain a more active lifestyle (taking walks every day, going to work by walking, using stairs instead of elevators, participating in sportive activities in their leisure times). In gestational weeks 20–24, interviews regarding nutrition were held. Women were informed about the basic nutrition principles (eating small but frequent meals at least 5 times a day, having breakfast every day, portions and amounts required to be consumed from all food groups, lessening consumption of sweet foods to once a day or less, increasing fibrin intake from bread, and decreasing fat in diet). Recommendations for consuming more healthy foods (e.g., fruit) instead of foods containing intensive energy (e.g., fast food and sweets) without any energy limitation according to the personal dietary habits were given. In week 37, only weights were followed up. **Nutrition counseling messaging:** Pender‚ health promotion model was used to allow women to express their experiences and opinions through open‐ended questions, e.g., what problems (barriers) you may have to eat healthier foods (more vegetables, more fruits, lower‐fat foods, and healthy grains), regarding nutrition and physical activity. Therefore, counseling, and behavioral skill‐building interventions were personalized according to the barriers for the individuals to displaying the behavior and their self‐efficacies in terms of performing the behavior. Women were informed about the importance of gaining weight within the recommended range and maintaining a healthy life. **Intervention adapted to local context:** Not reported **Women empowerment approach:** Partial empowerment model **Control (*n* = 51)** **Description:** Women are generally followed up by at least four times by midwives or nurses in standard care. In every follow‐up, weights of women are measured; however, they are not informed on what the GWG range appropriate for their BMI is and their personal weight changes.
**Outcomes**	Gestational weight gain, mode of delivery, Dietary intake during pregnancy, **Macronutrient intake** during pregnancy,
**Notes**	Funding: This study did not receive grant from any institution or organization.

Risk of bias

**Table:** 

Bias	Author's judgment	Support for judgment
Risk of bias arising from the randomization process	Low risk	The women were divided into randomized groups by a staff who was not involved in this study, by drawing lots (control *n* = 51, intervention = 51).
Risk of bias due to deviations from the intended interventions (effect of assignment to intervention)	Low risk	Participants were blind about which group they were involved in and the evaluated study outcomes. Groups were interviewed at different times and in different rooms in the center to control the interaction between groups.
Risk of bias due to deviations from the intended interventions (effect of adhering to intervention)	Low risk	Participants were blinded and carers were possibly not aware of the two groups. No information on failure to implement the intervention or issues of adherence.
Missing outcome data	Some concerns	Two participants discontinued in the intervention group, but not in the control group
Risk of bias in measurement of the outcome	Low risk	Outcomes measured using calibrated instruments, measured twice and mean was calculated.
Risk of bias in selection of the reported result	Low risk	All outcomes measured as stated in methods with defined tools.

Abdel‐Aziz 2018

**Table:** 

**Methods**	Individual Randomized Controlled Trial of pregnant women in ANC clinic, Center for Social and Preventive Medicine (CSPM), Pediatrics Hospital, Cairo University, Cairo Egypt
**Participants**	**Inclusion:** Primigravidae aged between 20 and 30 years in the first trimester (<12 weeks of gestation) of selected ANC clinic, free from history of any chronic medical problems were recruited to participate. **Exclusion:** Women were not eligible for participation if they were younger than 18 years (to avoid natural linear growth), having a history of previous abortion or stillbirth, presence of any chronic dis‐ease, and taking any type of medications that might interfere with the bodyweight (steroids, diuretics, and thyroid hormones). **Social Score [*n* (%)]—Intervention, Control:** Low<8: 1(1.2%), 2(2.5%) Intermediate (8–18): 74(92.5%), 74(92.5%) High (19–28): 5(6.3%), 4(5%)
**Interventions**	**Intervention—Dietary counseling (*n* = 80)** **Description:** The nutrition counselor who was a member of the research team discussed with the participants how to control weight gain during pregnancy and how to maintain or optimize a healthy lifestyle in a period of physical and mental changes. Each counseling session took about 20 min, except for the first session, which took approximately half an hour; the counselor explained the aim of the study and the intervention again. Participants of both groups received information on the recommendations of IOM7 for total GWG. **Nutrition counseling messaging:** Dietary counseling focused on the main topics considered important in the prevention of EGWG. The following dietary objectives were set for each participant to achieve or to maintain her body weight, having regular meal patterns based on the dietary guidelines for healthy pregnant women with considerations on the importance of dietary elements taken during pregnancy. Aiming to improve dietary practices, on five food groups of the food guide pyramid. **Intervention adapted to local context:** Not reported **Women empowerment approach:** Unclear **Control (*n* = 80)** **Description:** Standard care during routine ANC
**Outcomes**	Anemia, Gestational weight gain, Mode of delivery, Preterm Birth
**Notes**	Funding: None declared.

Risk of bias

**Table:** 

Bias	Author's judgment	Support for judgment
Risk of bias arising from the randomization process	Some concerns	Randomized allocation but no information on allocation concealment. There was no significant differences at baseline.
Risk of bias due to deviations from the intended interventions (effect of assignment to intervention)	Some concerns	No information on blinding of participants or personnel.
Risk of bias due to deviations from the intended interventions (effect of adhering to intervention)	High risk	“A considerable number of participants were illiterate, educational materials were taught, and a copy of written materials was provided to illiterate participants, who were advised to get help at home by their educated spouse. Unfortunately, dropouts at the end of the study endorse difficulty in follow‐up of the studied participants throughout the pregnancy until labor.”
Missing outcome data	Low risk	Data was reported for most participants.
Risk of bias in measurement of the outcome	Unclear risk	No information provided.
Risk of bias in selection of the reported result	Low risk	All outcomes measured as stated in methods with defined tools.

Abu‐Baker 2021

**Table:** 

**Methods**	Non‐randomized controlled study of pregnant women receiving antenatal care in public health center in Amman, Jordan
**Participants**	**Inclusion criteria:** Jordanian pregnant women, aged 18–40 years, and in the first or second trimesters. **Exclusion criteria:** Pregnant women with any medical problems or chronic diseases during pregnancy such as gestational diabetes, pre‐eclampsia, hypertension, or coronary heart disease to exclude the effect of other factors on the study outcomes. **Age [Mean (SD)]:** 28.38 (6.18) **Monthly family income [Mean (SD)]—Intervention, Control:** 490.32 (277.31), 451.60 (235.16) **Parity [Mean (SD)]—Intervention, Control:** 2.66 (2.04), 2.37 (2.23)
**Interventions**	**Intervention—Health education (*n* = 108)** **Description:** pretest structured questionnaires were completed face‐to‐face before the conduction of onsite educational sessions on six occasions and the posttest only was collected using phone calls after 6 weeks. It took 15–20 min to complete the questionnaires. At each visit, the nurse researcher gathered the assigned subgroup of 15–18 pregnant women in the meeting room where pretest structured questionnaires were completed. Then, one educational lecture was delivered for that group. At the end of the lecture, brochures were distributed to support the explained lecture. The researcher used WhatsApp application after that to communicate added information about dietary knowledge and practices to women at home using tips and videos. The researcher answered all questions by participants using phone calls or through text messages. After 6 weeks, the posttest was collected from all subgroups using phone calls. The nurse implemented an interactive lecture using PowerPoint presentations and discussions with participants to increase the level of dietary knowledge and practices. **Nutrition counseling messaging:** benefits of a healthy diet and using interactive learning are among the successful nutrition interventions. Examples of the topics covered are the physiological changes that occur in pregnant woman, the importance of diet and supplements for pregnant women and the fetus, the six categories of nutrition, the daily needs of diet during pregnancy, the normal weight gained during pregnancy, and the body mass index categories and its calculation. The teaching materials were adopted from ‚Healthy Eating during Pregnancy and Breastfeeding Nutrition of Women in the Preconception Period, during Pregnancy and the Breastfeeding Period and Diet during Pregnancy: Healthy Eating While Pregnant. **Intervention adapted to local context:** Minor modifications [questionnaire] were made to accommodate the Jordanian culture and achieve the study purpose. **Women empowerment approach:** Unclear **Control (*n* = 108)** **Description:** The control group received usual antenatal care in the two other health centers. No questions or discussions were opened about dietary knowledge and practices during pregnancy with the control group.
**Outcomes**	No outcomes of interest reported.
**Notes**	Funding: This study was funded by the Deanship of Research in Jordan University of Science and Technology.

Risk of bias

**Table:** 

Bias	Author's judgment	Support for judgment
Risk of bias arising from the randomization process	High Risk	Quasi‐randomized
Risk of bias due to deviations from the intended interventions (effect of assignment to intervention)	Some concerns	Study conducted in the health centers so carers are likely to know about the intervention.
Risk of bias due to deviations from the intended interventions (effect of adhering to intervention)	Some concerns	No information on blinding of participants or deviations that arose.
Missing outcome data	Low risk	Almost all the participants were included in the analysis, with no difference in dropouts between groups.
Risk of bias in measurement of the outcome	Low risk	Used structured questionnaire to collect outcome data. No information on awareness of outcome assessors.
Risk of bias in selection of the reported result	Low risk	All outcomes reported as specified in the methods.

Asiabar 2018

**Table:** 

**Methods**	Individual Randomized Controlled Trial in hospital center, Tehran, Iran
**Participants**	**Inclusion criteria:** The inclusion criteria were being a nulliparous female in the 10th to 12th week of pregnancy (based on the first day of the last menstrual cycle‐LMP or ultrasound result), single pregnancy, wanted pregnancy, lack of chronic diseases and any disease, which needs a particular nutritional pat‐tern before and during pregnancy, not having a vegan diet, not having a special diet, living permanently with one's spouse, and signing the written consent form. **Exclusion criteria:** The Exclusion criterion was having had miscarriage or abortion during pregnancy. **Age [Mean (SD)]—Intervention, Control:** 25.52(3.22), 26.70(3.77) **Unemployed: [*n* (%)]—Intervention, Control:** 33(75.0), 28(68.3) **Education: [*n* (%)]—Intervention, Control:** High school diploma or diploma: 15 (34.1), 20 (48.8) University graduate: 27 (61.4), 18 (43.9) Postgraduate: 2 (4.5), 3 (7.3) **Low income: [*n* (%)]—Intervention, Control:** 6 (13.6), 6 (14.6)
**Interventions**	**Intervention—Educational program (*n* = 50)** **Description:** The counseling sessions were structured focusing mainly on nulliparous female and nutritional dietary intake. The first session lasted up to 90 min and the second counseling session (90 min) reviewed the topics, which were mentioned in the first session yet in more depth and addressed selected aspects in a problem‐oriented manner. The control group received routine prenatal care only. During the study, he educational material on nutritional dietary intake was sent to the subjects via social messaging services ‚Telegram/Line, as all the participants had access to related applications. Telegram/Line education package included of 3–5 informative short messages as texts and figures about the kind of foods, the importance of dairy intake, vegetables, benefits of lipid reduction, especially saturated fats on the health of the mother and developing fetus and sufficiency of food supply during pregnancy. The service network frequently monitored whether the participants read the messages. Electronic learning continued during the 4 weeks addressing questions during the session. **Nutrition counseling messaging:** According to this booklet, focus on the essential dietary component was explained, including general topics, such as energy balance and a healthy nutrition. **Intervention adapted to local context:** Not reported **Women empowerment approach:** Unclear **Control (*n* = 50)** **Description:** received routine prenatal care, but no advice on dietary intake.
**Outcomes**	No outcomes of interest reported.
**Notes**	Funding: Authors have no direct financial interests that might pose a conflict of interest in connection with the submitted manuscript. The Tarbiat Modares University provided funding for this study.

Risk of bias

**Table:** 

Bias	Author's judgment	Support for judgment
Risk of bias arising from the randomization process	Some concerns	Randomized allocation but no information on allocation concealment. There was no significant differences at baseline.
Risk of bias due to deviations from the intended interventions (effect of assignment to intervention)	Some concerns	Single bind study with appropriate analysis used to estimate the effect of assignment to intervention.
Risk of bias due to deviations from the intended interventions (effect of adhering to intervention)	Low risk	Participants might have been aware of the study while personnel were blinded.
Missing outcome data	Low risk	Over 95% of participants were included in analysis.
Risk of bias in measurement of the outcome	Low risk	Outcomes were measured appropriately.
Risk of bias in selection of the reported result	Low risk	All outcomes reported appropriately.

Belizan 1995

**Table:** 

**Methods**	Individual Randomized Controlled Trial in Hospitals of Latin America (Rosario, Argentina, Pelotas, Brazil, Havana, Cuba, Mexico City, Mexico)
**Participants**	**Inclusion criteria:** Women initiating prenatal care between the fifteenth and twenty‐second weeks of gestation with singleton pregnancies; without clinical evidence of cardiovascular, renal, or other chronic diseases; history of cerclage. Rh‐negative, or mental diseases, but with at least one of the following risk factors were eligible for the study: (1) previous low‐birth‐weight or infant death; (2) previous fetal, neonatal or infant death (3) <17 years old; (4) body weight < 50 kg and height < 1.5; (5) low family income defined by locally adapted cutoff points; (6) <3 years of schooling; (7) smoking or heavy alcohol consumption; and (8) single, separated, divorced or widowed. **Exclusion criteria:** Not reported **Age [Mean (SD)]—Intervention, Control:** 24.3 (6.6), 24.6 (6.6) **White Race [*n* (%)]—Intervention, Control:** 669 (60.3), 609 (59.8) **Employed [*n* (%)]—Intervention, Control:** 330 (29.7), 309 (30.3) **Education (years) [Mean (SD)]—Intervention, Control:** 8.4 (3.7), 8.4 (3.8) **Low income [*n* (%)]—Intervention, Control:** (55.1), (54.9) **Married [*n* (%)]—Intervention, Control:** 509 (45.9), 486 (47.7)
**Interventions**	**Intervention—Nutrition counseling (*n* = 1110)** **Description:** The first part of the visit was devoted to encouraging the pregnant woman and her support person to discuss the pregnancy situation, changes, worries, and doubts. By using this information as background, the home visitor adapted the predefined themes, focusing the program on information that could be relevant to each woman. The home visitor discussed the developed strategy with the study supervisor after the first visit, and the final plan of the intervention for that woman, was developed. Changes were made, if needed, during subsequent visits. Activities during each visit and follow‐up recommendations were focused on strengthening the pregnant woman's social network, including from the first visit a support person selected by the patient to share with her all‐intervention activities. The support person could be the husband or partner, mother, sister, friend, or neighbor. The support person was encouraged to be involved throughout the pregnancy, to participate in the decision‐making process, helping the mother to resolve personal problems and promote healthy behavior and prenatal care attendance. The home visitor provided direct emotional support to the woman and helped her resolve problems related to the implementation of medical recommendations or prenatal care attendance. A special patient support office that did not require a previous appointment, with a hot line, was located at the hospitals only for patients in the intervention group. It was intended to help women with problems related to care attendance, the implementation of any indicated treatment or laboratory tests, and the monitoring of doctors’ recommendations. **Nutrition counseling messaging:** health education provided during home visit; this included education about nutrition, relevance and schedule of prenatal care, recognition of alarm signs, opportunity of hospital attendance, and suggestions about reducing smoking and alcohol or drug use. Educational activities conducted during the home visit were reinforced with a poster simulating a path for a healthy pregnancy and a booklet provided during the first home visit. These materials gave indications and encouragement, using familiar terms and situations, of different health and nutritional activities recommended during pregnancy. **Intervention adapted to local context:** The manual included detailed descriptions of various situations that home visitors were likely to encounter, with suggested interventions. It was intended that home visitors at the different study sites would act in a similar way, with adaptations appropriate to the local culture. **Women empowerment approach:** Partial empowerment model (i.e., including agency‐related activities only. **Control (*n* = 1019)** **Description:** The control group was provided with the routine prenatal care available at each of the participating institutions.
**Outcomes**	Anemia, hemorrhage, adherence to nutrition counseling, stillbirths, Low birthweight
**Notes**	Funding: Supported by a grant from the International Development Research Center, Ottawa, Canada.

Risk of bias

**Table:** 

Bias	Author's judgment	Support for judgment
Risk of bias arising from the randomization process	Low risk	A computer‐generated randomization code was used with balanced blocks of 20 women and stratified by center. A sequence of sealed opaque envelopes was used by a single investigator to assign women to intervention groups
Risk of bias due to deviations from the intended interventions (effect of assignment to intervention)	Some concerns	Clinic personnel were not made aware of the assigned intervention, but some women may have informed them. Due to the nature of the intervention participants were not blinded to assignment during the trial. The data were analyzed on an intention to treat basis.
Risk of bias due to deviations from the intended interventions (effect of adhering to intervention)	Some concerns	Adhering to the intervention was not assessed by the investigators. A special support office was created for women in the intervention group to encourage compliance, but adherence rates were not reported.
Missing outcome data	High risk	There are around 100 drop‐outs from each study arm, but missingness was not mentioned and no analysis to address this issue was conducted.
Risk of bias in measurement of the outcome	Low risk	Data for the treatment effects were collected by a team of independent professional interviewers who were not informed about the nature of the study.
Risk of bias in selection of the reported result	Low risk	data that produced this result was analyzed in accordance with a pre‐specified analysis plan that was finalized before unblinded outcome data were available for analysis.

Daniel 2016

**Table:** 

**Methods**	Non‐randomized Controlled Study, health facility in Bharuch district in Gujarat state, India
**Participants**	**Inclusion criteria:** Pregnant women with low BMI are defined as those pregnant women whose weight/height is less than 18.5 in the first trimester as on their ANC registration. **Exclusion criteria:** Not reported **Age [Mean (SD)]—Intervention, Control:** 23.2 (3.4), 24.8 (3.7)
**Interventions**	**Intervention—nutrition education (*n* = 54)** **Description:** In the intervention group, pregnant women with low BMI received nutrition education based on a field‐tested flipbook along with demonstration sessions on handwashing and meal preparation for a mean of 18 h over 9 months. Pregnant women in the intervention group were then educated and followed up by a health volunteer from their own community at random to ensure that they practice minimum meal frequency and diversity. Effect of nutrition education was assessed based on the practice of minimum meal frequency (3 meals a day), adoption of dietary diversity through 24‐h recall method, the proportion of change evidenced in handwashing practice and mean weight gain in the third trimester. **Nutrition counseling messaging:** information on food frequency and dietary diversity. **Intervention adapted to local context:** Not reported **Women empowerment approach:** Unclear empowerment approach **Control (*n* = 46)** **Description:** the non‐intervention group received regular entitlements through ICDS.
**Outcomes**	Gestational weight gain, low birth weight
**Notes**	Funding: No funding sources

Risk of bias

**Table:** 

Bias	**Author's judgment**
Representativeness of the exposed cohort	Somewhat representative of the average population of pregnant women in the community *
Selection of the nonexposed cohort	Drawn from the same community as the exposed cohort*
Ascertainment of exposure	Structured interview*
Demonstration that outcome of interest was not present at start of study	Yes*
Comparability of cohorts based on the design or analysis	Secure record (eg surgical records)*
Assessment of outcome	No description
Was follow‐up long enough for outcomes to occur	Yes*
Adequacy of follow up of cohorts	Complete follow up ‐ all subjects accounted for*

Demilew 2020

**Table:** 

**Methods**	Cluster Randomized Controlled Trial of 3 woredas (clusters) in West Gojjam zone (Bahir Dar Zuria, South Achefer, and Burie Zuria Woredas), Ethopia
**Participants**	**Inclusion criteria:** Pregnant women before 16 weeks of gestation who had planned to stay in the study area until delivery were enrolled in this trial. **Exclusion criteria:** Women without hypertension or diabetes mellitus. **Age [*n* (%)]—Intervention, Control:** Age < 20: 25 (8.0), 16 (4.8) 20–24: 53 (16.9), 74 (22.3) 25–29: 103(32.9), 87(26.2) 30–34: 74(23.7), 84(25.3) ≥35: 58 (18.5), 71 (21.4) **Housewife [*n* (%)]—Intervention, Control:** 149 (47.6), 183 (55.1) **Religion [*n* (%)]—Intervention, Control:** Muslim: 2 (0.6), 2 (0.6) Orthodox: 311 (99.4), 330 (99.4) **No formal education [*n* (%)]—Intervention, Control:** 260 (83.1), 262 (78.9) **Socioeconomic status [*n* (%)]—Intervention, Control:** Poorest: 56 (17.9), 60 (18.0) Poor: 72 (23.0), 67 (20.2) Medium: 65 (20.8), 62 (18.7) Rich: 56 (17.9), 78 (23.5) Richest: 64 (20.4), 65 (19.6) **Family size [*n* (%)]—Intervention, Control:** < 5: 215 (68.7), 240 (72.3) ≥5: 98(31.3), 92(27.7)
**Interventions**	**Intervention—Community‐based guided counseling (*n* = 346)** **Description:** The first counseling was given before 16 weeks of gestation, focused on basic nutrition, food groups, food selection, preparation, meal frequency, portion size, and iodized salt utilization. The second and third sessions of the counseling were given during the second trimester of pregnancy and covered the whole contents of the counseling guide. The last counseling was given based on the identified gaps during the early third trimester of pregnancy. Leaflets with the core messages in Amharic (local language) and appropriate pictures were prepared and delivered to each pregnant woman in the intervention arm. For women who could not read, anyone at home or in the neighborhood who could read was requested to read the leaflet to the woman and other family members. **Nutrition counseling messaging:** The core contents of the counseling guide were increasing meal frequency and portion size with increasing gestational age. Message on taking diversified meals by giving emphasis to iron‐rich foods, animal products, fruits, and vegetables was also one a component of the counseling guide. Messages on the consumption of iron/folic acid supplements and iodized salt were also included in the core contents of the counseling guide. Additional messages of the core contents were reducing heavy workload, taking day rest, impregnated bed net use and utilization of health care services. **Intervention adapted to local context:** During counseling, counselors used a client‐centered approach to identify women, their dietary practices and their specific needs in terms of nutrition. Leaflets with core messages in Amharic (local language) and appropriate pictures were prepared and delivered to each pregnant woman in the intervention group. **Women empowerment approach:** Unclear empowerment approach. **Control (*n* = 332)** **Description:** Women in the control group received nutrition education given by the health system. Pregnant women from both the control and intervention groups attended (antenatal clinic) ANC services.
**Outcomes**	**Macronutrient intake** during pregnancy
**Notes**	Funding: This research was funded by Bahir Dar University.

Risk of bias

**Table:** 

Bias	Author's judgment	Support for judgment
Risk of bias arising from the randomization process	Some concerns	Randomized allocation but no information on allocation concealment. There was no significant differences at baseline.
Risk of bias due to deviations from the intended interventions (effect of assignment to intervention)	Some concerns	No information on blinding for participants or personnel. Appropriate analysis was used to estimate the effect of assignment to intervention.
Risk of bias due to deviations from the intended interventions (effect of adhering to intervention)	Low risk	No information on blinding of participants but appropriate analysis used to estimate the effect of adherence to intervention.
Missing outcome data	Low risk	Complete data were obtained from 694 study participants, with a response rate of 97.5%.
Risk of bias in measurement of the outcome	Some concerns	Outcome assessors were probably aware of the intervention received by study participants.
Risk of bias in selection of the reported result	Low risk	outcomes were reported adequately.

Garg 2006

**Table:** 

**Methods**	Quasi‐randomized Controlled Study, Home visit/Community based in Anganwadi centers (AWCs) of the village, India
**Participants**	**Inclusion criteria:** pregnant women belonging to low‐SES and in varied periods of gestation ranging from completing their 5th month to completing their 9th month of gestation. **Exclusion criteria:** not reported. **Age [Mean (SD)]:** 24.46 (4.1) **Literacy:** More than half (57%) of the subjects were illiterate **Income:** The average per capita income was Rs. 699.5.
**Interventions**	**Intervention—Nutrition education (*n* = 50)** **Description:** The individualized counseling sessions ranged from about 30–40 min at the start of the intervention, where the subjects were asked to follow certain behaviors based on the lacunas identified at baseline. This was followed by weekly home visits to reinforce the messages, listen to subjects’ problems and check the compliance which included checking the IFA tablet strips and cross‐checking with family members if the subject was following the emphasized behaviors. The counseling sessions at the mid‐intervention lasted to about 20–30 min where the messages were again reinforced based on subjects’ 24‐h dietary recall, FFAQ and weight gain. Finally, towards the end of the intervention, the sessions lasted to about 15–20 min for further reinforcement. Monthly weighing of the subjects proved to be a motivating factor and helped to reinforce the messages. Whenever possible, the family members especially the husband and the mother‐in‐law of the subject were also encouraged to take extra care of the subject. **Nutrition counseling messaging:** (1) increasing quantity of food; (2) improving the quality of food; (3) promoting consumption of iron‐folic acid (IFA) tablets (4) rest during pregnancy; (5) Injection tetanus toxoid (TT) immunization and antenatal checkups (ANC) (getting 2 TT injections immunization completed at an interval of 1 month from ANM or doctor, undergoing periodic health check‐ups for weight gain, blood pressure and anemia)and (6) use of iodized salt (using iodized salt in all food preparations). **Intervention adapted to local context:** Not reported **Women empowerment approach:** Unclear empowerment approach. **Control (*n* = 50)** **Description:** The late‐gestation group without any intervention was referred to as non‐NE group for comparisons.
**Outcomes**	Anemia, Hemoglobin concentration, Adherence to Iron supplement consumption, Dietary intake during pregnancy
**Notes**	Funding: Not reported

Risk of bias

**Table:** 

Bias	Author's judgment	Support for judgment
Risk of bias arising from the randomization process	High risk	This was a quasi RCT with participants allocated to study arms without randomization or concealment
Risk of bias due to deviations from the intended interventions (effect of assignment to intervention)	Some concerns	Because of the nature of the intervention, participants and personnel were not blinded to the intervention assignment during the trial, but No reporting of any deviations from the intended intervention due to the trial context.
Risk of bias due to deviations from the intended interventions (effect of adhering to intervention)	Some concerns	The intervention seems to have been implemented properly but no discussion of challenges in implementation, but their statistical analysis was not weighted to adherence and did not consider attrition rates.
Missing outcome data	High risk	Data for this outcome was not available for all participants and no analysis to address this issue was conducted.
Risk of bias in measurement of the outcome	Low risk	The method of measuring the outcome was appropriate but the assessment of the outcomes could have been influenced by knowledge of intervention assignment.
Risk of bias in selection of the reported result	Low risk	All their analysis plans were prespecified before data was available.

Goodarzi‐Khoigani 2018

**Table:** 

**Methods**	Individual Randomized Controlled Trial of 15 community health centers, 5 hospitals, and 15 private offices in Isfahan, Iran
**Participants**	**Inclusion criteria:** Included gestational age between 6 and 10 weeks, body mass index (BMI) < 40 kg/m^2^, a history of nonsmoking, age18 and older, Iranian by origin, and singleton pregnancy. **Exclusion criteria:** Women with weight‐related complications, a history of diabetes (diabetes mellitus type1 and type2), mental disease, anemia, urinary tract complications, usage of a special regimen, chronic disease, addiction as well as the women who did not participate in all the classes because of medical or other reasons were excluded. **Age [Mean (SD)]—Intervention, Control:** 26.31 (3.99), 26.83 (3.89) **Education [*n* (%)]—Intervention, Control:** Diploma and< diploma: 30 (34.09), 31 (36.05) Undergraduate: 49 (55.68), 50 (58.14) Postgraduate: 9 (10.32), 5 (5.81) **Socioeconomic status (Iranian Rial) [*n* (%)]—Intervention, Control:** <600,000,019: (21.59), 17 (19.77) 6,000,000–12,000,000: 48 (54.55), 54 (62.79) >12,000,000: 21 (3.86), 15 (17.44)
**Interventions**	**Intervention—Nutrition education (*n* = 96)** **Description:** The nutrition education was based on Pender‚ included three 45‚ 60 min training sessions in 6‚ 10, 18, and 26 weeks of pregnancy. In the first session, the dietary pattern, including the average daily servings of five food groups, was explained to the participants. The food groups were (i) grain (cereal), mostly whole grains;(ii) milk, yogurt, cheese, and/oral alternatives (mostly reduced fat);(iii) lean meat and poultry, fish, eggs, nuts and seeds, and legumes/beans; (iv) fruits; and (v) vegetables. One booklet, which included the benefits of their commended points, the barriers to implementation, and the ways to overcome these barriers during pregnancy, was given to each of the participants in the experimental group. Each participant was requested to record her daily dietary food intakes monthly to develop a commitment toward a plan. They were also requested to record the daily food portions form and keep this record with them for use in future sessions. These data and responses to questions about the leaflet‚ and points were used to examine the participants‚ compliance and to give individualized feedback to each woman as needed. Except for the first session, pregnant mothers were divided into groups, including 3–8 persons who discussed their opinions about recommended points (through role playing and brainstorming). In the second session, practical steps (goal setting techniques) to increase self‐efficacy were taught to the mothers in the experimental group. Except for the first session, pregnant mothers were divided into groups, including 3–8 persons who discussed their opinions about recommended points (through role playing and brainstorming). In the second session, practical steps (goal setting techniques) to increase self‐efficacy were taught to the mothers in the experimental group. Positive and negative feelings toward the dietary pattern were discussed by mothers. In a training session, the researcher explained to the participants, husbands, mothers, and mothers‐in‐law about the role of nutrition in improving the outcome of the pregnancy and the influencing factors of consumption (such as the availability of healthy foods at home). **Nutrition counseling messaging:** One booklet, which included the benefits of their commended points, the barriers to implementation, and the ways to overcome these barriers during pregnancy. Positive and negative feelings toward the dietary pattern were discussed by mothers. **Intervention adapted to local context:** Not reported **Women empowerment approach:** Unclear empowerment approach. **Control (*n* = 96)** **Description:** ANC
**Outcomes**	No outcomes of interest reported.
**Notes**	Funding: Public Health College of Shahid Sadoughi University of Medical Sciences, Yazd, Iran

Risk of bias

**Table:** 

Bias	Author's judgment	Support for judgment
Risk of bias arising from the randomization process	High risk	Randomization occurred in consecutive order at the time of enrollment.
Risk of bias due to deviations from the intended interventions (effect of assignment to intervention)	Low risk	Midwives and physicians were blinded to the subject randomization and the educational content of the study to prevent contamination. Computer‑generated codes were sealed in consecutively numbered opaque envelopes and concealed from the investigator by a responsible person who had no other involvement in the study.
Risk of bias due to deviations from the intended interventions (effect of adhering to intervention)	Low risk	Participants and carer were not aware of the intervention.
Missing outcome data	High risk	Outcome data were not available for all participant, and it is likely that missingness in the outcome depends in its true value since women dropping from the intervention group.
Risk of bias in measurement of the outcome	Low risk	The outcome was measured using a standardized Pender construct.
Risk of bias in selection of the reported result	Low risk	All the reported measures belong to the Pender construct.

Hasneezah 2020

**Table:** 

**Methods**	Non‐randomized Controlled Study in Salak and Dengkil health clinics, Sepang District, Malaysia
**Participants**	**Inclusion criteria:** The sampling population is pregnant women in both clinics diagnosed as having anemia in pregnancy with Hemoglobin(Hb) levels of less than 11.0 g/dL and meet all the inclusion criteria. Subjects who met the following inclusion criteria were selected: Malaysian, singleton pregnancy, Hb level between 7.0 and 11 g/dL, came for antenatal booking before 24 weeks of pregnancy and wished to continue her antenatal check‐up at these clinics. **Exclusion criteria:** Known cases of anemia secondary to hematological disorders, women with severe anemia (Hb < 7 g/dL) and twin pregnancies were excluded. **Age [Mean (SD)]—Intervention, Control:** 30.1 (4.84), 29.23 (5.05) **Race/Ethnicity [*n* (%)]—Intervention, Control:** Malay: 57 (70.3), 62 (76.5) Chinese: 8 (9.9), 1 (1.2) India: 12 (14.8), 14 (17.3) Others: 4 (4.9), 4 (4.9) **Employment status [*n* (%)]—Intervention, Control:** Working: 58 (54.7), 48 (45.3) Not working: 23 (41.1), 33 (58.9) **Education level (Finished sec school) [*n* (%)]—Intervention, Control:** 50 (56.2), 39 (43.8) **Income [*n* (%)]—Intervention, Control:** 0–2999: 49 (51.6), 46 (48.4) 3000 and above: 32 (47.8), 35 (52.2)
**Interventions**	**Intervention—HBM‐based health education intervention (*n* = 81)** **Description:** The intervention program was developed through the process of consultations with a group of experts including two public health specialists and one family medicine specialist, studying the relevant literature and received opinions from the community being served. The health talk was given once for each participant during the intervention period in the form of power point presentation for about 1 h. The second activity of the health education intervention programme was as small group discussions. This was conducted and facilitated by the researcher between two to 4 weeks after the health talk. The participants were divided into nine small groups with eight to ten participants in one group. Each group participated one session during the intervention period based on timing of the participants. This activity dealt with the issues to enhance the compliance towards iron supplementation and to increase the dietary iron intake by identifying barriers towards anemia preventive behaviors. The participants were also provided with a checklist on compliance for iron supplementation. The materials used in the health education intervention programme were posters and pamphlets. **Nutrition counseling messaging:** The HBM‐based program addresses four major components for compliance with recommended health action: perceived barrier of recommended health action, perceived benefits of recommended health action, perceived susceptibility of the disease and perceived severity of the disease. The health talk, given by the researcher, was on topics such as introduction to anemia in pregnancy, predisposing factors, signs and symptoms, complications, prevention of anemia in pregnancy, knowledge of the wrong perception of anemia and iron supplementation, misperception about protein‐rich food and food taboos during pregnancy, and knowledge of the various sources of food that contain high iron. The pamphlet also helped to deliver take‐home messages to the participants about anemia in pregnancy. It was covered the information regarding the definition, causes, complications, and prevention of anemia in pregnancy and examples of food rich of iron. These materials were also aimed to maintain adherence towards anemia preventive behavior. **Intervention adapted to local context:** Not reported **Women empowerment approach:** Unclear empowerment approach. **Control (*n* = 81)** **Description:** Regular treatment.
**Outcomes**	Hemoglobin concentration, Adherence to Iron supplement consumption,
**Notes**	Funding: This research was funded by the Putra IPS Grant (GP‐IPS/2017/9576900) from Universiti Putra Malaysia.

Risk of bias

**Table:** 

Bias	Author's judgment	Support for judgment
Risk of bias arising from the randomization process	High Risk	Quasi‐randomized
Risk of bias due to deviations from the intended interventions (effect of assignment to intervention)	Some concerns	Unlikely to be double blind. There is no information on deviations that arise because of trial context.
Risk of bias due to deviations from the intended interventions (effect of adhering to intervention)	Low risk	Even though the included women were anemic, both groups received routine antenatal care which included iron supplementation from their respective clinics.
Missing outcome data	Low risk	Almost all the participants were included in the analysis, with no difference in dropouts between groups.
Risk of bias in measurement of the outcome	Low risk	The method measuring the outcome was not inappropriate and the measurement method did not differ between intervention groups.
Risk of bias in selection of the reported result	Some concerns	Multiple analyses of the same data ‐ did not report unadjusted.

Izadirada 2017

**Table:** 

**Methods**	Individual Randomized Controlled Trial, health centers in Iranshahrm Baluchistan region, Iran
**Participants**	**Inclusion criteria:** first pregnancy, wanted pregnancy, first marriage, monogamy, aged between 18 and 35 years, being in the second trimester of pregnancy, residence in the city of Iranshahr, having health care records, and being healthy. **Exclusion criteria:** Exclusion criteria included abortion, being transferred to another city and nonattendance in the training sessions. **Age [Mean (SD)]: ‐ Intervention, Control:** 20.78 (2.18), 2.1 (2.97) **Occupation [*n* (%)]: ‐ Intervention, Control:** Housewife: 71 (91.1), 70 (93.3) Employee: 69 (8.88), 50 (6.66) **Education [*n* (%)]: ‐ Intervention, Control:** 0–6 years (primary): 24 (31.11), 28 (37.77) 7–12 years (secondary): 28 (35.55), 33 (44.44) >12 years (college): 26 (33.33), 13 (17.77) **Income [*n* (%)]: ‐ Intervention, Control:** Very low (140$<): 45 (57.77), 50 (66.66) Low ($140 to $280): 21 (26.66), 17 (22.22) Average (280$>): 12 (15.55), 8 (11.11)
**Interventions**	**Intervention—Educational intervention (*n* = 78)** **Description:** The educational intervention comprised three sessions of 50 to 60 min each, using lectures, question and answers, group discussion, and role playing also benefiting from reliable sources of the Ministry of Health, Education. The control group received routine training at the care centers. **Nutrition counseling messaging:** The first session of the educational intervention included introduction to prenatal care, the right time to start prenatal care, hygiene, danger signs, frequently occurring disorders and high‐risk pregnancies. The second and the third sessions, based on the Health Belief Model, contained education regarding potential complications of pregnancy (including abortion, maternal death, fetal death, low birth weight, etc.), increasing the mother's perceived benefits regarding receiving prenatal care, as well as efforts to reduce barriers perceived by mothers for receiving prenatal care by highlighting the perceived benefits (to reduce the difficulty of obstacles to them). Additionally, to boost self‐efficacy, the (increasing maternal skills, using successful models, such as the experiences of other mothers who have successfully performed prenatal care in their first pregnancy in the same community with the same conditions, educational videos, setting schedules and verbal encouragement) participants in the intervention group received a training manual. **Intervention adapted to local context:** Not reported **Women empowerment approach:** Partial empowerment model **Control (*n* = 75)** **Description:** The control group received routine training at the care centers.
**Outcomes**	No outcomes of interest reported.
**Notes**	Funding: This study is a result of a health education and health promotion doctoral thesis approved by the health faculty of Tarbiat Modares University in Tehran. Hereby, we would like to thank the Tarbiat Modares University and Iranshahr Health Center and all the participants in this study.

Risk of bias

**Table:** 

Bias	Author's judgment	Support for judgment
Risk of bias arising from the randomization process	Some concerns	No information allocation concealment but no baseline imbalance to suggest a problem.
Risk of bias due to deviations from the intended interventions (effect of assignment to intervention)	Low risk	This trial was conducted in a single blind fashion in which the statistical analyst was not aware of the allocation of the women to the intervention or control groups, but the observers and participants were aware of their group assignment.
Risk of bias due to deviations from the intended interventions (effect of adhering to intervention)	Some concerns	Participants were probably aware of the intervention.
Missing outcome data	Low risk	All participants were included in the analysis.
Risk of bias in measurement of the outcome	Low risk	The outcome was measured using the standardized Health Belief Model constructs.
Risk of bias in selection of the reported result	Low risk	All the reported measures belong to the Health Belief Model.

Jahan 2014

**Table:** 

**Methods**	Individual Randomized Controlled Trial in antenatal clinics, Bashbari, Dhaka, Bangladesh
**Participants**	**Inclusion criteria:** Women at a gestational age of 24 weeks attending the government Maternal and Child Health Training Institute, Azimpur, and the Marie Stopes Clinic, Bashbari, Dhaka, were invited to participate in the study. **Exclusion criteria:** Women with complications and special requirements were excluded. **Age [Mean (SD)]—Intervention, Control:** 23.45 (3.5), 23.55 (3.54) **Monthly family income (US$) [Mean (SD)]—Intervention, Control:** 176 (45), 166 (68)
**Interventions**	**Intervention—Nutrition education (*n* = 192)** **Description:** The investigators developed a manual to provide nutrition education to the women containing principles of the UNICEF proposed nutrition triangle, including food security, caring practices, and disease control. Nutrition education was provided in the outpatient areas of clinics to groups of six to eight women for 1 h each month over a 3‐month period. **Nutrition counseling messaging:** The pregnant women and any accompanying family members were educated on the significance of pregnancy weight gain, adequate food intake, and breastfeeding for the newborn. Nutrition education emphasized specific key messages, including increasing the frequency of food intake from the usual three to five times daily, as well as preparing and consuming a specific, nutrient‐rich local food (khichuri). The women were given advice on food intake, personal hygiene, rest during the daytime, accessing antenatal care services, initiation of breastfeeding immediately after birth, and continuation of exclusive breastfeeding. The benefits of adequate energy, protein, and vitamins during pregnancy were explained. The benefit of regular iron intake was also emphasized. **Intervention adapted to local context:** Not reported **Women empowerment approach:** Partial empowerment model. **Control (*n* = 192)** **Description:** The intervention group received nutrition education and the control group received routine hospital services.
**Outcomes**	Gestational weight gain, timely initiation of breastfeeding
**Notes**	Funding: Not reported

Risk of bias

**Table:** 

Bias	Author's judgment	Support for judgment
Risk of bias arising from the randomization process	Some concerns	Randomized allocation but no information on allocation concealment. There were no significant differences at baseline.
Risk of bias due to deviations from the intended interventions (effect of assignment to intervention)	Some concerns	No report on blinding.
Risk of bias due to deviations from the intended interventions (effect of adhering to intervention)	Some concerns	Participant blinding not reported and outcome assessors.
Missing outcome data	Low risk	Data incomplete however reasons were provided and unlikely that missing effects true value.
Risk of bias in measurement of the outcome	Low risk	Appropriate measure of outcome with same number of outcome measurements.
Risk of bias in selection of the reported result	Low risk	Outcomes were reported adequately.

Jahangiri 2020

**Table:** 

**Methods**	Individual Randomized Controlled Trial in urban healthcare centers in Arak City, Iran.
**Participants**	**Inclusion criteria:** The inclusion criteria of the research included literate pregnant women who were at the beginning of the fourth month of pregnancy and had a case in health centers in Arak; not having an underlying disease related to anemia; not having anemia, and satisfaction to participate in the study. Before the intervention, pre‐test information was collected from both study groups. **Exclusion criteria:** Not reported. **Occupation [*n* (%)]—Intervention, Control:** Housewife: 34 (85), 35 (88) Student: 1 (3), 0 (0) Independent staff: 0 (0), 1 (3) Employee: 5 (13), 4 (10) **Education [*n* (%)]—Intervention, Control:** Primary: 5 (13), 4 (10) Secondary: 21 (53), 24 (60) University: 14 (35), 12 (30) **Monthly family income (US$) [Mean (SD)]—Intervention, Control:** 176 (45), 166 (68)
**Interventions**	**Intervention—Nutrition counseling (*n* = 40)** **Description:** Then, the educational intervention was performed in the experimental group in 1 month and 4 training sessions, based on the TPB. The obtained results were analyzed 3 months after the intervention. **Nutrition counseling messaging:** Not described **Intervention adapted to local context:** Not reported **Women empowerment approach:** Unclear empowerment approach. **Control (*n* = 40)** **Description:** Not described
**Outcomes**	No outcomes of interest reported.
**Notes**	Funding: This study was extracted from the MSc. thesis of the first author at the Department of Health Promotion and Education, Faculty of Health, Arak University of Medical Sciences, Arak. And it was supported by Arak University of Medical Sciences

Risk of bias

**Table:** 

Bias	Author's judgment	Support for judgment
Risk of bias arising from the randomization process	Some concerns	There is no information about concealment of the allocation sequence and there were no differences between the study groups for demographic variables.
Risk of bias due to deviations from the intended interventions (effect of assignment to intervention)	High risk	There is no information on whether the participants and carers were aware of the intervention groups.
Risk of bias due to deviations from the intended interventions (effect of adhering to intervention)	High risk	There is no information on whether the participants and carers were aware of the intervention groups as well as no information on adherence.
Missing outcome data	High risk	There is no information on the missingness.
Risk of bias in measurement of the outcome	High risk	There is no information who assessed the outcomes or details on the intervention to inform the possibility of the intervention being influenced by external factors.
Risk of bias in selection of the reported result	Some concerns	There is no information on the planned analysis of the authors and the framework used to develop the questionnaire.

Jing 2015

**Table:** 

**Methods**	Individual Randomized Controlled Trial in West China Second University Hospital, Chengdu, China
**Participants**	**Inclusion criteria:** Eligible women were aged at least 18 years, could understand the written Chinese language, and did not have pre‐existing diabetes. **Exclusion criteria:** Women with any pregnancy‐related complications or general medical disorders not associated with pregnancy were excluded. **Age [Mean (SD)]—Intervention, Control:** 29.57 (4.13), 9.89 (3.86)
**Interventions**	**Intervention—Personalized intervention (*n* = 131)** **Description:** In addition to standard health education manuals, women in the intervention group received an education manual on diet and physical activity (written by the research team) after randomization, and one‐to‐one counseling for at least 20 min in a private room with a trained graduate student (W.J.) after group assignment and at 16–20 and 20–24 weeks. The same graduate student was also available to answer questions and provide feedback on diet and physical activity behavior until 20–24 weeks either over the phone or via a group established on Recent instant messenger (generally known as QQ in China) specifically for the present study. By contrast, pregnant women in the control group received only conventional interventions, such as standard health education manuals produced by the hospital. **Nutrition counseling messaging:** The key points of education included the harm of GWG and GDM, the benefit of encouraged behaviors, the difficulty to change habits, and the importance of belief in the efficacy of the interventions. **Intervention adapted to local context:** Not reported **Women empowerment approach:** Unclear empowerment approach. **Control (*n* = 131)** **Description:** The control group received only conventional interventions, such as standard health education manuals produced by the hospital.
**Outcomes**	Gestational weight gain, **Macronutrient intake** during pregnancy
**Notes**	Funding: Not reported

Risk of bias

**Table:** 

Bias	Author's judgment	Support for judgment
Risk of bias arising from the randomization process	Some concerns risk	No information on how the randomization was conducted. “Participants and data analysts were masked to group assignment.”
Risk of bias due to deviations from the intended interventions (effect of assignment to intervention)	Low	healthcare worker delivering the intervention was aware of the participants assigned to the trial however there were no deviations reported.
Risk of bias due to deviations from the intended interventions (effect of adhering to intervention)	Some concerns	Participants were possibly aware of their assigned intervention as well as the people delivering the intervention.
Missing outcome data	Low risk	Data was reported for most participants. Reason for missing participants was reported.
Risk of bias in measurement of the outcome	Some concerns	outcome measures were appropriate however assessors were not blinded.
Risk of bias in selection of the reported result	Low risk	All outcomes were measured as stated in methods

Kafatos 1989

**Table:** 

**Methods**	Cluster Randomized Controlled Trial of home visits in Florina, Greece
**Participants**	**Inclusion criteria:** women residing in the market towns and villages of Florina, Greece. **Exclusion criteria:** Not reported. **Age [Mean (SD)]—Intervention, Control:** 23.17 (0.31), 22.92 (0.31)
**Interventions**	**Intervention—Nutrition intervention program (*n* = 300)** **Description:** Nutrition education for the 300 women in the intervention group was provided through home visits every 2 week by the 10 nurses employed at the intervention group clinics, who had received intensive training for this purpose. **Nutrition counseling messaging:** basics of nutrition during pregnancy for the health of mother and fetus, including food sources and nation effects of selecting a balanced diet. The women were also taught practical techniques for improving the quality of their diets. They were encouraged to consume locally grown foods that have a high nutrient value and to prepare and preserve food in such a way as to reduce the loss of nutrients. **Intervention adapted to local context:** Not reported **Women empowerment approach:** Unclear empowerment approach. **Control (*n* = 268)** **Description:** Not described.
**Outcomes**	Hemoglobin, dietary intake, stillbirths, low birthweight, small for gestational Age (SGA)
**Notes**	Funding: Computer analysis of nutrient intake data was done by G Christakis and J Cassady of the Nutrition and Biostatistics Division, Department epidemiology, University of Miami School of Medicine. SN Pantelakis of the Agia Sophia Pediatric Hospital, Athens, and SA Doxiadis, Director of the Foundation for Research in Childhood, Athens, made substantive contributions in implementing the nutrition education program.

Risk of bias

**Table:** 

Bias	Author's judgment	Support for judgment
Risk of bias arising from the randomization process	Some concerns	Centers were randomized but no explanation of the randomization process, no reporting of allocation conceallment. Baseline characteristics were balanced except for maternal weight, suggesting appropriate randomization.
Risk of bias due to deviations from the intended interventions (effect of assignment to intervention)	High risk	Because of the nature of the intervention, participants and personnel were made aware of their assignment during the trial, and no reporting of analysis to determine the effect of intervention assignment.
Risk of bias due to deviations from the intended interventions (effect of adhering to intervention)	High risk	There were difficulties experienced by the nurses and the program's coordinator in carrying out home visits during the winter months, when many of the county's mountainous villages are cut off by snow for days or weeks at a time, and no reporting of special analysis.
Missing outcome data	High risk	Incomplete sets of assessments were imbalanced across study arms, analysis therefore only focused on paired intergroup comparisons in dietary Intake at different points in time.
Risk of bias in measurement of the outcome	High risk	Nurses who delivered the intervention measured the outcomes. However, to minimize the possible bias associated with the nurses simultaneously delivering nutrition counseling and recording the effects in the intervention group (in terms of dietary histories and weight gain), the program's nurse coordinator periodically accompanied each nurse on their home visits to observe the data gathering and to sort out any associated problems.
Risk of bias in selection of the reported result	Low risk	data that produced this result was analyzed in accordance with a pre‐specified analysis plan that was finalized before unblinded outcome data were available for analysis.

Kamalifard 2012

**Table:** 

**Methods**	Non‐randomized Controlled Study of four centers in the south and southeast of Karaj, Iran
**Participants**	**Inclusion criteria:** women with a prenatal care record, with a gestational age of 8–14 weeks, with a minimum literacy rate, who intended to live in Karaj during pregnancy, were included in the study from August to September 2011. **Exclusion criteria:** The person had a history of previously known illnesses such as cardiovascular, renal, respiratory, gastrointestinal, blood, autoimmune, diabetes, thyroid, epilepsy, chronic hypertension, and cancer. Abortion, multiple births, cerclage, preterm labor, pre‐eclampsia and a history of infertility, people using alcohol and drugs and antidepressants, and people on a diet specific to a chronic systemic disease, as well as people who are earlier than 6 weeks later. They were excluded from the study due to the termination of their pregnancy for any reason. **Age [*n* (%)]—Intervention, Control:** ‚≤19: 5 (12), 5 (12.2) 20–34: 32 (76), 33 (80.5) ≥35: 5 (12), 3 (7.3) **Housewife [*n* (%)]—Intervention, Control:** 40 (95.2), 38 (92.7) **Education [*n* (%)]—Intervention, Control:** Elementary and middle school: 13 (31), 17 (41.5) High school: 22 (52.5), 19 (46.3) University: 7 (16.5), 5 (12.2) **Income less than 500 thousand tomans [*n* (%)]—Intervention, Control:** 22 (52.5), 25 (61)
**Interventions**	**Intervention—Nutrition intervention program (*n* = 44)** **Description:** The sessions of each class were separate and at separate times. These classes were held in groups, with a minimum of 5 and a maximum of 11 people, in two sessions of approximately 60–90 min once a week for two consecutive weeks for each selected group of health centers. The presentation of an educational video as well as an educational booklet, which was given to mothers at the end of the first session, was used. Mothers were instructed to read the booklet within a week of the start of the second session. At the beginning of the second session, the researcher addressed the ambiguities of mothers. To answer the possible questions of the experimental group, a contact number was given to them. The content of the educational booklet is based on the basic information of scientific texts related to nutrition during pregnancy, including the book compiled by the Ministry of Health for health workers, and based on the initial assessment of the educational needs of pregnant women by asking open and closed questions. The pre‐intervention stage was developed. The general content of the training, after being prepared and approved by the professors of midwifery and nutrition, was prepared in a simple language and in a way that could be understood by the mothers. **Nutrition counseling messaging:** The content of nutrition education in the first session includes Familiarity with the main food groups and their recommended amounts in pregnancy and lactation, observing diversity and balance in the diet, improving eating habits and behavior, and explaining how the mother weighs in pregnancy, based on mass index It was the mother's body. To inform mothers about how to gain weight during pregnancy, the body mass index of each pregnant mother was calculated using height and weight before or early pregnancy and was marked in the relevant section of each person's educational booklet. The educational content of the second session included: how to take a variety of dietary supplements, vitamins and minerals during pregnancy and lactation, familiarity with common problems during pregnancy and advice on how to deal with them, as well as nutritional tips for dealing with complications and diseases during pregnancy. The final 30 min in each session were devoted to questions and answers. **Intervention adapted to local context:** Not reported **Women empowerment approach:** Unclear empowerment approach. **Control (*n* = 44)** **Description:** The control group received another training with completely different content and did not affect the outcome of this study, with approximately the same number of sessions and duration, which by asking Experts in this field, the control group received training in almost the same way in the field of sexual health.
**Outcomes**	No outcomes of interest reported.
**Notes**	Funding: This article has been extracted from a student dissertation, the design of which has been approved by Tabriz University of Medical Sciences with the number 9014 on 2/14/2011. Due to the assistance and cooperation of the Vice Chancellor for Research and the Faculty of Nursing and Midwifery of Tabriz University of Medical Sciences due to the financial and spiritual support of this project, the cooperation of Karaj University of Medical Sciences and Alborz health centers in collecting data.

Risk of bias

**Table:** 

Bias	Author's judgment	Support for judgment
Risk of bias arising from the randomization process	Some concerns	Centers were randomized but no explanation of the randomization process, no reporting of allocation conceallment. Baseline characteristics were balanced except for maternal weight, suggesting appropriate randomization.
Risk of bias due to deviations from the intended interventions (effect of assignment to intervention)	High risk	Because of the nature of the intervention, participants and personnel were made aware of their assignment during the trial, and no reporting of analysis to determine the effect of intervention assignment.
Risk of bias due to deviations from the intended interventions (effect of adhering to intervention)	High risk	There were difficulties experienced by the nurses and the program's coordinator in carrying out home visits during the winter months, when many of the county's mountainous villages are cut off by snow for days or weeks at a time, and no reporting of special analysis.
Missing outcome data	High risk	Incomplete sets of assessments were imbalanced across study arms, analysis therefore only focused on paired intergroup comparisons in dietary Intake at different points in time.
Risk of bias in measurement of the outcome	High risk	Nurses who delivered the intervention measured the outcomes. However, to minimize the possible bias associated with the nurses simultaneously delivering nutrition counseling and recording the effects in the intervention group (in terms of dietary histories and weight gain), the program's nurse coordinator periodically accompanied each nurse on their home visits to observe the data gathering and to sort out any associated problems.
Risk of bias in selection of the reported result	Low risk	data that produced this result was analyzed in accordance with a pre‐specified analysis plan that was finalized before unblinded outcome data were available for analysis.

Kanber 2011

**Table:** 

**Methods**	Individual Randomized Controlled Trial in Obstetrics and Gynecology Hospital and the University Hospital in the city center, Afyonkarahisar, Turkey
**Participants**	**Inclusion criteria:** Not reported **Exclusion criteria:** Pregnant women with chronic anemia, chronic kidney disease, parasitic disease, and illiterate people were not included in the study. **Age [*n* (%)]—Intervention, Control:** under 25 years old: 10 (3.3), 14 (46.7) 25 years and older: 20 (66.7), 16 (53.3) **Occupation [*n* (%)]—Intervention, Control:** Not working: 22 (73.3), 24 (80) Working: 8 (26.7), 6 (20) **Education status [*n* (%)]—Intervention, Control:** Primary education: 15 (50), 17 (56.7) Lesesne: 9 (30), 8 (26.7) University: 6 (20), 5 (16.7) **Monthly Income Level [*n* (%)]—Intervention, Control:** Below 500 Turkish Lira: 10 (33.3), 10 (33.3) Above 500 Turkish Lira: 20 (66.7), 20 (66.7) **Social security [*n* (%)]—Intervention, Control:** 2 (6.7), 1 (3.3)
**Interventions**	**Intervention—Nutrition education (*n* = 30)** **Description:** In our study, the questionnaire form was first applied to the pregnant women by face‐to‐face interview technique, and then the information prepared in the training form was explained to the EG pregnant, and these pregnant women were reminded by phone once a month to pay attention to their nutrition. **Nutrition counseling messaging:** No information. **Intervention adapted to local context:** Not reported. **Women empowerment approach:** Unclear empowerment approach. **Control (*n* = 30)** **Description:** Not reported.
**Outcomes**	Hemoglobin concentration
**Notes**	Funding: Not reported

Risk of bias

**Table:** 

Bias	Author's judgment	Support for judgment
Risk of bias arising from the randomization process	High risk	There is no information about randomization process and there are baseline imbalances
Risk of bias due to deviations from the intended interventions (effect of assignment to intervention)	High risk	There is no information on whether the participants and carers were aware of the intervention groups
Risk of bias due to deviations from the intended interventions (effect of adhering to intervention)	High risk	There is no information on whether the participants and carers were aware of the intervention groups as well as no information on adherence.
Missing outcome data	High risk	There is no information on the missingness or follow up of participants from eligibility to analysis
Risk of bias in measurement of the outcome	High risk	There is no information who assessed the outcomes or details on the intervention to inform the possibility of the intervention being influenced by external factors.
Risk of bias in selection of the reported result	Some concerns	There is no information on the planned analysis of the authors and the framework used to develop the questionnaire

Katenga‐Kaunda 2021

**Table:** 

**Methods**	Cluster Randomized Controlled Trial, Namkumba area in Mangochi, one of the 12 districts in the southern region of Malawi.
**Participants**	**Inclusion criteria:** All consenting pregnant women who were between their 9th and 16th gestational week, both primi‐ and multiparous, were eligible for inclusion. **Exclusion criteria:** Women carrying multiple fetuses and those with severe illnesses were excluded from the study. **Age [Median (IQR)]: ‐ Intervention, Control:** 24 (19–30), 23 (18–28) **Geographic location [*n* (%)]: ‐ Intervention, Control:** Lakeshore: 43 (46.7), 60 (58.3) Upland: 49 (53.3), 43 (41.7) **Household head gender [n (%)]: ‐ Intervention, Control:** Female: 7 (4.3), 9 (11.7) Male: 85 (95.7), 94 (88.3) **Education [*n* (%)]: ‐ Intervention, Control:** Illiterate/primary: 79 (83.7), 79(78.6) Secondary/above: 13 (16.3), 24(21.4) **Socioeconomic status [*n* (%)]: ‐ Intervention, Control:** Very‐poor: 31 (33.7), 29 (28.2) Poor: 39 (42.4), 43 (41.7) Well‐off: 22 (23.9), 31 (30.1) **Marital status [*n* (%)]: ‐ Intervention, Control:** Single: 9 (4.3), 12 (16.5) Married: 83 (95.7), 91 (83.5),
**Interventions**	**Intervention—Nutrition education (*n* = 121)** **Description:** Nutrition education for the 300 women in the intervention group was provided through home visits every 2 weeks by the 10 nurses employed at the intervention group clinics, who had received intensive training for this purpose. **Nutrition counseling messaging:** Basics of nutrition during pregnancy for the health of mother and fetus, including food sources and nation effects of selecting a balanced diet. The women were also taught practical techniques for improving the quality of their diets. They were encouraged to consume locally grown foods that have a high nutrient value and to prepare and preserve food in such a way as to reduce the loss of nutrients. **Intervention adapted to local context:** Not reported **Women empowerment approach:** Partial empowerment model **Control (*n* = 136)** **Description:** Not described
**Outcomes**	No outcomes of interest reported.
**Notes**	Funding: This project was funded by the University of Oslo, The Global Health and Vaccination Program (GLOBVAC) of the Research Council of Norway and by the Throne Holst Foundation.

Risk of bias

**Table:** 

Bias	Author's judgment	Support for judgment
Risk of bias arising from the randomization process	Low risk	The villages were assigned STATA generated random numbers to allocate them to either the intervention or the control group, generating 10 clusters in each study group. The women from these clusters were identified by the study team in their communities and referred to the nearest designated local health center where their pregnancy and the gestational age were confirmed and where they were also invited to consent to participate in the study.
Risk of bias due to deviations from the intended interventions (effect of assignment to intervention)	High risk	There is no information on whether the participants and carers were aware of the intervention groups.
Risk of bias due to deviations from the intended interventions (effect of adhering to intervention)	High risk	There is no information on whether the participants and carers were aware of the intervention groups as well as no information on adherence.
Missing outcome data	High risk	There is no information on the missingness or follow up of participants from eligibility to analysis
Risk of bias in measurement of the outcome	High risk	There is no information who assessed the outcomes or details on the intervention to inform the possibility of the intervention being influenced by external factors.
Risk of bias in selection of the reported result	Some concerns	There is no information on the planned analysis of the authors and the framework used to develop the questionnaire

Khoramabadi 2015

**Table:** 

**Methods**	Individual Randomized Controlled Trial in therapeutic‐health centers (Level I) Shahid Beheshti University of Medical Sciences in Tehran, Iran
**Participants**	**Inclusion criteria:** Pregnant women with low‐risk pregnancies, living in Tehran, their first single pregnancy, and pregnancy demands for couples. **Exclusion criteria:** Not described. **Age [Mean (SD)]—Intervention, Control:** 26 (4.04), 26.53(5.03) **Occupation [*n* (%)]—Intervention, Control:** Housewife: 60 (93.8), 58 (90.8) Employee: 4 (6.2), 6 (9.2) **Education status [*n* (%)]—Intervention, Control:** 0–6 years (primary): 14 (21.6), 9 (13.8) 7–12 years (secondary): 36 (56.9), 43 (67.7) >12 years (college): 14 (21.5), 12 (18.5)
**Interventions**	**Intervention—Nutrition education (*n* = 64)** **Description:** At the beginning of the classes, a question‐and‐answer session was conducted for the initial survey of the women's knowledge. This was followed by a lecture, presentation of posters, photographs, and training pamphlets, and then continued with group discussions. All participants were followed up by the researcher, and a month later, both groups were re‐evaluated by questionnaires. Training classes included two sessions of approximately 2‐h duration (10–12 a.m.) (based on comfort and willingness of the participants), were held 1 week apart. **Nutrition counseling messaging:** Criteria considering healthy diet behaviors during pregnancy in the study and questionnaire and the educational contents of classes were prepared based on National Health Services (NHS) advice and National Institute for Health and Care Excellence (NICE) guidance. **Intervention adapted to local context:** Not reported. **Women empowerment approach:** Unclear empowerment approach. **Control (*n* = 64)** **Description:** After final evaluation, mothers in the control group were given 1 day of training and received an educational pamphlet.
**Outcomes**	No outcomes of interest reported
**Notes**	Funding: Not described

Risk of bias

**Table:** 

Bias	Author's judgment	Support for judgment
Risk of bias arising from the randomization process	Some concerns	Randomized allocation but no information on allocation concealment. There was no significant differences at baseline.
Risk of bias due to deviations from the intended interventions (effect of assignment to intervention)	Some concerns	No information on blinding, no information on deviations that arose.
Risk of bias due to deviations from the intended interventions (effect of adhering to intervention)	Some concerns	No information on blinding of Participants and people delivering the intervention.
Missing outcome data	Low risk	Overall, all 130 patients completed the study, and the response rate was 100%.
Risk of bias in measurement of the outcome	Low risk	Appropriate measure of outcome with same number of outcome measurements.
Risk of bias in selection of the reported result	Low risk	outcomes measured as stated in methods with defined tools.

Lin 2020

**Table:** 

**Methods**	Individual Randomized Controlled Trial in Women and Children Hospital, School of Medicine, Xiamen University, China
**Participants**	**Inclusion criteria:** Adult pregnant women aged 18 years or older who had at least one risk factor of GDM were included in this study. The risk factors of GDM were defined as follows: age ≥35 years, pre‐pregnancy body mass index (BMI) ≥25 kg/m^2^, family history of diabetes mellitus, history of PCOS, and history of GDM in a previous pregnancy. **Exclusion criteria:** Pregnant women were excluded if they had pre‐existing diabetes mellitus, multiple pregnancies, use of medication that influences glucose metabolism (e.g., steroids, b‐adrenergic agonists, and anti‐psychotic drugs), physical disability, or severe psychiatric disorders. **Age [Mean (SD)]—Intervention, Control:** 31.4 (4.9), 31.8 (5) **Parity [*n* (%)]—Intervention, Control:** 0: 89 (64), 93 (65.5) ≥1: 50 (36.0), 49 (34.5) **Education status [*n* (%)]—Intervention, Control:** 0–6 years (primary): 14 (21.6), 9 (13.8) 7–12 years (secondary): 36 (56.9), 43 (67.7) >12 years (college): 14 (21.5), 12 (18.5)
**Interventions**	**Intervention—Lifestyle intervention (counseling and recommended exercise) (*n* = 152)** **Description:** Participants in the intervention group received structured but individually modified education regarding a balanced dietary pattern, moderate physical activity, and weight control. The intervention included one face‐to‐face education session with an interventionist at the onset of treatment and continuous educational messages delivered via a WeChat public account at a frequency of twice per week. **Nutrition counseling messaging:** The balanced dietary pattern in the intervention was based on the China diagnosis and therapy guideline of pregnancy with diabetes Mellitus, aimed to achieve or maintain ideal body weight and meet nutritional needs. Pregnant women were encouraged to consume vegetables, fruits, high‐fiber whole‐grain products, low‐fat dairy products, and to avoid foods rich in sugar and saturated fatty acids, among other guidance. Participants in the intervention group were recommended to engage in approximately 30 min of moderate‐intensity physical activity at a frequency of three to four times per week. Bodyweight control during early and mid‐to‐late pregnancy was based on the recommendation of the National Academy of medicine. **Intervention adapted to local context:** Not reported. **Women empowerment approach:** Unclear empowerment approach. **Control (*n* = 152)** **Description:** Usual prenatal care was offered.
**Outcomes**	Gestational weight gain, Hemorrhage, Mode of delivery
**Notes**	Funding: This research received no specific grant from any funding agency in the public, commercial, or not‐for‐profit sectors.

Risk of bias

**Table:** 

Bias	Author's judgment	Support for judgment
Risk of bias arising from the randomization process	Low risk	Randomization was performed using computer‐generated randomization schedules.
Risk of bias due to deviations from the intended interventions (effect of assignment to intervention)	Some concerns	No information on blinding, no information on deviations that arose.
Risk of bias due to deviations from the intended interventions (effect of adhering to intervention)	Some concerns	No information on blinding of participants or deviations that arose.
Missing outcome data	Some concerns	Almost all the participants were analyzed.
Risk of bias in measurement of the outcome	Low risk	outcome measurement was appropriate and similar across groups. No information on outcome assessor's awareness of intervention but highly unlikely to influence measurement.
Risk of bias in selection of the reported result	Low risk	All outcomes reported appropriately.

Liu 2009

**Table:** 

**Methods**	Individual Randomized Controlled Trial in antenatal clinics, Wuhan & Macheng, China
**Participants**	**Inclusion criteria:** (1) healthy pregnant women; (2) at their third trimester; (3) had at least three routine examinations at these antenatal clinics. **Exclusion criteria:** Not reported **Age [Mean (SD)]—Intervention, Control:** <25: 47 (30.52), 47 (31.76) 25–30: 88 (57.14), 81 (54.73) >30: 19 (12.34), 20 (13.51) **Education [*n* (%)]—Intervention, Control:** Primary: 22 (14.29), 23 (15.54) Middle school: 73 (47.40), 70 (47.30) High school: 7 (17.53), 38 (25.68) College: 32 (20.78), 17 (11.48) **Occupation [*n* (%)]—Intervention, Control:** Laborer: 26 (16.88), 19 (12.84) Farmer: 61 (39.61), 74 (50.0) Technical: 10 (6.49), 4 (2.70) Government official: 11 (7.14), 6 (4.05) Trader: 4 (2.60), 3 (2.03) House duty: 42 (27.27), 42 (28.38)
**Interventions**	**Intervention—Nutrition counseling (*n* = 220)** **Description:** Women of the two intervention groups in both areas were informed to take part in two, 2‐h health and nutritional education sessions. The sessions were held twice a month to ensure that every participant in the intervention groups could join in. A guidebook concerning postpartum nutrition and healthcare knowledge which was compiled by our research team was disseminated to the intervention group women after class. **Nutrition counseling messaging:** The content of the two sessions focused on: – Food guide pyramid knowledge – Nutrient‐food association knowledge – The importance of dairy consumption, fruit and vegetable consumption – Examples of healthy menus – Optimal hygiene behavior and physical exercise patterns during postpartum period‚ – Discussions about healthy lifestyle during the postpartum period – Common misconceptions on hygiene behaviors during puerperium – Discussions about nutrition and health problems concerns postpartum practices **Intervention adapted to local context:** Not reported. **Women empowerment approach:** Unclear empowerment approach. **Control (*n* = 190)** **Description:** Routine examinations
**Outcomes**	Mode of delivery, Dietary intake during pregnancy (kcal/day)
**Notes**	Funding: Not reported

Risk of bias

**Table:** 

Bias	Author's judgment	Support for judgment
Risk of bias arising from the randomization process	High risk	Allocation concealment was not mentioned and there was a significant difference across education level (*p* = 0.001*) and household income across groups.
Risk of bias due to deviations from the intended interventions (effect of assignment to intervention)	Some concerns	participants were likely to be aware of the intervention as mothers who met the inclusion criteria were placed either in the intervention or comparison group according to their respective villages and were recruited on a rolling basis. However, there were no deviations from the trial context and appropriate analysis was conducted.
Risk of bias due to deviations from the intended interventions (effect of adhering to intervention)	Low risk	Participants could have possibly been aware of assigned intervention due to allocation method however there were no failures in implementing the intervention.
Missing outcome data	Some concerns	Data was missing for 105 participants which may have contributed to the baseline imbalance.
Risk of bias in measurement of the outcome	Low risk	Outcome measures were appropriate and assessors were independent for groups. there was probably no differnece in how the outcome was measured across groups.
Risk of bias in selection of the reported result	Low risk	All outcomes reported approprriety

Liu 2017

**Table:** 

**Methods**	Non‐randomized Controlled Study, Maternity outpatient clinic of a large tertiary hospital in Wuhan, China
**Participants**	**Inclusion criteria:** Inclusion criteria were primiparas at least 20 years of age, having a single pregnancy confirmed by ultrasound, over 20 weeks of gestation, willing to have a vaginal birth, a pre‐pregnancy BMI of 18.5–24.9 and understanding of the written Chinese language. **Exclusion criteria:** Pregnant women were excluded from the study if they were 1) over 35 years of age; 2) had pregnancy complications such as cardiovascular, digestive, endocrine and reproductive system diseases; 3) had multiple gestations; and 4) could not have a vaginal birth because of predisposing factors such as an abnormal pelvis, malposition, or uterine fibroids. **Age [Mean (SD)]—Intervention, Control:** 26.73 (2.67), 7.11 (2.31) **Occupation [*n* (%)]—Intervention, Control:** Office clerk: 13 (25), 15 (30) Technician: 2 (4), 1 (2) Freelance work: 5 (9), 7 (14) Unemployed: 4 (7), 3 (5) **Education level [*n* (%)]—Intervention, Control:** Junior high school: 3 (5), 2 (3) Senior high school: 4 (7), 2 (4) College: 7 (13), 6 (11) Bachelor or above: 10 (20), 14 (27) **Income level (Yuan/month) [*n* (%)]—Intervention, Control:** 1000–1999: 2 (3), 1 (2) 2000–2999: 7 (13), 6 (12) 3000–3999: 6 (11), 6 (12) 4000–4999: 5 (10), 4 (7) ≥5000: 4 (8), 6 (12)
**Interventions**	**Intervention—Lifestyle intervention (counseling and recommended exercise) (*n* = 50)** **Description:** The intervention was developed based on the Transtheoretical model (TTM) (Prochaska et al., 2002). The education intervention based on the TTM theory was provided after the basic education given to all women. Participants in the intervention group were assessed to determine their readiness for change to control their gestational weight gain during each prenatal visit between 20 and 30 weeks by asking questions congruent with each stage (Prochaska et al., 2002). The intervention Booklet of Health Management (BHM) during pregnancy was developed by the investigator before the study and distributed to the intervention group at the second time intervention. **Nutrition counseling messaging:** The BHM described the benefits and necessity of weight management, the dietary management (controlling food intake, meeting the nutrition needs during different pregnancy stages, keeping a balanced diet, preparing foods using portions from a food exchange, et al.) and included information on an exercise plan during pregnancy. The intervention booklets (BHM) was based on educational information found in current literature (Asbee et al., 2009; Bogaerts et al., 2013), dietary guidelines for Chinese pregnant women from the Chinese nutrition society (Chinese Nutrition Society, 2010) and suggestions by nutrition experts, health care professionals, and obstetric care experts from two tertiary hospitals in Wuhan. Maternal women suggestions were also gathered and considered before the present study. **Intervention adapted to local context:** Not reported. **Women empowerment approach:** Partial empowerment approach. **Control (*n* = 51)** **Description:** At the first prenatal check, the investigator provided routine health education about the effects of excessive gestational weight on pregnancy outcomes and explained the pattern of ideal weekly gain and overall maternal weight gain based on participants’ calculated BMI. The maternal health handbook was distributed as a medical record at the first pre‐natal check which recorded the weight of each prenatal visit, which was routine prenatal care.
**Outcomes**	Gestational weight gain, Mode of delivery, low birthweight
**Notes**	Funding: Not reported

Risk of bias

**Table:** 

Bias	Author's judgment	Support for judgment
Risk of bias arising from the randomization process	Some concerns	allocation was random but no information on concealment with no difference between groups at baseline
Risk of bias due to deviations from the intended interventions (effect of assignment to intervention)	Low risk	health care professionals provided routine checks for control group and provided counseling for intervention group.
Risk of bias due to deviations from the intended interventions (effect of adhering to intervention)	Some concerns	No information on blinding of participants or deviations that arose.
Missing outcome data	High risk	only 75% of participants have outcome data reported, with no reason provided for loss of follow up or analysis accounting for missing, more missing in the intervention group
Risk of bias in measurement of the outcome	Low risk	outcome measurement was appropriate and similar across groups.
Risk of bias in selection of the reported result	Low risk	All outcomes reported appropriety.

Malta 2021

**Table:** 

**Methods**	Non‐randomized Controlled Study, public primary health care in Botucatu, Sao Paulo State, Brazil
**Participants**	**Inclusion criteria:** Aged 18 years or more and in the first gestational trimester were invited to participate in the study. **Exclusion criteria:** Pregnant women who moved to antenatal care in private health services during the follow‐up, moved to another city, suffered abortion, presented conditions that classified them as having high‐risk pregnancies 21 or had any adverse condition that required rest or reduced physical activity 15 were excluded. **Age [*n* (%)]: ‐ Intervention, Control:** 18–19: 32 (17.7), 21 (12.2) 20–30: 107 (59.1), 103 (59.9) 30–more: 42 (23.2), 48 (27.9) **Maternal skin color, White [*n* (%)]: ‐ Intervention, Control:** 112 (62.9), 113 (65.7) **Maternal education (years) [*n* (%)]: ‐ Intervention, Control:** ≤8: 130 (71.2), 146 (84.9) <8: 51 (28.2), 26 (15.1) **Socioeconomic position [*n* (%)]: ‐ Intervention, Control:** ** Class B: 14 (8.0), 20 (11.8) Class C: 112 (63.6), 123 (72.8) Classes D/E: 50 (28.4), 26 (15.4) **Classified according to the Brazilian Association of Research Companies (ABEP. http://www.abep.org/, accessed on 10/May/2015)
**Interventions**	**Intervention (*n* = 185)** **Description:** The intervention was divided into two main components: (1) Training all of physicians and nurses responsible for antenatal care in family health units through an educational activity that lasted 16 h and consisted of an immersion course and three workshops. (2) The systematic promotion of diet and physical activity during antenatal visits. The women were encouraged to engage in leisure‐time walking five times per week or more for 30–40 min each and at moderate intensity. According to current international guidelines, for pregnant women to be considered active and therefore experience the resulting benefits, they must engage in a minimum of 150 min of moderate or vigorous physical activity per week 15. Of note, previously inactive pregnant women were instructed to start walking for a shorter period and with lower intensity and to progressively increase their activity to reach the recommendation. Considering the ecological model adapted from Bauman et al. and Giles‐Corti, the intervention focused on intra and interpersonal factors that act on behavioral changes. **Nutrition counseling messaging:** The pregnant women were counseled individually about the importance and benefits of 5 healthy dietary habits and were motivated to adopt these habits: consumption of 3 fruits daily; 2 portions of vegetables (one raw and one cooked) and 2 portions of beans (one at lunch and one at dinner) at least 5 days per week; and sporadic consumption of soft drinks and commercially prepared cookies. The selection of these 5 dietary habits was based on previous studies conducted in the same municipality 6,24,25. These recommendations were based on national guidance concerning diet during pregnancy 16 and in the national food guide. **Intervention adapted to local context:** Not reported **Women empowerment approach:** Partial empowerment model **Control (*n* = 177)** **Description:** These pregnant women received routine antenatal care in accordance with national recommendation
**Outcomes**	No outcomes of interest reported.
**Notes**	Funding: We acknowledge the staff at the Research Unit on Collective Health, Botucatu Medical School, Sao Paulo State University, the interviewers, the professionals from the Botucatu, Sao Paulo State primary health care network, and the pregnant women who made this study possible.

Risk of bias

**Table:** 

Bias	Author's judgment
Representativeness of the exposed cohort	Somewhat representative of the average population of pregnant women in the community*
Selection of the nonexposed cohort	Drawn from the same community as the exposed cohort*
Ascertainment of exposure	secure record (eg surgical records)*
Demonstration that outcome of interest was not present at start of study	Yes*
Comparability of cohorts based on the design or analysis	Does not control
Assessment of outcome	No description
Was follow‐up long enough for outcomes to occur	Yes*
Adequacy of follow up of cohorts	Complete follow up—all subjects accounted for*

Mazloomy‐Mahmoodabad 2020

**Table:** 

**Methods**	Individual Randomized Controlled Trial of Individual and group training that took place in the public health centers in Isfahan, Iran
**Participants**	**Inclusion criteria:** pregnant women age 13–42 entered the study from 6 to 12 weeks of gestation (9). Inclusion criteria include Weber's problems, weight loss (untreated thyroid disease), insulin‐dependent diabetes mellitus, drug‐dependent hypertension, type 1 or 7 diabetes mellitus, and Yabel diabetes, addiction, deficiencies and underlying problems, disease Ointment, drug use, kidney disease, anemia, body mass scales were more than 35 kg/m and following adherent diets (6). **Exclusion criteria:** Not reported. **Age [Mean (SD)]—Intervention, Control:** 22.3 (3.99), 22.83 (3/89)
**Interventions**	**Intervention—Lifestyle intervention (counseling and recommended exercise) (*n* = 88)** **Description:** In this session, with the face to face training, the related yruheh was trained in 1 h with the 5‐min waifs of lambs of pregnant maidens for 6–12 weeks, and the yruheh (4) was presented to each person with the weight and weight of the mass of body masses. Air charts were displayed in front of mothers in each training session, drawing and scores. The question‐and‐answer process of was repeatedly hampered by inclusive learning. In the training of the instructor, the Pender male pre‐test questionnaire was used. **Nutrition counseling messaging:** **Intervention adapted to local context:** Not reported. **Women empowerment approach:** Unclear empowerment approach. **Control (*n* = 90)** **Description:** Not reported
**Outcomes**	No outcomes of interested reported.
**Notes**	Funding: Not reported

Risk of bias

**Table:** 

Bias	Author's Judgment	Support for Judgment
Risk of bias arising from the randomization process	Low risk	Codes were used for randomization, but there are no baseline imbalances.
Risk of bias due to deviations from the intended interventions (effect of assignment to intervention)	Low risk	Double blinded study.
Risk of bias due to deviations from the intended interventions (effect of adhering to intervention)	Low risk	Double blinded study with no information on failing to implement intervention.
Missing outcome data	Low risk	Almost all the participants were analyzed.
Risk of bias in measurement of the outcome	Low risk	Outcome measures were conducted using Pender health promotion model. there was probably no difference in how the outcome was measured across groups.
Risk of bias in selection of the reported result	Low risk	All outcomes reported appropriately.

Mirmolaei 2009

**Table:** 

Methods	Non‐randomized Controlled Study in health centers in Semnan, Iran
**Participants**	**Inclusion criteria:** pregnant women age 13–42 entered the study from 6 to 12 weeks of gestation (9). Inclusion criteria include Weber's problems, weight loss (untreated thyroid disease), insulin‐dependent diabetes mellitus, drug‐dependent hypertension, type 1 or 7 diabetes mellitus, addiction, deficiencies and underlying problems, disease Ointment, drug use, kidney disease, anemia, body mass scales were more than 35 kg/m and following adherent diets (6). **Exclusion criteria:** Not reported. **Age [Mean (SD)]—Intervention, Control:** 22.3 (3.99), 22.83 (3/89) **Occupation [*n* (%)]—Intervention, Control:** Housewife: 130 (95.6), 108 (79.4) Employed: 6 (4.4), 28 (20.6) **Education [*n* (%)]—Intervention, Control:** Primary: 6 (4.4), 4 (2.9) Middle School: 23 (16.9), 19 (14) High school: 71 (52.2), 71 (52.2) academic education: 36 (26.5), 42 (30.9)
**Interventions**	**Intervention—Nutrition education (*n* = 136)** **Description:** Pregnant women in the experimental group, received routine gestational care at health centers, participated in nutrition training classes. During the two 90‐min sessions 1 week apart, nutritional training was provided to mothers in classes of 8–12 people. **Nutrition counseling messaging:** The content of these classes includes the importance of nutrition and nutrition during pregnancy, training of food groups and the amount of need of the pregnant woman for each, the volume and number of meals, good and bad food, good and bad food, as well as bad beliefs. Dinner and gastrointestinal problems were common during pregnancy and how they were related to nutrition. **Intervention adapted to local context:** Not reported. **Women empowerment approach:** Unclear empowerment approach. **Control (*n* = 136)** **Description:** Mothers in the control group received only routine gestational care.
**Outcomes**	No outcomes of interest reported.
**Notes**	Funding: Not reported

Risk of bias

**Table:** 

Bias	Author's judgment	Support for judgment
Risk of bias arising from the randomization process	High risk	Quasi‐randomized
Risk of bias due to deviations from the intended interventions (effect of assignment to intervention)	Some concerns	Unlikely to be double blind since intervention conducted in health center.
Risk of bias due to deviations from the intended interventions (effect of adhering to intervention)	Some concerns	No information on blinding of participants or deviations that arose.
Missing outcome data	Some concerns	There is no information on participants included in the study vs analyzed.
Risk of bias in measurement of the outcome	Low risk	Outcome measured using a questionnaire detailed in the methods, unlikely that the outcome assessors knwoledge would impact the assessment.
Risk of bias in selection of the reported result	Low risk	All outcomes reported as specified in the methods and analyzed appropriately.

Mituki 2017

**Table:** 

**Methods**	Cluster Randomized Controlled Trial in Kiandutu Health Centre, Thika, Kenya
**Participants**	**Inclusion criteria:** The inclusion criteria included being less than six (<6) months gestation; absence of chronic diseases such as hypertension, diabetes, and tuberculosis, babies born low birth weight, multiple births and mothers below 18 years. **Exclusion criteria:** Not reported **Age [Mean (SD)]—Intervention, Control:** 24.15 (23.99) **Ethnicity [*n* (%)]—Intervention, Control:** Kikuyu: 29.2 (70.8) Kamba: 30 (70) Luyha/Kisii: 20 (80) Others: 28.6 (71.4) **Employment [*n* (%)]—Intervention, Control:** Informal 24.7 (75.3) Formal 43.8 (56.2) Business 33.3 (66.7) None: 27.6 (72.4) **Education level [*n* (%)]—Intervention, Control:** ≤Primary: 8 (92) ≥ Primary: 38.5 (61.5) **Household income [*n* (%)]—Intervention, Control:** ≤11,700: 25.5, 74.5 ≥ 11,700: 31.5, 68.5 **Marital Status [*n* (%)]—Intervention, Control:** Married 21.8 (71.9) Single/separated/divorced 36.4 (63.6)
**Interventions**	**Intervention—Nutrition education (*n* = 176)** **Description:** All mothers who accepted to participate in the study filled an initial Socio‐demographic, a Household Food insecurity Access scale and KAP questionnaire. These tools assessed Socioeconomic, demographic, food security situation and intra‐partum factors. After the 34th week gestation all mothers filled the BSES‐SF, they thereafter filled the questionnaire at the 37th week gestation or during their last clinic visit and again after six (6) months post‐partum. **Nutrition counseling messaging:** Mothers in the intervention received personalized nutrition education geared towards improving their BSE at the health centers from nutritionists and were visited four times by community health workers (CHWs) before delivery. The CHWs encouraged mothers to exclusively breastfeed and answered questions raised by the mothers. **Intervention adapted to local context:** Not reported. **Women empowerment approach:** Partial empowerment model **Control (*n* = 256)** **Description:** Routine examinations
**Outcomes**	Intent to breastfeed
**Notes**	Funding: None

Risk of bias

**Table:** 

Bias	Author's judgment	Support for judgment
Risk of bias arising from the randomization process	High risk	Allocation concealment was not mentioned and there was a significant difference across education level (*p* = 0.001*) and household income across groups.
Risk of bias due to deviations from the intended interventions (effect of assignment to intervention)	Some concerns	participants were likely to be aware of the intervention as mothers who met the inclusion criteria were placed either in the intervention or comparison group according to their respective villages and were recruited on a rolling basis. However, there were no deviations from the trial context and appropriate analysis was conducted.
Risk of bias due to deviations from the intended interventions (effect of adhering to intervention)	Low risk	Participants could have possibly been aware of assigned intervention due to allocation method however there were no failures in implementing the intervention.
Missing outcome data	Some concerns	Data was missing for 105 participants which may have contributed to the baseline imbalance.
Risk of bias in measurement of the outcome	Low risk	Outcome measures were appropriate, and assessors were independent for groups. there was probably no difference in how the outcome was measured across groups.
Risk of bias in selection of the reported result	Low risk	All outcomes reported approprriety.

Mohsenzadeh‐Ledari 2020

**Table:** 

**Methods**	Individual Randomized Controlled Trial in Rouhani and yahyanzhed hospitals, Babol University of Medical Sciences, from Iran
**Participants**	**Inclusion criteria:** Inclusion criteria included written consent, Iranian nationality, gestational age less than 14 weeks, a waist circumference greater than 80 cm, metabolic syndrome with IDF criteria and the ability to reading and writing in Persian and to answer questionnaires without the help of another person. **Exclusion criteria:** Pregnancy complications, including the threat of abortion, placenta Previa, and hydatidiform mole, multiple pregnancies, and mental illnesses required medication or ongoing psychiatrist care, the emergence of midwifery problems and conditions that could endanger the continuation of the physical activity program, confirming the fetal malformations, fetal or neonatal death during the study were considered as exclusion criteria. **Age [Mean (SD)]—Intervention, Control:** 31.00 (6.01), 31.36 (5.22) **Occupation [*n* (%)]—Intervention, Control:** housewife: 53 (88.3), 51 (85.0) employed: 7 (11.7), 9 (15.0) **Education level [*n* (%)]—Intervention, Control:** University: 9 (15.0), 24 (40.0) Secondary school: 24 (40.0), 18 (30.0) Primary school: 27 (45.0), 18 (30.0) **Economic situation [*n* (%)]—Intervention, Control:** low: 12 (20.0), 21 (35.0) Middle: 43 (71.7), 34 (56.7) High: 5 (8.3), 5 (8.3)
**Interventions**	**Intervention—Nutrition education (*n* = 60)** **Description:** For each participant in the intervention group, in addition to performing an assigned intervention program along with routine pregnancy care, a 120‐min sole motivational counseling session was conducted by the researcher at the beginning of the study from week 15–20 of pregnancy. The intervention group was referred to the nutritionist twice (15‐20 weeks of pregnancy and then 20–24 weeks of pregnancy). The diets contain 20% protein, 30% fat, and 50% carbohydrate. Also, three sessions of intervention counseling were provided to receive intervention contents including physical activity for pregnancy such as pelvic floor muscle training, bodybuilding, muscle strengthening, stretching, relaxation and walking during 15, 20, and 32 weeks of gestation. In addition, were used a pamphlet and educational booklet, and educational CDs prepared by the Ministry of Health and Medical Education, Maternal Health Department for women's Health during Pregnancy and Postpartum. To follow up on the caring intervention, the researcher made a phone call with the participants in the intervention group every 10 days to 2 weeks. There were also forms for participants to record the number of sessions and the duration of their exercises. The researcher phone number was given to the participants to contact if they encountered any problems or possible complications. **Nutrition counseling messaging:** Nutrition advice and recommendations based on the Nutrition Guidelines ministry of Health for Pregnant Women to use five main nutrient groups including fruits, vegetables, grains, meat, dairy and water per day based on the Pregnant Mothers‚ Food Pyramid that is 15–20 kcal/kg daily for body mass above 30, 25 kcal/kg daily for body mass 25–30 and 30 kcal/kg daily for body mass between 20 and 25. **Intervention adapted to local context:** Not reported. **Women empowerment approach:** Partial empowerment model **Control (*n* = 60)** **Description:** The control group only received routine pre‐natal care.
**Outcomes**	Gestational weight gain
**Notes**	Funding: The present study was supported by Shahroud University of Medical Sciences. We hereby acknowledge the research deputy for grant No 9577and also thank the women who participated

Risk of bias

**Table:** 

Bias	Author's judgment	Support for judgment
Risk of bias arising from the randomization process	Low risk	Block randomization was used and allocation was conducted by random allocation software
Risk of bias due to deviations from the intended interventions (effect of assignment to intervention)	Some concerns	No information on blinding, no information on deviations that arose.
Risk of bias due to deviations from the intended interventions (effect of adhering to intervention)	Low risk	Participants could have possibly been aware of assigned intervention due to allocation meethod however there were no faliures in implementing the intervention
Missing outcome data	Low risk	data was missing for 9% of participants but the reasons reported in the flowchart are unlikely to depend on true value.
Risk of bias in measurement of the outcome	Low risk	outcome measurement was appropriate and similar across groups. No information on outcome assessor's awareness of intervention but highly unlikely to influence measurement.
Risk of bias in selection of the reported result	Low risk	The data were analyzed in accordance with a pre‐specified plan that was finalized before unblinded outcome data were available for analysis and unlikely to have been selected.

Nahrisah 2020

**Table:** 

**Methods**	Non‐randomized Controlled Study, two municipalities in the province of Aceh Indonesia
**Participants**	**Inclusion criteria:** **Exclusion criteria:** **Age [Mean (SD)]—Intervention, Control:** 28.4 (3.51), 28.2 (3.82) **Occupation [*n* (%)]—Intervention, Control:** Housewife: 67 (95.7), 65 (92.9) Working: 3 (4.3%), 5 (7.1) **Education [*n* (%)]—Intervention, Control:** 9th grades: 46 (65.7), 38 (54.3) 12th grades and higher: 24 (34.3), 32 (45.7)
**Interventions**	**Intervention (n =)** **Description:** Intervention for anemic pregnant women consisted of 2 home visits (45–60 min) from a well‐trained village government midwife. The period between the first home visit and the second home visit was 2 weeks. First home visit: individual education through the pictorial handbook. Second‐home visit: individual counseling. During the sessions, the midwife and women discussed how to improve the amount, variety, and frequency of iron‐rich food intake with consideration of price, preferences, and accessibility. Creating an atmosphere that supported IFA compliance, such as placing the tablet in an easily seen place, customizing the time of intake, asking the husband or a family member for a reminder and solving the IFA side‐effect problem. **Nutrition counseling messaging:** The information on IDA in the pictorial handbook consisted of definitions, signs and symptoms, causes and risk factors, and prevention and treatment, as well as foods rich in iron and recommendations on the intake of IFA tablets. There were pictures available to support the explanation, in addition to pictures of food rich in iron that are affordable, locally available, and recognized by the community. This visit emphasized the behaviors of iron‐rich food and IFA tablet intake, the benefits of and barriers to iron‐rich food and IFA tablet intake, how to cope with the barriers, and susceptibility to and severity of anemic pregnancy. The information and activities above were designed to fulfill the assumption of the HBM; that is, a person's beliefs about health are determinants of the possibility of the individual make changes in their lifestyle and behaviors. **Intervention adapted to local context:** The suitability of the handbook‚ that is, message content, literacy, pictures, and layout was evaluated through informal interviews with health professionals, including a doctor, a master of community nutrition, a Master of Health Education, and five village government midwives, selected for convenience. Due to the Islamic characteristics of society in Aceh, human figures in the book were depicted as Muslim and attached several quotes from the Quran in the handbook. **Women empowerment approach:** Partial empowerment model **Control (*n* = 78)** **Description:** On the other hand, anemic pregnant women in the control area received routine antenatal care without any further support such as that received by women in the intervention area.
**Outcomes**	No outcomes of interest reported.
**Notes**	Funding: This study was supported by the 90th Anniversary of Chulalongkorn University Scholarship under the theRatchadaphisek Somphot Fund, Chulalongkorn University. The authors thank the research teams from the health care offices at Kota Langsa and Kota Lhokseumawe municipalities, province of Aceh, Indonesia. Last, but not least, thanks to Mr. Fadlin Noer who designed the pictorial handbook.

Risk of bias

**Table:** 

Bias	Author's judgment	Support for judgment
Risk of bias arising from the randomization process	High risk	Quasi‐randomized
Risk of bias due to deviations from the intended interventions (effect of assignment to intervention)	Low risk	Unlikely to be double blind since intervention conducted in health canter. No deviations arose because of trial context.
Risk of bias due to deviations from the intended interventions (effect of adhering to intervention)	Low risk	Unlikely to be double blind since intervention conducted in health center. No participants lost due to adherence.
Missing outcome data	Low risk	Almost all the participants were included in the analysis, with no difference in dropouts between groups
Risk of bias in measurement of the outcome	Low risk	Outcomes were measured using standardized instruments.
Risk of bias in selection of the reported result	Low risk	All outcomes reported as specified in the methods and analyzed approporietly.

Nyamasege 2018

**Table:** 

**Methods**	Cluster Randomized Controlled Trial of home‐based nutritional counseling part of the maternal Infant and Young Child Nutrition (MIYCN) project by the African Population and Health Research Center, Kenya
**Participants**	**Inclusion criteria:** The inclusion criteria for each pregnant woman were that she resided in the Korogocho or the Viwandani slum, was aged 12 to 49 years, was registered within the NUHDSS, and provided informed consent. **Exclusion criteria:** The exclusion criteria from the study were women of reproductive age who were to deliver before the intervention started. **Age [*n* (%)]—Intervention, Control:** 14–19: 80 (16.6), 88 (16.9) 20–24: 209 (43.5), 212 (40.7) 25–29: 127 (26.4), 127 (24.4) 30–45: 64 (13.5), 94 (18.0) **Ethnicity [*n* (%)]—Intervention, Control:** Kikuyu: 137 (28.6), 136 (26.0) Luhya: 84 (17.5), 108 (20.7) Luo: 79 (16.5), 75 (14.4) Kamba: 93 (19.4), 105 (20.2) Others: 87 (18.0), 97 (18.7) **Occupation [*n* (%)]—Intervention, Control:** Unemployed: 406 (84.7), 481 (92.3) Self‐employed: 38 (7.9), 22 (4.2) Casual labor: 22 (4.5), 13 (2.5) Salaried: 14 (2.9), 5 (1.0) **Education [*n* (%)]—Intervention, Control:** Less than primary: 67 (14.1), 84 (16.1) Completed primary: 272 (56.6), 303(58.3) Secondary school: 119 (24.8), 114 (21.8) College/university: 22 (4.5), 20 (3.8) **Marital status [*n* (%)]—Intervention, Control:** Married/living together: 383 (79.8), 433 (83.2) Single: 60(11.6), 65 (13.5) Separated/divorced: 17 (3.6), 18 (3.5) Widowed: 3 (0.6), 2 (0.5) Missing: 12 (2.5), 6 (1.2)
**Interventions**	**Intervention—Home‐based counseling (*n* = 480)** **Description:** The CHVs had a minimum of primary school education and basic primary health care training from the Kenyan ministry of health. They were further trained using the community Infant and Young Child Feeding (IYCF) training package developed by United Nations Children's Fund (UNICEF)/WHO in 2006 and adopted by the government of Kenya. The trained CHVs passed down this information to the mothers primarily, but also to the fathers or other caregivers where possible in the intervention group. Counseling was initiated as soon as the mother was recruited, as early as possible during pregnancy, and then continued monthly till after 1 year following delivery. A total of seven home‐based, personalized nutrition‐counseling sessions were offered during pregnancy to each pregnant woman in the intervention group. The first 4 sessions were conducted once in every fourth week till the 34th week of gestation, while the other three sessions were d1 weekly till the mother gave birth. **Nutrition counseling messaging:** Key messages were adopted from the training package and highlighted in brightly colored IYCF counseling cards. These cards were used by the CHVs during counseling. The specific maternal nutrition education key messages included importance of adequate diet during pregnancy, attending ANC, and taking iron and folate supplements. Other maternal health‐related key messages were on seeking early treatment for infections and how to prevent them, encouraging the use of good hygienic practices, avoiding alcohol, smoking, and nonprescription drugs, and good antenatal care.19 The counseling schedule for CHVs is published in supplementary material by Kimani et al. **Intervention adapted to local context:** The CHVs had a minimum of primary school education and basic primary health care training from the Kenyan ministry of health. They were further trained using the community Infant and Young Child Feeding (IYCF) training package developed by United Nations Children‚ Fund (UNICEF)/WHO in 2006 and adopted by the government of Kenya. **Women empowerment approach:** Complete empowerment model **Control (*n* = 521)** **Description:** The control group received the usual ANC services, reading materials on MIYCN, and counseling visits on basic health care by the CHVs. The CHVs home visits are defined by the needs of the pregnant woman as a common practice specified under community health promotion strategies.18 These CHVs did not receive the additional training on MIYCN as the CHVs in the intervention group did.14
**Outcomes**	Mode of delivery, adherence to nutrition counseling, preterm birth, low birthweight
**Notes**	Funding: The primary study was funded by the Welcome Trust, Grant number (097146/Z/11/Z). High appreciation towards the core funding of APHRC from The William and Flora Hewlett Foundation; the Swedish International Cooperation Agency (SIDA); and funding for the NUHDSS from the Bill and Melinda Gates Foundation, where the primary study was nested.

Risk of bias

**Table:** 

Bias	**Author's judgment**	**Support for judgment**
Risk of bias arising from the randomization process	High risk	Allocation concealment was not mentioned and there was a significant difference across occupation (*p* = 0.023*) and parity across groups.
Risk of bias due to deviations from the intended interventions (effect of assignment to intervention)	Some concerns	No information on blinding, no information on deviations that arose.
Risk of bias due to deviations from the intended interventions (effect of adhering to intervention)	Some concerns	No information on blinding of participants or deviations that arose.
Missing outcome data	Low risk	All participants were included in the analysis.
Risk of bias in measurement of the outcome	Low risk	Outcome measures were conducted using standardized measures. No information on outcome assessor's awareness of intervention but highly unlikely to influence measurement.
Risk of bias in selection of the reported result	Low risk	Outcomes measured as stated in methods with defined tools.

Nyamasege 2021

**Table:** 

**Methods**	Cluster Randomized Controlled Trial of home‐based nutritional counseling part of the maternal Infant and Young Child Nutrition (MIYCN) project by the African Population and Health Research Center from 2012 to 2015.
**Participants**	**Inclusion criteria:** This was a cohort of pregnant women recruited during pregnancy and followed up quarterly with their children until the 13th month after delivery and at the 55th month. Inclusion criteria involved women of reproductive age, registered in the NUHDSS, who became pregnant in the year between 2012 and 2014, who voluntarily agreed to participate in the study. **Exclusion criteria:** mother‐child pairs who participated in the initial study but migrated out of the study area, presence of chronic diseases, hearing and vision disabilities and/or death of the index child. A total of 220 mother‐child pairs in the exposed (intervention group) and 218 in the unexposed (control group) were traced. **Age [Mean (SD)]—Intervention, Control:** 14–20: 222 (28.8), 223 (29.3) 21–30: 446 (57.9), 458 (60.3) 31–50: 102 (13.3), 79 (10) **Ethnicity [*n* (%)]—Intervention, Control:** Kikuyu: 200 (26.0), 217 (28.6) Luhya: 159 (20.7), 133 (17.5) Luo: 111 (14.4), 125 (16.5) Kamba: 156 (20.2), 148 (19.4) Other: 144 (18.4), 137 (18) **Occupation [*n* (%)]—Intervention, Control:** Unemployed: 561 (72.8), 544 (71.6) Casual labor: 95 (12.4), 97 (12.8) Own business: 92 (12.0), 95 (12.4) Salaried: 22 (2.8), 24 (3.2) **Religion [*n* (%)]—Intervention, Control:** Christian: 701 (91.0), 700 (92.2) Muslim: 65 (8.5), 40 (5.2) Other: 4 (0.5), 20 (2.6) **Education [*n* (%)]—Intervention, Control:** Preschool: 131 (17.0), 119 (15.6) Primary: 450 (58.4), 429 (56.5) Secondary: 171 (22.3), 186 (24.5) College: 18 (2.3), 26 (3.4) **Monthly income in Kenyan Shilling [*n* (%)]—Intervention, Control:** <4000: 136 (17.7), 170 (22.4) 4000–6999: 223 (29.0), 214 (28.2) >7000: 411 (53.3), 376 (49.4) **Marital status [*n* (%)]—Intervention, Control:** Married: 639 (83.0), 606 (79.7) Unmarried: 131 (17.0), 154 (20)
**Interventions**	**Intervention—Home‐based counseling (*n* = 2174)** **Description:** The intervention will involve personalized, home‐based counseling of pregnant women and mothers of infants on optimal MIYCN practices by CHWs Table 1. Additionally, consultations will be held with key organizations including the Division of Nutrition and the Division of Community Health Services in the Ministry of Health; UNICEF and other organizations working on MIYCN issues. For the intervention arm, CHWs will visit the pregnant woman about once every month up to week 34, after which they will visit the mother weekly until delivery. After delivery, they will visit the mother weekly in the first 1 month, then once a month until the end of infancy, but during the fifth month when mothers are expected to initiate complementary feeding, the frequency will be biweekly. All recruited pregnant women in both the intervention arm and control arm will receive information materials regarding MIYCN throughout the follow‐up period; these are normally provided by the Ministry of Health for distribution as part of the standard care. Additionally, the recruits will be provided with standard care counseling by CHWs, which will address antenatal and postnatal care, appropriate tests during pregnancy, health facility delivery, general nutrition, hygiene, and immunization. Seven nutrition counseling sessions were offered during pregnancy. The first four sessions were conducted once every 4 weeks until the 34th week of gestation, whilst the other three sessions were done on a weekly basis until delivery, see online supplementary material. **Nutrition counseling messaging:** The elements of counseling package are based on WHO/UNICEF IYCF guidance documents, training and other materials, including the Infant and Young child feeding counseling integrated course [32]. The package is designed to equip community workers or primary health care staff to be able to support mothers, fathers and other caregivers to optimally feed their infants and young children. The package will be adapted to include counseling messages on maternal nutrition. Counseling will be initiated during pregnancy as soon as the mother is recruited and will be continued until the end of infancy (1 year after delivery). Counseling will encompass maternal nutrition, immediate initiation of breastfeeding after birth, breast positioning and attachment, exclusive breastfeeding, frequency and duration of breastfeeding, expressing breast milk, storage and handling of expressing and lactation management. It will also focus on age‐appropriate complementary feeding, starting at 6 months: age‐appropriate complementary foods (nutritious, safe, affordable, and locally available), feeding frequency and quantity, and appropriate feeding practices including hygiene and responsive feeding behaviors, which encourage mother‐child interaction during feeding [33,34]. Though we will not seek to establish HIV status of the participants, mothers in the intervention arms will be advised on how they should feed the child in case a mother is HIV positive, while the information materials given to both intervention and control arms will also stipulate information on feeding for HIV‐exposed infants. **Intervention adapted to local context:** A formative study will be conducted before the roll‐out of the intervention to inform the design and components of the intervention including content of the counseling messages. This will involve a qualitative study involving interviews with key informants in the study communities including community leaders, CHWs, traditional birth attendants (TBAs) and health professionals; mothers (pregnant, breastfeeding and mothers of children under 5 years. **Women empowerment approach:** Partial empowerment model **Control (*n* = 2326)** **Description:** The control group received the usual antenatal care practices such as home‐based counseling from the CHWon usual care (antenatal care, family planning, skilled delivery and immunization), MIYCN reading materials. This CHW was not participating in the intervention and was not given any form of training on MIYCN(20).
**Outcomes**	Intent to breastfeed
**Notes**	Funding: This was a cohort of pregnant women recruited during pregnancy and followed up quarterly with their children until the 13th month after delivery and at the 55th month. The initial study‚Äôs inclusion criteria involved women of reproductive age, registered in the NUHDSS, who became pregnant in the year between 2012 and 2014, who voluntarily agreed to participate in the study.

Risk of bias

**Table:** 

Bias	Author's judgment	Support for judgment
Risk of bias arising from the randomization process	Some concerns	No information on randomization allocation, no differences between groups in baseline characteristics.
Risk of bias due to deviations from the intended interventions (effect of assignment to intervention)	Some concerns	No information on blinding, no information on deviations that arose.
Risk of bias due to deviations from the intended interventions (effect of adhering to intervention)	Some concerns	No information on blinding of participants or deviations that arose.
Missing outcome data	Low risk	Participants with missing information on child length/height were excluded from the data analysis. There were few cases (2·1%) of missing data regarding the outcome variable from the 2012–2015 follow‐up and no cases of missing data in the 2018 follow‐up.
Risk of bias in measurement of the outcome	Low risk	Method of measuring outcome was appropriate and similar between groups. Outcome assessors might have been aware of the intervention received but their knowledge could not have influenced by their knowledge.
Risk of bias in selection of the reported result	Low risk	Outcomes were reported using raw numbers and linear modeling.

Perichart‐Perera 2009

**Table:** 

**Methods**	Non‐randomized Controlled Study in The National Institute of Perinatology, Mexico City, Mexico
**Participants**	**Inclusion criteria:** *Gestational age‚ ≤29 weeks *Diagnosis of gestational diabetes subclass A2 or B1 *Diagnosis of pregestational type 2 diabetes *Two fasting AND two postprandial glucose values indicating mentioned diagnoses **Exclusion criteria:** Diagnosis of gestational diabetes subclass A1 Diagnosis of pregestational type 1 diabetes Renal, hepatic, or thyroid disorder Diabetes mellitus complications **Age [median (range)]—Intervention, Control:** 32.3 (22–42), 32.3 (19–43))
**Interventions**	**Intervention—Medical Nutrition Therapy (MNT) (*n* = 108)** **Description:** The MNT program consisted of individual nutrition counseling with an intensive education component performed by one clinical dietitian. The program included nutrition assessment, nutrition intervention, and capillary glucose self‐monitoring. Specific materials were designed for nutrition therapy and self‐monitoring education. 19 Women received a glucose meter (Optium MediSense, Abbott Laboratories, Bedford, MA) and strips to perform capillary blood glucose self‐monitoring 2 days a week, 6 times a day (before and 2 h after each meal). Fasting and 2 h postprandial serum glucose was also measured every 2 weeks by a glucose oxidase method. Fasting and 2 h postprandial glycemic goals were ≤95 mg/dL (5.27 mmol/L) and ≤120 mg/dL (6.66 mmol/L), respectively. Ketonuria was measured with urine strips on each visit (Multistix‐10SG, Bayer Diagnostics, Germany). If ketones were present and weight gain was subnormal, energy prescription was increased (200–300 kcal/day). If weight gain was adequate, energy was not modified and carbohydrates were increased (no more than 45%). Until the end of pregnancy, all women received follow‐up every 2 weeks by the dietitian and the endocrinologist, who was responsible for prescribing insulin, as needed, to meet glycemic goals. **Nutrition counseling messaging:** Nutrition recommendations were based on nutrition practice guidelines for gestational diabetes developed and published by the American Dietetic Association. **Intervention adapted to local context:** Not reported **Women empowerment approach:** Unclear empowerment approach **Control (*n* = 112)** **Description:** Monthly medical visits with the endocrinologist before 28 weeks of gestation, and every 2 weeks thereafter. Most women attended 1 initial nutrition orientation group session where they received dietary information from a technician.
**Outcomes**	Preterm birth, Low birthweight
**Notes**	Funding: Not reported

Risk of bias

**Table:** 

Bias	**Author's judgment**
Representativeness of the exposed cohort	Somewhat representative of the average population of pregnant women in the community*
Selection of the nonexposed cohort	Drawn from the same community as the exposed cohort*
Ascertainment of exposure	structured interview*
Demonstration that outcome of interest was not present at start of study	Yes*
Comparability of cohorts based on the design or analysis	Structured interview*
Assessment of outcome	Independent blind assessment*
Was follow‐up long enough for outcomes to occur	Yes*
Adequacy of follow up of cohorts	Complete follow up—all subjects accounted for*

Permatasari 2021

**Table:** 

**Methods**	Non‐randomized Controlled Study in Bogor Regency, Indonesia
**Participants**	**Inclusion criteria:** Pregnant women who lived at least 6 months in these villages were included in this study to maintain homogeneity in access to information exposure and health services regarding nutrition and reproductive health. Another inclusion criterion was a maximum gestational age of 27 weeks (end of the second trimester), because the intervention is intended to be implemented before the delivery period. **Exclusion criteria:** The exclusion criteria were confirmation or diagnosis of serious health problems requiring a special diet and nutritional needs, as well as premature delivery during the data collection period. **Age [*n* (%)]—Intervention, Control:** 19–25 years: 41 (42.3), 36 (37.1) 26–35 years: 43 (44.3), 4 (46.4) >35 years: 13 (13.4), 16 (16.5) **Residence status [*n* (%)]—Intervention, Control:** Original population: 76 (78.4), 77 (79.4) Migrants: 21 (21.6), 20 (20.6) **Occupation [*n* (%)]—Intervention, Control:** Housewife: 93 (95.9), 92 (94.8) Working Mothers: 4 (4.1), 3 (3.1) **The decision‐maker in the household [*n* (%)]—Intervention, Control:** Mother: 6 (6.2), 5 (5.2) Father and mother: 83 (85.6), 82 (84.5) **Education level [*n* (%)]—Intervention, Control:** Elementary school (≤6 years): 44 (45.4), 41 (42.3) Junior high school (7–9 years): 38 (39.1), 39 (40.2) Senior high school (9–12 years): 15 (15.5), 14 (14.4) College (>12 years): 0 (0.0), 3 (3.1) **Family income [*n* (%)]—Intervention, Control:** <1,500,000 IDR: 19 (19.6), 20 (20.6) 1,500,000‐3,000,000 IDR: 58 (59.8), 61 (62.9) > 3,000,000 IDR: 20 (20.6), 16 (16.5)
**Interventions**	**Intervention—Nutrition education (*n* = 107)** **Description:** They were placed in small groups (four or five mothers per group) and received 2 h of nutrition and reproductive health education from a facilitator every 2 weeks for 3 consecutive months. The educational contents and special applied strategies can be seen in Table 1. The education consisted of three sessions that included theoretical (lectures) and practical sessions. The first covered parenting (psycho‐emotional and nutritional parenting) and was complemented by role‐playing. The second covered nutrition during pregnancy, stunting, and immunity. This session was reinforced by the simulation to assess nutritional status and nutritional requirements for the first 1000 days of life. In this session, the facilitator used two packets of nutrition discs, one consisting of eight discs that determine the nutritional status of children based on age groups, and another consisting of eight discs focused on the needs of balanced nutrition from the gestational period through adolescence (19 years). The third session covered reproductive health education, equipped with the games of myths and facts. **Nutrition counseling messaging:** 1. Parenting ‐ Psycho‐emotional parenting ‐ Nutritional parenting Lectures (module, leaflet, flipchart) Role play (scenario, props, name tags, accessories for each participant), 35 min for lectures, 25 min for roleplay 2. Nutrition during pregnancy stunting, and immunity Lectures (module, leaflet, flipchart) Simulation (nutrition discs),15 min for lectures‚ 15 min for simulation 3. Reproductive health education Lectures (module, leaflet, flipchart), Games: myths and facts (list of questions, props), 15 min for lectures, 15 min for games Total = 120 min **Intervention adapted to local context:** **Women empowerment approach:** Partial empowerment model **Control (*n* = 112)** **Description:** The control group received the usual antenatal care practices such as home‐based counseling from the CHWon usual care (antenatal care, family planning, skilled delivery and immunization), MIYCN reading materials. This CHW was not participating in the intervention and was not given any form of training on MIYCN(20).
**Outcomes**	No outcomes of interest reported.
**Notes**	Funding: Not reported

Risk of bias

**Table:** 

Bias	Author's judgment	Support for judgment
Risk of bias arising from the randomization process	High risk	Quasi‐randomized
Risk of bias due to deviations from the intended interventions (effect of assignment to intervention)	High risk	Most pregnant women had low education and Socioeconomic level in the study area. Their literacy level might have been a barrier to access information regarding nutrition and reproductive health.
Risk of bias due to deviations from the intended interventions (effect of adhering to intervention)	High risk	Given that most of the pregnant women had had low education and Socioeconomic which could impede intervention effects, failure to implement the intervention is likely No approach was reported to evaluating adherence.
Missing outcome data	Low risk	All the women were included in the analysis.
Risk of bias in measurement of the outcome	Low risk	Outcome assessors were probably aware of the intervention group since they were the same people that provided the counseling and they received training for that purpose. However, it is unlikely that their knowledge influenced the assessment.
Risk of bias in selection of the reported result	Low risk	All outcomes reported as specified in the methods and analyzed appropriately.

Kian 2016

**Table:** 

**Methods**	Individual Randomized Controlled Trial in Hospitals affiliated with Tehran University of Medical Sciences, Iran
**Participants**	**Inclusion criteria:** Pregnant women with gestational diabetes were enrolled in the study who had a gestational age of 28‐36 weeks and were admitted at one of three selected hospitals affiliated with Tehran University of Medical Sciences due to high blood sugar or diabetes diagnosis in 2012–2013. Other inclusion criteria were gestational, being literate, and lack of any known physical or mental disease that makes trainings impossible. **Exclusion criteria:** Women who received diabetes training before entering the study were excluded. **Age [*n* (%)]—Control group, face‐to‐face training, training booklet** 25 (30.14), 25 (30.93), 25 (30.744) **Highschool education [*n* (%)]—Control group, face‐to‐face training, training booklet** 24 (57.10), 24 (57.1), 22 (52.40) **Average economic status [*n* (%)]—Control group, face‐to‐face training, training booklet** 27 (64.20), 32 (76.20), 27 (64.30)
**Interventions**	**Intervention—face‐to‐face training (*n* = 42)** **Description:** In the intervention group, two training sessions were held on 2 consecutive days and each session took about 40 min. Training items that were taught in the second session were included; nutrition, physical activity and exercise, insulin injection, and following‐up after the pregnancy. **Intervention—training booklet (*n* = 42)** In the other intervention group, all the items taught by face‐to‐face training method were distributed in the form of an educational booklet, to be studied by the patients. Up to a week after the birth, the mothers were contacted by the researcher via phone to answer the questions, the mothers‚ medical records were also used, and then, the record sheet was completed by the researcher. **Nutrition counseling messaging:** Training items mentioned in the first session of the training included; a definition of gestational diabetes, causes, side effects, individuals at risk, and control and treatment of gestational diabetes such as glycemic control. Thus, in all three hospitals, mothers received comprehensive guidance on how to feed themselves by a dietitian. **Intervention adapted to local context:** Not reported **Women empowerment approach:** Unclear empowerment approach **Control (*n* = 42)** **Description:** In this study, the control group received routine hospital care and education.
**Outcomes**	Mode of delivery
**Notes**	Funding: This article was as a part of MSc thesis in midwifery approved by Tehran University of Medical Sciences in 2013 (approval code number: 0192‐99‐21969) and sponsored by the Nursing and Midwifery Care Research Center, Tehran University of Medical Sciences.

Risk of bias

**Table:** 

Bias	Author's judgment	Support for judgment
Risk of bias arising from the randomization process	Some concerns	Randomized allocation but no information on allocation concealment. There was no significant differences at baseline.
Risk of bias due to deviations from the intended interventions (effect of assignment to intervention)	Some concerns	No information on blinding, no information on deviations that arose.
Risk of bias due to deviations from the intended interventions (effect of adhering to intervention)	Some concerns	No information on blinding of Participants and people delivering the intervention. Likely to be aware.
Missing outcome data	Low risk	Almost all the participants were analyzed.
Risk of bias in measurement of the outcome	Some concerns	No information on the data collection method as well as no information on blindness of outcome assessors.
Risk of bias in selection of the reported result	Some concerns	Analysis intentions are not available.

Saadatnia 2020

**Table:** 

**Methods**	Non‐randomized Controlled Study in comprehensive health centers in Kermanshah, Iran
**Participants**	**Inclusion criteria:** individuals with a gestational age of 6–10 weeks (first gestational care (and overweight), body mass 25–9/29) were selected. Inclusion criteria for studying single pregnancy, having literacy, being able to read and write, being able to speak Persian, suffering from underlying diseases (such as diabetes, hypertension, thyroid, liver, and heart diseases), not being addicted, and being pregnant. **Exclusion criteria:** Not reported. **Age [mean]—Intervention, Control** 27.91, 27.53 **Occupation [*n* (%)] Intervention, Control** Housewife: 35 (77.8), 37 (82.2) Employed: 10 (22.2), 8 (17.8) **Education [n(%)]—Intervention, Control** Undergraduate: 13 (28.9), 11 (31.1) Diploma: 22 (18.9), 21 (46.7) University: 10 (22.2), 10 (22.2) **Income [*n* (%)]—Intervention, Control** Less than 2 million tomans: 8 (17.8), 9 (20.2) 2–3 mil: 27 (60), 21 (46.7) More than 3 million: 10 (22.2), 12 (26.7)
**Interventions**	**Intervention—face‐to‐face training (*n* = 45)** **Description:** Counseling sessions based on the health belief model, including four group counseling sessions, one session per week and each session for 45–60 min in groups (five to ten people) based on the pamphlet prepared according to the health belief model by lecture method, PowerPoint presentation, group discussion, Q&A, Giving pamphlets and explaining the food pyramid poster with the help of a nutrition consultant, the control group received day‐to‐day pregnancy care, and 1 month after the intervention, the two groups completed the questionnaires again. **Nutrition counseling messaging:** Basic introduction to the general principles of mothers’ familiarity with overweight and mother's familiarity with nutritional behavior Nutrition and the food pyramid of obesity and its healthy side effects. Explain the complications and problems of obesity in pregnancy to increase sensitivity and perceived severity, increase the perception of pregnant women in addition to the risk of obesity, provide statistics on the complications of obesity, summarize counseling sessions and plan the next session. Investigating the benefits and barriers to performing healthy eating behaviors to increase perceived benefits and reduce perceived barriers, identify barriers. Physical skills to improve the diet, answering potential questions of clients and their ambiguities and problems in the implementation of educational techniques and methods, summarizing the entire counseling process. **Intervention adapted to local context:** Not reported **Women empowerment approach:** Unclear empowerment approach **Control (*n* = 45)** **Description:** received day‐to‐day pregnancy care, and 1 month after the intervention
**Outcomes**	No outcomes of interest reported
**Notes**	Funding: Not reported

Risk of bias

**Table:** 

Bias	Author's judgment	Support for judgment
Risk of bias arising from the randomization process	High risk	Quasi‐randomized
Risk of bias due to deviations from the intended interventions (effect of assignment to intervention)	Low	Unlikely to be double blind since intervention conducted in health center. No deviations arose because of trial context.
Risk of bias due to deviations from the intended interventions (effect of adhering to intervention)	Some concerns	Unlikely to be double blind since intervention conducted in health center. No information on number of participants included in study and follow up till end of study.
Missing outcome data	Some concerns	There is no information on participants included in the study vs analyzed.
Risk of bias in measurement of the outcome	Low risk	Outcome assessment was conducted with a questionnaire therefore it is unlikely to have been influenced by the outcome assessors.
Risk of bias in selection of the reported result	Low risk	All outcomes reported as specified in the methods and analyzed appropriately.

Sachdeva 1994

**Table:** 

**Methods**	Non‐randomized Controlled Study in Subsidiary Health Cetre (SHC), Ludhiana, India
**Participants**	**Inclusion criteria:** individuals with a gestational age of 6–10 weeks (first gestational care (and overweight), body mass 25–9/29) were selected. Inclusion criteria for studying single pregnancy, having literacy, being able to read and write, being able to speak Persian, suffering from underlying diseases (such as diabetes, hypertension, thyroid, liver and heart diseases), not being addicted and being pregnant. **Exclusion criteria:** Not reported. **Age [mean]—Intervention, Control** 27.91, 27.53 **Occupation [*n* (%)]—Intervention, Control** Housewife: 35 (77.8), 37 (82.2) Employed: 10 (22.2), 8 (17.8) **Education [*n* (%)]—Intervention, Control** Undergraduate: 13 (28.9), 11 (31.1) Diploma: 22 (18.9), 21 (46.7) University: 10 (22.2), 10 (22.2) **Income [*n* (%)]—Intervention, Control** Less than 2 million tomans: 8 (17.8), 9 (20.2) 2–3 mil: 27 (60), 21 (46.7) More than 3 million: 10 (22.2), 12 (26.7)
**Interventions**	**Intervention—face‐to‐face training (*n* = 45)** **Description:** Counseling sessions based on the health belief model, including four group counseling sessions, one session per week and each session for 45–60 min in groups (five to ten people) based on the pamphlet prepared according to the health belief model by lecture method, PowerPoint presentation, group discussion, Q&A, Giving pamphlets and explaining the food pyramid poster with the help of a nutrition consultant, the control group received day‐to‐day pregnancy care, and 1 month after the intervention, the two groups completed the questionnaires again. **Nutrition counseling messaging:** Basic introduction to the general principles of mothers’ familiarity with overweight and mother's familiarity with nutritional behavior Nutrition and the food pyramid of obesity and its healthy side effects. Explain the complications and problems of obesity in pregnancy to increase sensitivity and perceived severity, increase the perception of pregnant women in addition to the risk of obesity, provide statistics on the complications of obesity, summarize counseling sessions and plan the next session. Investigating the benefits and barriers to performing healthy eating behaviors to increase perceived benefits and reduce perceived barriers, identify barriers. Physical skills to improve the diet, answering potential questions of clients and their ambiguities and problems in the implementation of educational techniques and methods, summarizing the entire counseling process. **Intervention adapted to local context:** Not reported **Women empowerment approach:** Unclear empowerment approach **Control (*n* = 45)** **Description:** received day‐to‐day pregnancy care, and 1 month after the intervention
**Outcomes**	Adherence to Iron supplement consumption
**Notes**	Funding: Not reported

Risk of bias

**Table:** 

Bias	Author's judgment
Representativeness of the exposed cohort	Somewhat representative of the average population of pregnant women in the community*
Selection of the nonexposed cohort	Drawn from the same community as the exposed cohort*
Ascertainment of exposure	Structured interview*
Demonstration that outcome of interest was not present at start of study	Yes*
Comparability of cohorts based on the design or analysis	Structured interview*
Assessment of outcome	Independent blind assessment*
Was follow‐up long enough for outcomes to occur	Yes*
Adequacy of follow up of cohorts	Complete follow up ‐ all subjects accounted for*

Sharifirad 2013

**Table:** 

**Methods**	Non‐randomized Controlled Study in Urban health care centers, Gonabad, Iran
**Participants**	**Inclusion criteria:** Target population in this study was pregnant women who were resided in Gonabad and went to urban health care centers for prenatal care. So, 110 pregnant women (case group: 54, control group: 56) were selected who had come to these centers in the first stage of prenatal care (6th‐10th week of pregnancy) in 1388 and were classified in case and control group. **Exclusion criteria:** Not reported. **Age [Mean (SD)]—Intervention, Control** 26.62 (5.62), 27.72 (5.15) **Occupation [*n* (%)]—Intervention, Control** Homemaker: 81.48 (44), 73.21 (41) Employee: 7.41 (4), 14.29 (8) Teacher: 11.11 (6), 12.5 (7) **Education [*n* (%)]—Intervention, Control** Uneducated: 3.70 (2), 3.57 (2) Up to Diploma: 61.11 (33), 58.93 (33) University degree: 35.19 (19), 37.5 (21)
**Interventions**	**Intervention—Nutrition education program (*n* = 54)** **Description:** Before the educational intervention, a questionnaire was completed by a female researcher in both groups via an organized interview in the first stage of prenatal care. Then the interview based on HBM was performed in two sessions of nutritional education (16 min for each session) according to nutrition educational Guidelines in health care centers and live lecture, group discussion, Colloquy and individual nutrition consultation for the experimental group. In the first session of the education plan, all case partners were invited to have active participation. In this way, two sessions of nutritional education (16 min for each session) was done. **Nutrition counseling messaging:** Not reported **Intervention adapted to local context:** Not reported **Women empowerment approach:** Unclear empowerment approach **Control (*n* = 56)** **Description:** In the control group, current education without using the educational model for nutrition during pregnancy in health care centers was performed.
**Outcomes**	Gestational weight gain
**Notes**	Funding: This study financially was supported by Gonabad University of Medical Sciences, Iran. The authors deeply are thankful to participants, Gonabad health care staff and health promotion, social development research center for their support.

Sunuwar 2019

**Table:** 

**Methods**	Non‐randomized Controlled Study in Tribhuvan University Teaching Hospital, Nepal
**Participants**	**Inclusion criteria:** Pregnant women aged 15–49 years who were willing to participate with mild and moderate anemia were included in the study. **Exclusion criteria:** Pregnant women who were vegetarian and with special medical conditions like diabetes mellitus, hypertension, ante partum hemorrhage, renal disease, malignancy, cardio‐pulmonary disease, hypo or hyperthyroidism, epilepsy and severe anemia were excluded from the study. **Age [Mean (SD)]—Intervention, Control** 26.23 (4.74), 26.06 (4.98) **Occupation [*n* (%)]—Intervention, Control** House wife: 32 (60.4), 27 (50.0) Employee: 12 (22.6), 12 (22.2) Trader: 6 (11.3), 8 (14.8) Unemployed: 0, 2 (3.7) Others: 3 (5.7), 5 (9.3) **Religion [*n* (%)]—Intervention, Control** Hindu: 42 (79.2), 42 (77.8) Buddhist: 8 (15.1), 6 (11.1) Others: 3 (5.7), 6 (11.2) **Education [*n* (%)]—Intervention, Control** No education: 1 (1.9), 4 (7.4) Literate only: 6 (11.3), 1 (1.9) Primary (1–5): 2 (3.8), 2 (3.7) Secondary (6–9): 10 (18.9), 13 (24.1) SLC and above: 34 (64.2), 34 (63.0)
**Interventions**	**Intervention—Nutrition education program (*n* = 58)** **Description:** Before the educational intervention, a questionnaire was completed by a female researcher in both groups via an organized interview in the first stage of prenatal care. Then the interview based on HBM was performed in two sessions of nutritional education (16 min for each session) according to nutrition educational Guidelines in health care centers and live lecture, group discussion, Colloquy and individual nutrition consultation for the experimental group. In the first session of the education plan, all case partners were invited to have active participation. In this way, two sessions of nutritional education (16 min for each session) was done. **Nutrition counseling messaging:** Not reported **Intervention adapted to local context:** Not reported **Women empowerment approach:** Unclear empowerment approach **Control (*n* = 57)** **Description:** In the control group, current education without using the educational model for nutrition during pregnancy in health care centers was performed.
**Outcomes**	Gestational weight gain, Hemoglobin concentration
**Notes**	Funding: DRS received funding by University Grants Commission (UGC), Nepal for this research (Award number: MRS/74‐75/S&T‐55 (www.ugcnepal.edu.np). The funders had no role in study design, data collection and analysis, decision to publish, or preparation of the manuscript

Tu 2021

**Table:** 

**Methods**	Individual Randomized Controlled Trial in East Hospital of the Sixth People's Hospital Affiliated to Shanghai Medical College, Shanghi, China.
**Participants**	**Inclusion criteria:** BMI before pregnancy ≥24 kg/m^2^, Gestational age less than 12 weeks. Age is 30–40 years old, singleton pregnancy. **Exclusion criteria:** suffer from chronic diseases before pregnancy, such as diabetes, hypertension. Multiple pregnancy **Age [Mean (SD)]—Intervention, Control** 26.23 (4.74), 26.06 (4.98) **Occupation [*n* (%)] Intervention, Control** House wife: 32 (60.4), 27 (50.0) Employee: 12 (22.6), 12 (22.2) Trader: 6 (11.3), 8 (14.8) Unemployed: 0, 2 (3.7) Others: 3 (5.7), 5 (9.3) **Religion [*n* (%)]—Intervention, Control** Hindu: 42 (79.2), 42 (77.8) Buddhist: 8 (15.1), 6 (11.1) Others: 3 (5.7), 6 (11.2) **Education [*n* (%)]—Intervention, Control** No education: 1 (1.9), 4 (7.4) Literate only: 6 (11.3), 1 (1.9) Primary (1–5): 2 (3.8), 2 (3.7) Secondary (6–9): 10 (18.9), 13 (24.1) SLC and above: 34 (64.2), 34 (63.0)
**Interventions**	**Intervention—Nutrition education program (*n* = 100)** **Description:** The intervention group carried out one‐to‐one nutrition intervention on the basis of pregnancy nutrition classes, including individualized nutrition demonstration meals, guided diet and exercise, and WeChat diet management. **Nutrition counseling messaging:** (1) Individual nutrition demonstration meal Food choices: (1) Cereals and potatoes: whole grains and miscellaneous beans account for 33%: you can choose starch‐rich potatoes, sweet potatoes, pumpkin, yams etc., but need to exchange equal calories with the staple food; try to avoid the intake of refined added sugar. (2) Vegetables: 300~500 g/day, using the “3‐2‐1 vegetable model” proposed by G SHeng, director of the Nutrition Department of the Sixth People's Hospital Affiliated to Shanghai Jiatong University, to ensure vegetable intake and variety; among them, “3” refers to 3 taels (150 g) of leafy vegetables, “2” refers to 2 taels (100 g) of melons and fruits, and “1” refers to 1 tael (50 g) of bacteria and algae. (3) Fish, poultry, eggs, meat and soybeans: lean poultry and livestock meat 50~75 g/day, fish and shrimp 50~75 g/day, eggs 50 g/day, soybeans 20 g/day. (4) Milk: 300~500 mL/day of milk (5) Cooking oil: 20~25 g/day, olive oil, linseed oil, etc can be selected. (6) Iodized table salt: <6 g/day (7) healthy snacks: You can choose the right amount of sugar‐free yogurt, nuts 10 g/day or low glycemic index (GI) fruits 200 g. meal allocation: Morning, lunch, and evening meals account for 25%, 30%, 30% of energy; 1 to 2 extra meals a day, each time accounting for 5% to 10% of the total energy is appropriate to help prevent excessive hunger. Cooking method: avoid frying, deep‐frying, and grilling. Steam, boil, quick stir, etc. are recommended. The order of eating: vegetables first, fish and shrimp meat, and staple food last. (2) WeChat diet management Inquire about diet and upload photos of meals every week through WeChat. according to the standard of nutritional demonstration meals, evaluate the diet structure, food choices, meal distribution and cooking methods, and give suggestions to adjust the diet in time. It is recommended that the pregnant women without exercise contradictions (such as placenta previa, threatened premature delivery, etc.) perform the moderate‐intensity exercise for 30‐40 min, such as brisk walking, 30 min after each meal. **Intervention adapted to local context:** Not reported **Women empowerment approach:** Unclear empowerment approach **Control (*n* = 100)** **Description:** The Control group received a group regular health education in the form of pregnancy nutrition classroom, the main method was text combined with pictures.
**Outcomes**	No outcomes of interest reported.
**Notes**	Funding: Not reported

Risk of bias

**Table:** 

Bias	Author's judgment	Support for judgment
Risk of bias arising from the randomization process	Low risk	Randomization block was used to enroll study subjects, no differences between groups in baseline characteristics.
Risk of bias due to deviations from the intended interventions (effect of assignment to intervention)	Some concerns	No information on blinding, no information on deviations that arose.
Risk of bias due to deviations from the intended interventions (effect of adhering to intervention)	Some concerns	No information on blinding of participants or deviations that arose.
Missing outcome data	Low risk	Almost all the participants were included in the analysis, with no difference in dropouts between groups.
Risk of bias in measurement of the outcome	Low risk	Method of measuring outcome was appropriate and similar between groups. Outcome assessors might have been aware of the intervention received but their knowledge could not have influenced by their knowledge.
Risk of bias in selection of the reported result	Low risk	outcomes measured as stated in methods.

Villalon 2010

**Table:** 

**Methods**	Non‐randomized Controlled Study in prenatal clinics in the Cotonou region, Africa
**Participants**	**Inclusion criteria:** These Beninese women were between 19 and 30 years old and were in the first month of gestation when recruiting for the study. They attended one of the prenatal clinics in the Cotonou region, did not consume any form of tobacco and had never experienced a previous pregnancy. **Exclusion criteria:** Not reported. **Age [Range]—Intervention, Control** 19–30 **Occupation [*n* (%)]—Intervention, Control** Urban: 18 (94.7), 26 (100) Rural: 1 (5.3), 0 (0) **Race/ethnicity [*n* (%)]—Intervention, Control** Fon: 13 (68.4), 10 (37.0) Yoruba: 0 (0), 5 (18.5) Gun: 1 (5.3), 3 (11.1) Adja: 1 (5.3), 5 (18.5) Dendi: 1 (5.3), 0 (0) Autres: 3 (15.8), 4 (14.8) **Religion [*n* (%)]—Intervention, Control** Catholic: 15 (78.9), 13 (52) Protestant: 2 (10.5), 6 (24) Muslim: 1 (5.3), 2 (8) Others: 1 (5.3), 4 (16) **Social capital [*n* (%)]—Intervention, Control** Single parent: 2 (11), 7 (26) Biparentale: 17 (89), 20 (74) **Matrimonial situation [*n* (%)]—Intervention, Control** Married: 7 (42.1), 12 (44.4) Engaged: 7 (36.8), 6 (22.2) Concubinage: 3 (15.8), 4 (14.8) Divorcee: 1 (5.3), 2 (7.4) Widow: 0 (0), 3 (11.1)
**Interventions**	**Intervention—Nutrition program (*n* = 20)** **Description:** The nutritional program developed consisted of two components, namely an assessment of the nutritional status of women at their 12th week of gestation and nutritional education. The latter consisted of individual nutritional advice including a personalized diet adapted to the needs of each woman and educational group sessions. Food intakes were also assessed during pregnancy to ensure that they were meeting nutritional needs. Finally, five educational group sessions were carried out with the women during the first and second trimesters of pregnancy. Various subjects were taught such as physiological transformations related to pregnancy, recommended diet, essential nutrients, body weight, breastfeeding, the introduction of solid foods to the child and diet. the nursing woman. **Nutrition counseling messaging:** The latter consisted of individual nutritional advice including a personalized diet adapted to the needs of each woman and educational group sessions. Various subjects were taught such as physiological transformations related to pregnancy, recommended diet, essential nutrients, body weight, breastfeeding, the introduction of solid foods to the child and diet. the nursing woman. **Intervention adapted to local context:** A prenatal nutrition program adapted to the Beninese context was implemented for the women. **Women empowerment approach:** Unclear empowerment approach **Control (*n* = 27)** **Description:** Not described.
**Outcomes**	Gestational weight gain
**Notes**	Funding:

Risk of bias

**Table:** 

Bias	Author's judgment	Support for judgment
Risk of bias arising from the randomization process	High risk	Quasi‐randomized
Risk of bias due to deviations from the intended interventions (effect of assignment to intervention)	Low risk	Unlikely to be double blind, probably no deviations arose because of trial context. An appropriate analysis was used to estimate the effect of assignment to intervention.
Risk of bias due to deviations from the intended interventions (effect of adhering to intervention)	Some concerns	No information on blinding of participants or deviations that arose.
Missing outcome data	Low risk	All the women were included in the analysis.
Risk of bias in measurement of the outcome	Low risk	Outcome measures were subjective measures such as weight and waist circumference, therefore knowledge of the group wouldn't influence outcome assessment. Assessment method was similar across groups.
Risk of bias in selection of the reported result	Low risk	All outcomes reported as specified in the methods and analyzed appropriately.

Vítolo 2011

**Table:** 

**Methods**	Individual Randomized Controlled Trial in Center Health Reference Unit, Brazil
**Participants**	**Inclusion criteria:** Pregnant women who were in prenatal care at the time of the study, during the period from January 2007 to May 2008, were invited to participate in the research, provided they met the following inclusion criteria: they had a gestational age between 10 and 29th weeks; belonged to the prenatal care group of the health unit. **Exclusion criteria:** Exclusion criteria were pregnant women with a positive HIV test, the previous diagnosis of diabetes, hypertension, anemia or any other condition that required a special diet and aged over 35 years. **Age [Range]—Intervention, Control** 19–30 **Occupation [*n* (%)]—Intervention, Control** Paid work: 59 (37.8), 58 (30.2) UNPAID: 97 (62.2), 111 (69.8)
**Interventions**	**Intervention—Dietary intervention (*n* = 159)** **Description:** Pregnant women in the Intervention Group received an interview one more month after receiving the instructions for their reinforcement. **Nutrition counseling messaging:** Pregnant women randomized to the Intervention Group received dietary guidelines in the first interview, summarized in eight to ten eating behaviors, with the aim of adjusting the speed of weight gain and improving the quality of food. For pregnant women with low weight, it was adopted as a priority to increase the energy density of the diet with the addition of a spoon of oil in the main meals, consume two snacks a day with high energy value (with examples of portions), 100 g of offal once a week and one fruit daily. For the eutrophic pregnant women, they were instructed to divide their food into six times a day, daily portions of vegetables, fruits and water; restrict the consumption of high‐fat foods and oil in preparations. For pregnant women with excess weight, intervals between meals (3–4 h) were prioritized; do not repeat food portions from meals and snacks; restrict the daily consumption of soft drinks and sweets, processed foods rich in fat and also the oil in preparations. Daily portions of vegetables, vegetables and fruits were determined. All guidelines provided values and recommended household food measures to facilitate understanding of the actions to be taken. **Intervention adapted to local context:** A prenatal nutrition program adapted to the Beninese context was implemented for the women. **Women empowerment approach:** Unclear empowerment approach **Control (*n* = 162)** **Description:** The Control Group did not receive dietary guidelines, but were informed about their nutritional status, and were instructed to carry out prenatal care, informing the physician of the result of the nutritional assessment.
**Outcomes**	Gestational weight gain
**Notes**	Funding: Not reported

Risk of bias

**Table:** 

Bias	Author's judgment	Support for judgment
Risk of bias arising from the randomization process	Low risk	No information on allocation concealment, no differences between groups in baseline characteristics.
Risk of bias due to deviations from the intended interventions (effect of assignment to intervention)	Some concerns	No information on blinding, no information on deviations that arose.
Risk of bias due to deviations from the intended interventions (effect of adhering to intervention)	Some concerns	No information on blinding of participants or deviations that arose.
Missing outcome data	Low risk	Almost all the participants were included in the analysis, with no difference in dropouts between groups.
Risk of bias in measurement of the outcome	Low risk	Method of measuring outcome was appropriate and similar between groups. Outcome assessors might have been aware of the intervention received but their knowledge could not have influenced by their knowledge.
Risk of bias in selection of the reported result	Low risk	All outcomes reported appropriety.

Zhou 2020

**Table:** 

**Methods**	Individual Randomized Controlled Trial in West China Second University Hospital, Sichuan University/West China Women and Children Hospital, China
**Participants**	**Inclusion criteria:** According to the diagnostic methods and standards of IADPSG2010, fasting plasma glucose (FPG) ≤5.1 mmol/L when first examined at 24–28 weeks of pregnancy could be diagnosed as GDM [6]. **Exclusion criteria:** (1) Twin or multiple pregnancies; (2) combined with other metabolic diseases such as thyroid dysfunction, abnormal secretion of adrenaline or adrenocorticotropic hormone or growth hormone; (3) combined with other diseases. **Age [Mean (SD)]—Intervention, Control** 29.6 (3.2), 28.5 (3.1) **Occupation [*n* (%)]—Intervention, Control** Paid work: 59 (37.8), 58 (30.2) UNPAID: 97 (62.2), 111 (69.8)
**Interventions**	**Intervention—Nutritional nursing (*n* = 50)** **Description:** (1) Prenatal care: evaluate the dietary preference of pregnant women and implement the individualized nutritional intervention. Then, experts from the Nutrition Department and Endocrinology Department explained the diet plan to pregnant women and their families and informed them of scientific methods of food collocation based on the results of the consultation, so as to maintain balanced nutrition. (2) Intrapartum care: Continuous observation of the labor process changes and evaluation of vaginal delivery status. After the uterine mouth opened to 2 cm, the pregnant woman was sent to the delivery room for one‐on‐one midwifery, and the pregnant woman was instructed to relax. Before delivery, a B‐ultrasound examination was carried out. Besides, the appropriate position should be selected according to the fetal position, and a venous channel was created electrocardiogram (ECG) monitoring of mother and fetus was done, and the first‐aid kit were prepared. Shortening of the second stage of labor was done as much as possible [9, 10]. During this period, it was immediately converted to cesarean section if the risk of vaginal delivery increased, and emergency preparations for cesarean section were made in advance. (3) Postpartum care: After birth, the umbilical cord blood was collected appropriately to measure the blood glucose, bilirubin, and other indicators. The infants were nursed as high‐risk infants and were placed in an incubator. At the same time, oxygen inhalation and warmth were given. 30 min later, breastfeeding was started and glucose solution was dripped. At the same time, the physical signs and complex‐ion were observed [11]. Then, 24 h after giving birth, insulin treatment at 50% of the original dose on the parturient woman was conducted. Then, the dose of insulin was adjusted and an evaluation of physical signs and the symptoms of the parturient woman was made. Any abnormalities were checked in time. At the same time, the parturient woman was urged to rest [12]. In addition, keeping the perineum clean was a priority. After delivery, the diet was formulated by a nutritionist to guide the parturient to have a high protein and high vitamin diet during the month. At the same time, an appropriate exercise plan was made according to the recovery of the parturient to enhance their health. It was also suggested to get out of bed as soon as possible to promote lochia excretion and train the uterine involution function [13, 14]. **Nutrition counseling messaging:** The daily caloric requirement in the first trimester of pregnancy was 105 kJ/Kg, and was 126 kJ/kg in the second and third trimester, in which protein accounted for 25%, carbohydrates accounted for 55% and fat accounted for 20% of the die, which was consumed in 5‐6 meals a day. In addition, pregnant women adhered to low salt and low sugar diets and were supplemented with folic acid appropriately during pregnancy [7, 8]. **Intervention adapted to local context:** Not reported. **Women empowerment approach:** Unclear empowerment approach **Control (*n* = 50)** **Description:** Routine nursing was implemented for the pregnant women in the control group and the main nursing measures are listed as follows: construct a favorable rehabilitation environment for pregnant women; nursing staff closely observed the indicators of pregnant women, such as blood glucose, blood pressure detection and infection, etc., and conducted psychological counseling for pregnant women regularly, so as to reduce the psychological barriers and negative emotions of pregnant women, and to achieve a more comfortable state [7, 8].
**Outcomes**	Hemorrhage
**Notes**	Funding: None

Risk of bias

**Table:** 

Bias	Author's judgment	Support for judgment
Risk of bias arising from the randomization process	Low risk	No information on allocation concealment, no differences between groups in baseline characteristics
Risk of bias due to deviations from the intended interventions (effect of assignment to intervention)	Some concerns	Nurses seem to be aware of the intervention group since intervention group consisted of additional individualized nutritional care in addition to the standard care in control group. No deviations reported
Risk of bias due to deviations from the intended interventions (effect of adhering to intervention)	Some concerns	Nurses seem to be aware of the intervention group since intervention group consisted of additional individualized nutritional care in addition to the standard care in control group
Missing outcome data	High risk	number of participants from eligibility to analysis not reported.
Risk of bias in measurement of the outcome	Low risk	Method of measuring outcome was appropriate and similar between groups. Outcome assessors might have been aware of the intervention received but their knowledge could not have influenced by their knowledge.
Risk of bias in selection of the reported result	Low risk	All outcomes reported appropriately.

ZiyendaKatenga‐Kaunda 2020

**Table:** 

**Methods**	Cluster Randomized Controlled Trial in Health facilities, Namkumba area in Mangochi, Southern Malawi.
**Participants**	**Inclusion criteria:** We recruited all consenting primi‐ and multiparous pregnant women between their 9th and 16th gestational week who were available during the study period and planned to give birth at the health facilities within the study area. **Exclusion criteria:** We excluded women carrying multiple fetuses and those with severe illnesses. **Age [Median (IQR)]—Intervention, Control** 24 (19–30), 23 (18–28) **Education [*n* (%)]—Intervention, Control** Illiterate/primary: 79 (83.7), 79 (78.6) Secondary/above: 13 (16.3), 24 (21.4) **Socioeconomic status [*n* (%)]—Intervention, Control** Very poor: 31 (33.7), 29 (28.2) Poor: 39 (42.4), 43 (41.7) Well‐off: 22 (23.9), 31(30.1) **Marital status [*n* (%)]—Intervention, Control** Single: 9 (4.3), 2 (16.5) Married: 83 (95.7), 91 (83.5)
**Interventions**	**Intervention—Nutrition education (*n* = 121)** **Description:** The intervention promoted the consumption of locally produced nutrient‐dense food. These foods were not always readily accessible, and many families had to buy them. The intervention promoted practical ways of addressing challenges relating to food accessibility, that is, the use of food powders of inaccessible foods to optimize the dietary diversity of the meals. The intervention also promoted the consumption of adequate amounts of food through the following comprehensive dietary recommendations: (i) diverse food groups in the main meals as well as in supplementary meals; (ii) frequent meals and snacks (at least five eating times per day); (iii) double the usual portions of relish (i.e. vegetables, legumes, meats, fish and nuts); (iv) Fe supplements; (v) fruits; (vi) vitamin‐C‐rich fruit following a meal with beans and (vii) 50 ml of milk (fresh goat milk or from cow milk powder) at least twice a week. The intervention also promoted absorption of these micronutrients through use of partially fermented whole grains and soaking beans overnight before cooking to reduce the concentration of phytate and polyphenols that inhibit absorption of Fe, Zn and Ca and the use of oils with green leafy vegetables and fruits to promote absorption of vitamins. **Nutrition counseling messaging:** The nutrition education constitutes summarized information of the recommended food groups to be consumed with no provision of cooking demonstrations. The intervention group was exposed to a more expanded and detailed dietary counseling and education in addition to the one provided through standard antenatal care. The specific intervention counseling and education was informed by the findings from a cross‐sectional pre‐study performed among pregnant women in the same study area (12). These findings implied to recommend inclusion of more fats, vitamin C‐rich foods and milk in the diet. Through linear programming, we identified possible food combinations which could increase the intakes of most micronutrients (28). The intervention delivery included monthly nutrition education sessions, followed by cooking demonstrations and weekly individualized counseling sessions. **Intervention adapted to local context:** Not reported. **Women empowerment approach:** Complete empowerment model **Control (*n* = 136)** **Description:** The control group was exposed to the standard antenatal health education given in Malawi.
**Outcomes**	No outcomes of interest reported.
**Notes**	Funding: This project was funded by the University of Oslo, The Global Health and Vaccination Program (GLOBVAC) of the ResearchCouncil of Norway and by the Throne Holst Foundation. The funders had no role in the design, analysis or writing of this article.

Risk of bias

**Table:** 

Bias	Author's judgment	Support for judgment
Risk of bias arising from the randomization process	Low risk	The villages were assigned STATA generated random numbers to allocate them to either the intervention or the control group, generating 10 clusters in each study group. The women from these clusters were identified by the study team in their communities and referred to the nearest designated local health Center where their pregnancy and the gestational age were confirmed and where they were also invited to consent to participate in the study.
Risk of bias due to deviations from the intended interventions (effect of assignment to intervention)	High risk	There is no information on whether the participants and carers were aware of the intervention groups
Risk of bias due to deviations from the intended interventions (effect of adhering to intervention)	High risk	There is no information on whether the participants and carers were aware of the intervention groups as well as no information on adherence.
Missing outcome data	High risk	There is no information on the missingness or follow up of participants from eligibility to analysis.
Risk of bias in measurement of the outcome	High Risk	There is no information who assessed the outcomes or details on the intervention to inform the possibility of the intervention being influenced by external factors.
Risk of bias in selection of the reported result	Some concerns	There is no information on the planned analysis of the authors and the framework used to develop the questionnaire.

**Characteristics of ongoing studies**

Chowdhury 2016

**Table:** 

**Methods**	Protocol for two arm parallel community‐based cluster randomized controlled trial in villages of Sherpur district, Bangladesh
**Participants**	**Inclusion criteria:** married and of reproductive age (15–49 years) ‚ pregnant with a duration of gestation of 7–12 weeks‚ and permanent residents of the study area. **Exclusion criteria:** We will exclude women who have:‚ planned to deliver outside the study area‚ been diagnosed with any chronic diseases, such as diabetes, hypertension and other diseases that may im‐pact on their ability to participate in the trial.
**Interventions**	**Intervention—A balanced plate nutrition education with practical demonstrations** **Description:** The education session has two parts: one‐to‐one nutrition‐specific counseling and practical demonstration. Each pregnant woman will have 4–7 sessions throughout her entire pregnancy. The sessions will last for approximately 30–45 min. Practical demonstration refers to exhibiting the meal preparation in front of the pregnant women and their family members in a participatory way. **Nutrition counseling messaging:** nutritional messages are developed with reference to the current Bangladeshi recommendations, which incorporates the following: (a) intake of food‐yielding at least 2500 kcal energy per day; (b) consumption of diversified food; (c) inclusion of animal‐sourced food in the diet, at least two servings per day; and (d) eating at least five times a day (five different plates for five meals) as follows: 2. Eat 1.5 dishes of rice or 3 pieces (medium‐sized) of chapaties, 1 dish of vegetables, 1 egg or 1 dish of thick lentil in the morning. 3. Eat 1 piece or 1 dish of seasonal fruit(s) and 1 dish of milk product(s) as a morning snack. 4. Eat 3 dishes of rice, 1 dish of vegetables, 1 dish of thick lentil and 1 piece of fish or meat or egg at lunch. 5. Eat 1 glass of milk, 1 dish of puffed rice mixed with molasses and 1 piece or 1 dish of seasonal fruit(s) in the evening snack. **Intervention adapted to local context:** Not reported. **Women empowerment approach:** Partial empowerment model **Control** **Description:** receive standard nutrition education.
**Outcomes**	The primary outcome of this study is the birthweight of the newborn infants.
**Notes**	Funding: MC conceived the overall study and wrote the first draft of the protocol and this manuscript and CRG critically reviewed it. MJD provided critical input regarding study design, sample size calculation, and outcome evaluation and statistical analysis plan. AA provided crucial input on formative research and process evaluation design and contributed to addressing the reviewers‚ Äô comments and revision of the paper. MC and CRG obtained funding for the intervention. All authors critically reviewed and approved the final version of the manuscript and agree to be accountable for all investigations necessary to resolve questions related to the accuracy or integrity of all or any part of the work.

Kirkwood 2021

**Table:** 

**Methods**	Protocol for cluster randomized controlled trial in two subdistricts of Sirajganj, Ullapara and Kamarkhanda, in northern Bangladesh
**Participants**	**Inclusion criteria:** All married women between 15 and 49 years, who are permanent residents of the study area and provide consent to participate and test positive with pregnancy urine test kit (Excel) (whose gestational age is ≤90 days), are eligible to participate in the SCC Trial. **Exclusion criteria:** We excluded any potential villages if there are any other maternal and or neonatal interventions either by the government or nongovernment organizations in the area. We also excluded villages where access is challenging, for example, in flood‐prone areas.
**Interventions**	**Intervention—Nutrition counseling and an unconditional cash transfer** **Description:** (1) nutrition BCC delivered on a specially tailored app on a smart‐phone (audio, video and animation); (2) direct nutrition counseling from a call center; and (3) unconditional cash transfer of 1000 Taka (US$12.50) received monthly via BKash mobile banking app. The study will provide thirty‐six 20‐min fortnightly counseling sessions. **Nutrition counseling messaging:** The counselors will use a nonjudgmental and empathetic disposition on mother's perceptions. They will try to increase perceived susceptibility to and seriousness of a health condition by sharing information on the consequences of malnutrition on maternal and child health. They will also stimulate positive behavior change by sharing knowledge of positive practices as a cue to action, highlighting motivational factors (sharing efficacy of positive nutrition practices in improving child growth and development) and enabling decision making through enhanced self‐efficacy (empowerment through information sharing). The counselors from Soi Pushti Sheba will use a life‐course approach, from pre‐conception and throughout the first 2 years of the child's life. The counselor will mainly focus on diet and micronutrient during pregnancy, on breastfeeding, and Infant and Young Child Feeding Practices (IYCF) **Intervention adapted to local context:** Not reported. **Women empowerment approach:** Complete empowerment model **Control** **Description:** The control arm will receive a mobile phone and the current government of Bangladesh health and nutrition services.
**Outcomes**	Difference in women's empowerment scores in the intervention arm as compared with the control arm of the SCC Trial. Secondary study outcomes will include (1) control over income and economic resources, (2) input and decision‐making power in nutrition and healthcare choices, and (3) experience and attitude towards IPV
**Notes**	Not reported

Malhotra 2013

**Table:** 

**Methods**	Abstract rural projects of Integrated Child Development Services (ICDS) scheme in Low‐income settings in Delhi, India
**Participants**	**Inclusion criteria:** All married women between 15 and 49 years, who are permanent residents of the study area and provide consent to participate and test positive with pregnancy urine test kit (Excel) (whose gestational age is ≤90 days), are eligible to participate in the SCC Trial. **Exclusion criteria:** We excluded any potential villages if there are any other maternal and or neonatal interventions either by the government or nongovernment organizations in the area. We also excluded villages where access is challenging, for example, in flood‐prone areas.
**Interventions**	**Intervention—Nutrition counseling and an unconditional cash transfer** **Description:** (1) nutrition BCC delivered on a specially tailored app on a smart‐phone (audio, video and animation); (2) direct nutrition counseling from a call center; and (3) unconditional cash transfer of 1000 Taka (US$12.50) received monthly via BKash mobile banking app. The study will provide thirty‐six 20‐min fortnightly counseling sessions. **Nutrition counseling messaging:** The counselors will use a nonjudgmental and empathetic disposition on mother's perceptions. They will try to increase perceived susceptibility to and seriousness of a health condition by sharing information on the consequences of malnutrition on maternal and child health. They will also stimulate positive behavior change by sharing knowledge of positive practices as a cue to action, highlighting motivational factors (sharing efficacy of positive nutrition practices in improving child growth and development) and enabling decision making through enhanced self‐efficacy (empowerment through information sharing). The counselors from Soi Pushti Sheba will use a life‐course approach, from pre‐conception and throughout the first 2 years of the child's life. The counselor will mainly focus on diet and micronutrient during pregnancy, on breastfeeding, and Infant and Young Child Feeding Practices (IYCF)**Intervention adapted to local context:** Not reported. **Women empowerment approach:** Complete empowerment model **Control** **Description:** The control arm will receive a mobile phone and the current government of Bangladesh health and nutrition services.
**Outcomes**	difference in women's empowerment scores in the intervention arm as compared with the control arm of the SCC Trial. Secondary study outcomes will include (1) control over income and economic resources, (2) input and decision‐making power in nutrition and healthcare choices, and (3) experience and attitude towards IPV
**Notes**	Not reported

Upadhyay 2013

**Table:** 

**Methods**	Insert information here
**Participants**	No vertical lines, only horizontal.
**Interventions**	The table may be used with gray background/bold text on top instead of to the left.
**Outcomes**	
**Notes**	

Munyogwa 2021

**Table:** 

**Methods**	Protocol for a cluster randomized trial in the peri‐urban areas of Dodoma City, the capital of the Dodoma region and the national capital of Tanzania
**Participants**	**Inclusion criteria:** Pregnant women at second trimester pregnancy (gestational age from 13 weeks and above) living in peri‐urban areas of Dodoma City at the time of data collection and willing to participate will be included in the study. **Exclusion criteria:** Pregnant women with special medical conditions, such as epilepsy, sickle cell anemia, diabetes mellitus, hypo‐ or hyperthyroidism, malignancy disease, hypertension, human immunodeficiency virus, antepartum hemorrhage, renal disease and severe anemia will be excluded from the study.
**Interventions**	**Intervention—Nutrition education** **Description:** Nutrition education will be delivered by a research team member using a developed education material. The education material (see Supporting Information: File 1) has been developed by a research team after doing an intensive literature review from previous studies [26, 27]. The developed educational material has been modified and translated to the Swahili language to suit our environment. Each pregnant woman in the interventional group will receive nutritional education. Nutrition education consists of the following: definition of anemia, symptoms of anemia, causes for anemia, effects of anemia, behavioral and dietary risks for developing anemia and prevention of anemia. Nutritional education will be delivered during all three phases of the study. The first phase will be delivered at the time of baseline study where a hard copy of education material will be provided to each participant for self‐learning. The second phase will be followed 1 month after the first, and the last phase will be carried out 1 month after the second phase. Hard copy of training material will be provided only during the first phase (baseline survey), but the same education material will be used to train pregnant women about nutritional education for all three phases of the study. The nutritional education training will be provided in groups of 10–11 women. Sessions will be interactive and are expected to last for 45–60 min. All participants in the interventional and control arms will receive one tablet of FEFO (200 mg of dried Ferrous Sulphateþ0.25 mg Folic Acid) supplement once daily as per ANC national guidelines [28] during the entire course of the study period. The FEFO supplements will be given on a monthly dose basis starting at the baseline survey. **Nutrition counseling messaging:** Nutrition education consists of the following: definition of anemia, symptoms of anemia, causes for anemia, effects of anemia, behavioral and dietary risks for developing anemia and prevention of anemia. **Intervention adapted to local context:** Not reported. **Women empowerment approach:** Complete empowerment model **Control** **Description:** Supplementation of FEFO tablets, malaria prophylaxis, de‐worming
**Outcomes**	The primary outcome of this study is Hemoglobin concentration.
**Notes**	Funding: This work is supported by the University of Dodoma through the programme of Junior Academic Staff Research Proposal (JAS) grand award. The grant will also assist the data collection process. The funders had no role in study design, data collection and analysis, decision to publish or preparation of the manuscript.

**Characteristics of excluded studies**

**Table:** 

Study	Notes
**Abrha** 2020	Exclusion reason: Wrong intervention;
**Abujilban** 2018	Exclusion reason: Not interactive counseling;
**Huang** 2017	Exclusion reason: Wrong setting;
**Adhikari** 2009	Exclusion reason: Not interactive counseling;
**Akter** 2012	Exclusion reason: Wrong study design;
**Althuizen** 2012	Exclusion reason: Wrong setting;
**Asbee** 2009	Exclusion reason: Wrong setting;
**Asiabar** 2018	Exclusion reason: Duplicate;
**Bogaerts** 2012	Exclusion reason: Wrong setting;
**Caut** 2019	Exclusion reason: Wrong study design;
**CostadeOliveira 2018**	Exclusion reason: Not interactive counseling;
**CTRI/2015/06/005834,15/06/005834 2015**	Exclusion reason: Wrong intervention;
**Delfiani 2021**	Exclusion reason: Not interactive counseling;
**DellaLibera 2011**	Exclusion reason: Wrong study design;
**Demilew** 2020	Exclusion reason: Duplicate;
**Demilew** 2020	Exclusion reason: Duplicate;
**Deveer** 2013	Exclusion reason: Not interactive counseling;
**Diddana** 2018	Exclusion reason: Wrong intervention;
**dosSantos 2019**	Exclusion reason: Wrong patient population;
**Fan** 2010	Exclusion reason: Wrong intervention;
**Fayasari** 2019	Exclusion reason: Wrong patient population;
**Fernald** 2016	Exclusion reason: Wrong patient population;
**Ferrari** 2020	Exclusion reason: Wrong intervention;
**Garmendia** 2015	Exclusion reason: Wrong setting;
**Garmendia** 2018	Exclusion reason: Wrong setting;
**Garmendia** 2020	Exclusion reason: Wrong setting;
**Gila‐Diaz** 2021	Exclusion reason: Wrong study design;
**Gomes** 2019	Exclusion reason: Wrong intervention;
**Guelinckx** 2010	Exclusion reason: Wrong setting;
**Guelinckx** 2010	Exclusion reason: Wrong setting;
**Hajian** 2020	Exclusion reason: Wrong intervention;
**Haruna** 2017	Exclusion reason: Wrong setting;
**Haruna** 2017	Exclusion reason: Wrong setting;
**Hui** 2011	Exclusion reason: Wrong setting;
**Hunt** 1976	Exclusion reason: Wrong setting;
**Ilmonen** 2011	Exclusion reason: Wrong setting;
**IRCT2017100736626N1,17100736626N1** 2017	Exclusion reason: Wrong setting;
**ISRCTN61793947,793947** 2018	Exclusion reason: Wrong setting;
**javadi 2020**	Exclusion reason: Wrong control group;
**Jindal** 2021	Exclusion reason: Wrong study design;
**Kaleem** 2020	Exclusion reason: Nutrition counseling not described;
**Kamudoni** 2016	Exclusion reason: Wrong study design;
**Kimani‐Murage** 2013	Exclusion reason: Wrong patient population;
**Kimani‐Murage** 2015	Exclusion reason: Wrong patient population;
**Kurnit** 2015	Exclusion reason: Wrong setting;
**Lim** 2016	Exclusion reason: Wrong study design;
**Lin** 2017	Exclusion reason: Wrong patient population;
**Luoto** 2009	Exclusion reason: Wrong setting;
**Meng** 2021	Exclusion reason: Wrong setting;
**Messito** 2020	Exclusion reason: Wrong setting;
**Mshanga** 2019	Exclusion reason: Not assessing the effectiveness of nutrition counseling;
**Mujsindi** 2014	Exclusion reason: Wrong setting;
**Mwangi 2020**	Exclusion reason: Wrong patient population;
**Nair** 2020	Exclusion reason: Wrong study design;
**NCT00792480,792480** 2008	Exclusion reason: Wrong setting;
**NCT02574767,574767** 2015	Exclusion reason: Wrong setting;
**NCT04275622,275622** 2020	Exclusion reason: Wrong setting;
**NCT04694235,694235** 2021	Exclusion reason: Wrong study design;
**Nguyen** 2017	Exclusion reason: Duplicate;
**Nguyen** 2017	Exclusion reason: Wrong patient population;
**Nguyen** 2021	Exclusion reason: Wrong patient population;
**Nikiema** 2017	Exclusion reason: Wrong patient population;
**Nimbalkar** 2017	Exclusion reason: Duplicate;
**Nugraheni** 2020	Exclusion reason: Wrong patient population;
**Nyamasege** 2019	Exclusion reason: Duplicate;
**Okesene‐Gafa** 2019	Exclusion reason: Wrong setting;
**Owais** 2017	Exclusion reason: Wrong patient population;
**PACTR202007617885299,2007617885299 2020**	Exclusion reason: Duplicate;
**Parra** 2005	Exclusion reason: Wrong study design;
**Pawalia** 2020	Exclusion reason: Wrong intervention;
**Peccei** 2017	Exclusion reason: Wrong setting;
**Piirainen** 2006	Exclusion reason: Wrong setting;
**RBR‐5jy777** 2019	Exclusion reason: Wrong intervention;
**RBR‐7yx36h** 2019	Exclusion reason: Nutrition counseling not described;
**Rifayanto** 2021	Exclusion reason: Wrong study design;
**Saadatnia** 2021	Exclusion reason: Nutrition counseling not described;
**Sahran** 2021	Exclusion reason: Wrong intervention;
**Santos** 2013	Exclusion reason: Wrong study design;
**Sartorelli** 2020	Exclusion reason: Duplicate;
**Scherbaum** 2017	Exclusion reason: Wrong patient population;
**Selvakumar 2016**	Exclusion reason: Wrong study design;
**Seshan** 2018	Exclusion reason: Wrong setting;
**Shimpuku** 2019	Exclusion reason: Wrong study design;
**Sirajuddin** 2018	Exclusion reason: Nutrition counseling not described;
**Soliman** 2019	Exclusion reason: Duplicate;
**Stewart** 2020	Exclusion reason: Wrong patient population;
**Sun** 2016	Exclusion reason: Nutrition counseling not described;
**TCTR20210317002,210317002** 2021	Exclusion reason: Nutrition counseling not described;
**Teweldemedhin** 2021	Exclusion reason: Duplicate;
**Thaver** 2020	Exclusion reason: Wrong study design;
**Todd** 2019	Exclusion reason: Wrong study design;
**Vahamiko** 2013	Exclusion reason: Wrong setting;
**Vesco** 2014	Exclusion reason: Wrong setting;
**Wijaya‐Erhardt** 2014	Exclusion reason: Wrong study design;
**Wolff** 2008	Exclusion reason: Wrong setting;

## SUMMARY OF FINDINGS TABLE


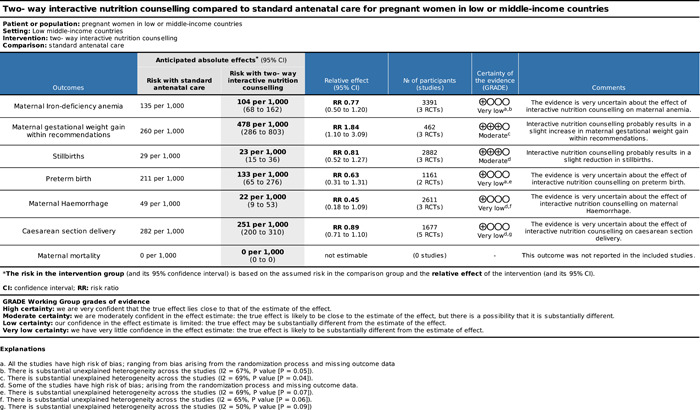


## SOURCES OF SUPPORT

**Internal sources**

- No sources of support provided

**External sources**

- Nutrition International, Canada.

This project is funded by Nutrition International.

## Supporting information

Supporting information.

## Footnotes

1 The presence of agency and opportunity structure‐related activities was not be used as inclusion criteria, but rather, was used to conduct sub‐group analyses comparing nutrition counseling interventions that do and do not apply a complete empowerment model.
